# Activation of IGF1R/p110β/AKT/mTOR confers resistance to α-specific PI3K inhibition

**DOI:** 10.1186/s13058-016-0697-1

**Published:** 2016-04-05

**Authors:** Cedric Leroy, Pedro Ramos, Karen Cornille, Debora Bonenfant, Christine Fritsch, Hans Voshol, Mohamed Bentires-Alj

**Affiliations:** 1 Friedrich Miescher Institute for Biomedical Research, Maulbeerstraße 66, 4058 Basel, Switzerland; 2 Novartis Institutes for Biomedical Research, Postfach, CH-4002 Basel, Switzerland

**Keywords:** Phosphatidylinositol 3-kinase, p110α, p110β, Resistance, Breast cancer

## Abstract

**Background:**

The PI3K pathway is hyperactivated in many cancers, including 70 % of breast cancers. Pan- and isoform-specific inhibitors of the PI3K pathway are currently being evaluated in clinical trials. However, the clinical responses to PI3K inhibitors when used as single agents are not as efficient as expected.

**Methods:**

In order to anticipate potential molecular mechanisms of resistance to the p110α isoform-selective inhibitor BYL719, we developed resistant breast cancer cell lines, assessed the concomitant changes in cellular signaling pathways using unbiased phosphotyrosine proteomics and characterized the mechanism of resistance using pharmacological inhibitors.

**Results:**

We found an increase in IGF1R, IRS1/IRS2 and p85 phosphorylation in the resistant lines. Co-immunoprecipitation experiments identified an IGF1R/IRS/p85/p110β complex that causes the activation of AKT/mTOR/S6K and stifles the effects of BYL719. Pharmacological inhibition of members of this complex reduced mTOR/S6K activation and restored sensitivity to BYL719.

**Conclusion:**

Our study demonstrates that the IGF1R/p110β/AKT/mTOR axis confers resistance to BYL719 in *PIK3CA* mutant breast cancers. This provides a rationale for the combined targeting of p110α with IGF1R or p110β in patients with breast tumors harboring *PIK3CA* mutations.

**Electronic supplementary material:**

The online version of this article (doi:10.1186/s13058-016-0697-1) contains supplementary material, which is available to authorized users.

## Background

The phosphatidylinositol 3-kinase (PI3K) signaling cascade is a major pathway inducing hallmarks of cancer [1]. Of the three main classes of lipid kinases in the PI3K family, the class I enzymes are often altered in human cancers [2]. Class IA PI3Ks include regulatory and catalytic subunits where the regulatory p85 maintains the catalytic p110 in a low activity state [2]. p110α and p110β are expressed ubiquitously whilst p110δ is restricted to immune cells. Class IA PI3Ks primarily generate phosphatidylinositol-3,4,5-trisphosphate (PIP3) from phosphatidylinositol-4,5-bisphosphate (PIP2), leading to the recruitment of PDK1 and AKT and activation of downstream kinases essential for cell growth, proliferation, survival, and metabolism [3, 4].

An estimated 70 % of breast cancers show hyperactivation of the PI3K pathway. Amplification and/or mutation of *PIK3CA*, the gene encoding the p110α catalytic subunit, occurs in 20–40 % of breast cancers, leading to an increase in activity of the enzyme. Moreover, expression of mutant *PIK3CA* in the mouse mammary gland induces heterogeneous mammary tumors with features resembling human breast cancer [5, 6]. Further mechanisms of PI3K pathway hyperactivation include phosphatase and tensin homolog (PTEN) loss of function (30 % of breast cancers), activation of receptor tyrosine kinases (RTK), and the amplification or mutation of *AKT* [7].

Not surprisingly, members of the PI3K pathway are attractive therapeutic targets in oncology. Although a broad range of PI3K inhibitors are currently in clinical trials, the responses to these compounds as single agents are less robust than expected. Isoform-selective PI3K inhibitors are highly specific and thus can be used at higher concentrations than pan-PI3K inhibitors, resulting in a more robust target inhibition, while limiting side-effect complication [8]. However, the combination of isoform-selective PI3K inhibitors with additional agents may require the use of lower concentrations to avoid potential toxicities.

Screening of a panel of cancer cell lines has revealed the hypersensitivity of cells with *PIK3CA* mutations to the α-specific inhibitor BYL719 [9]. Early clinical trials evaluating BYL719 were restricted to patients with *PIK3CA*-mutated solid tumors and showed promising clinical activity with prolonged disease stabilization and tumor shrinkage [10]. Anticipating potential mechanisms of resistance to PI3K α-specific inhibitors such as BYL719 is crucial in order to rationally stratify patients for such therapy and design efficacious combinations.

To identify potential molecular mechanisms of resistance, we developed BYL719-resistant breast cancer cell lines and used unbiased global phosphoproteomic approaches to assess changes in signaling molecules and pathways as well as functional assays. We found that insulin growth factor receptor (IGF1R)/p110β-evoked AKT/mammalian target of rapamycin (mTOR)/S6K activation stifles the effects of BYL719. Combination of p110α inhibition with inhibitors of IGF1R or p110β circumvents BYL719 resistance. Thus, we have discovered an important mechanism of resistance to PI3K α-specific inhibition and propose that the combination of the described inhibitors may be more efficacious in treating human breast tumors than any of the single agents.

## Materials and methods

### Cell lines

Human cell lines T47D and MCF7 were obtained from the American Type Culture Collection (Manassas, VA, USA), were authenticated by single nucleotide polymorphism fingerprinting, and were generally used within 20 passages. The cell lines were maintained in RPMI medium supplemented with 10 % fetal bovine serum, 10 μg/ml human insulin solution, 100 IU/ml penicillin, and 100 μg/ml streptomycin. All lines were maintained at 37 °C with 5 % CO_2_. To develop resistant models, parental cell lines were chronically treated with IC90: (90 % inhibitory concentrations) of BYL719 (2 μM for T47D and 5 μM for MCF7) over a period of 5–6 months until resistance occurred. Fresh media were provided every 2 days.

### Antibodies and reagents

Antibodies used in this study were anti-pAKT Ser473, anti-AKT, anti-pS6 Ser235/236, anti-S6, anti-pPRAS40 Thr246, anti-pERK1/2 Thr202/Tyr204, anti-ERK1/2, anti-p110α, anti-p110β, anti-PTEN, anti-IRS1 (Insulin receptor substrate), anti-poly (ADP-ribose) polymerase (anti-PARP), and anti-cleaved PARP from Cell Signaling Technology (Danvers, MA, USA), anti-phosphotyrosine 4G10 and anti-p85 from Millipore (Billerica MA, USA) anti-pIGF1R/IR Tyr1162/1163 from Biosource (ThermoFisher Scientific, Waltham MA, USA) and anti-IRS2 from Novus Biological (Littleton CO, USA). BYL719, AEW541, RAD001, and MEK162 were provided by Novartis Pharma AG (Basil, Switzerland). MK2206, AZD6482, GSK2334470, CAL101, and BMS354825 were purchased from Selleckchem (Houston TX, USA). All compounds used in vitro were dissolved in dimethyl sulfoxide (DMSO).

### Sample preparation and phosphotyrosine immunoprecipitation for liquid chromatography–mass spectrometry

Cells were harvested in lysis buffer (200 mM ammonium bicarbonate, pH 7.5, 8 M urea) supplemented with the PhosStop phosphatase inhibitor cocktail Roche Diagnostics (Rotkreuz, Switzerland), reduced and alkylated, and then digested with Trypsin/LysC Promega (Madison WI, USA) after dilution to 2 M urea. The peptides were acidified to 1 % TFA (trifluoroacetic acid) and desalted on SepPak C18 cartridges. Lyophilized peptides were dissolved in immunoprecipitation buffer (50 mM ammonium bicarbonate, pH 7.4, 150 mM NaCl, 1 % (w/v) octyl-β-d-glucopyranoside, and Roche protease and phosphatase inhibitors (Complete and PhosStop, Roche Diagnostics, Rotkreuz, Switzerland) and incubated with 200 μl of anti-phosphotyrosine antibodies (PY99; SantaCruz, Dallas TX, USA) for 16 hours at 4 °C. After elution of the beads with 0.1 % TFA, peptides were desalted on Poros R3 and further purified on TiO_2_ microcolumns.

### Analysis by liquid chromatography/tandem mass spectrometry

The purified phosphopeptides were resuspended in 10 % formic acid and injected onto a 15 cm × 75 μm ProteoPep 2 PicoFrit column (New Objectives, Woburn MA, USA) connected to an LTQ-OrbiTrap Elite mass spectrometer Thermo (ThermoFisher Scientific, Waltham MA, USA). Buffer A consisted of H_2_O with 0.1 % formic acid and Buffer B of 100 % acetonitrile with 0.1 % formic acid. Peptides were separated using a two-step gradient, from 0 % B to 20 % B in 90 minutes and from 20 % B to 50 % B in 50 minutes. Data acquisition used a “Top 15 method”, where every full mass spectrometry (MS) scan was followed by 15 data-dependent scans on the 15 most intense ions from the parent scan. Full scans were performed in the OrbiTrap at 120,000 resolution with target values of 1E6 ions and 500 milliseconds injection time, while MS/MS scans were performed in the ion trap with 1E4 ions and 200 milliseconds injection time. Database searches were carried out with Mascot Server (Matrix Science, London UK) using the human Uniprot database (release 20130429, www.uniprot.org). Mass tolerances were set at 10 ppm for the precursor and at 0.8 Da for the fragment ions. In the case of ambiguous assignments, spectra were manually interpreted for confirmation of identity and localization of the phosphorylation site using Scaffold (version 4.3; Proteome Software, Portland OR, USA). Label-free quantification was performed on duplicate liquid chromatography (LC)–MS runs for each sample using Progenesis LC-MS (Nonlinear Dynamics Software, Newcastle-upon-Tyne, UK). Normalized peptide intensities were added together for each unique phosphorylated peptide with Mascot scores exceeding 20, and used to calculate the log_2_ ratios between samples for each unique phosphopeptide.

### Immunoprecipitation and western blotting

Total proteins were extracted with 50 mM Tris–HCl, pH 7.6, 150 mM NaCl, 1 % NP40, and Roche phosphatase and protease inhibitor cocktail. Immunoprecipitation was performed overnight at 4 °C on total protein lysates with the indicated antibodies according to the supplier’s recommendations. Protein G sepharose beads were then added for 1 hour at 4 °C. Proteins were eluted with 50 μl loading buffer (45 μl LDS sample buffer + 5 μl Reducing Agent; Novex, ThermoFisher Scientific, Waltham MA, USA,) and then boiled at 95 °C for 5 minutes. Immunoprecipitates or 50 μg of proteins for a whole cell lysate were loaded onto 4–12 % SDS-PAGE gels using the NuPAGE system from Invitrogen (ThermoFisher Scientific, Waltham MA, USA) (30 mA for 10 minutes and 50 mA for 1 hour 15 minutes) and then transferred onto Invitrolon PVDF membranes in the Biorad Blotter system (Hercules CA, USA), using blotting buffer containing 25 mM Tris-Base, 192 mM glycine, and 5 % methanol (100 V for 30 minutes). Detection was by chemiluminescence using the Western Bright ECL detection kit Advansta (Menlo Park CA, USA). Immunoblots are representative of a minimum of three independent experiments. An equal amount of protein was loaded onto each gel and the immunoblots shown in the same figure were developed simultaneously with the same exposure time.

### Cell number count

Standard cell growth was performed in 2 % fetal calf serum medium with appropriate concentrations of the indicated inhibitors using 24-well plates (50,000 cells/well) and measured by sulforhodamide B staining according to the manufacturer’s instructions (Sigma Aldrich, St. Louis MO, USA). Dose-response experiments were evaluated at day 3 and time course experiments at days 3, 6, and 9. In the time course experiments, cells were plated and treatments with DMSO vehicle (VHC) or the indicated inhibitors started 6 hours later. VHC-treated resistant cell lines do not have BYL719 in the media all through the experiment.

### Apoptosis assay

A total of 70,000 cells/well were seeded in six-well plates. After overnight incubation, media were aspirated and replaced with media with the indicated drugs. After 72 hours, the media were collected and cells were harvested, washed twice with cold phosphate-buffered saline (PBS), and resuspended in Annexin binding buffer. Cells were stained with propidium iodide (PI) and Annexin V according to the manufacturer’s protocol (BD Biosciences, San Jose, CA, USA).

### Xenograft mouse experiment

Female Balb-c nude mice were used in compliance with the Swiss laws on animal welfare and the animal protocols were approved by the Swiss Cantonal veterinary Office of Basel. MCF7 cells (5 × 10^6^ cells) were suspended in a 50 μl mixture of Matrigel (BD Biosciences, San Jose CA, USA) and PBS (1/1), and were injected subcutaneously into the right flank of female Balb-c nude mice 6–8 weeks old. Mice were implanted with estrogen pellets on the day of cell injection. Tumor-bearing mice were randomized into six groups of five or six mice based on tumor volume prior to initiation of treatment, which started when the average tumor volume was between 150 and 200 mm^3^. BYL719, AEW541, and GSK2636771 were given orally daily. BYL719 and GSK2636771 were dissolved in CMC/Tween and AEW541 in NMP/PEG300 (1/9). VHC-treated mice received a combination of CMC/Tween and NMP/PEG300. Tumors were measured every 5 days and tumor volumes calculated by the formula:

$$ \mathrm{Tumor}\ \mathrm{volume} = \left({\left(\mathrm{smaller}\ \mathrm{diameter}\right)}^2 \times \left(\mathrm{larger}\ \mathrm{diameter}\right)\right)\ /\ 2. $$

### Statistical analysis

For dose-response experiments, one-way analysis of variance analysis was performed using GraphPad Prism (Graphpad, La Jolla CA, USA) 6.0 software to determine GI50 (50 % growth inhibition) growth inhibition values. For all analyses, reported values represent the means ± standard error of the mean (SEM) of at least three independent experiments. Data were tested with Student’s *t* test and *P* <0.05 was considered statistically significant.

## Results

### Sustained mTOR activity leads to BYL719 resistance in breast cancer cells harboring *PIK3CA* mutation

**Fig. 1:**
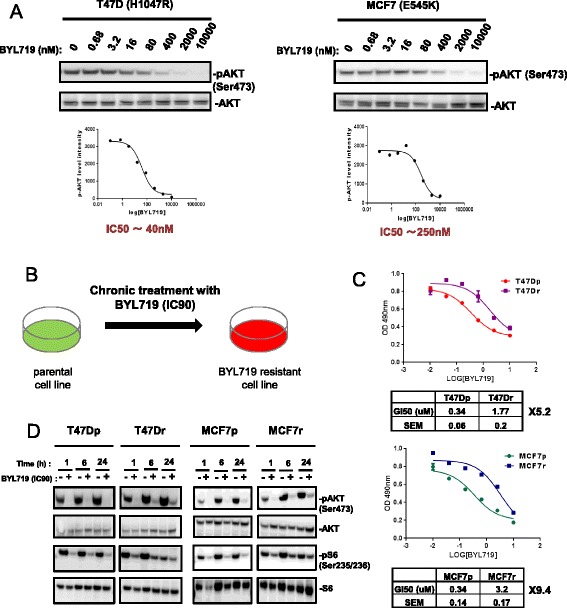
Generation of BYL719-resistant breast cancer cell lines. **a** Immunoblots of lysates from T47D and MCF7 cells treated for 1 hour with increasing concentrations of BYL719. Immunoblot quantification used ImageJ software (developed at the U. S. National Institutes of Health and available on the Internet at http://rsb.info.nih.gov/nih-image/). **b** Schematic of the generation of BYL719-resistant breast cancer cell lines. Parental cells were treated chronically with respective IC90 (90 % inhibitory concentrations) of BYL719 for a period of 5–6 months. **c** Curves showing BYL719 dose-responses of parental and resistant lines after 3 days of treatment. Cell number was evaluated using the sulforhodamide B assay. GI50 (50 % growth inhibition) values were calculated using GraphPad Prism 6 software. Data are mean ± SEM (*n* >3). **d** Immunoblots of lysates from parental and resistant cells treated with respective IC90 (90 % inhibitory concentrations) of BYL719 as indicated. *MCF7p* MCF7-parental, *MCF7r* MCF7-resistant, *SEM* standard error of the mean, *T47Dp* T47D-parental, *T47Dr* T47D-resistant

BYL719 was initially evaluated in clinical trials for luminal breast cancer with *PIK3CA* mutations. Thus, to investigate the mechanisms of resistance to BYL719, we selected the BYL719-sensitive luminal human breast cancer cell lines T47D and MCF7 harboring the H1047R and E545K *PIK3CA* hotspot mutations, respectively. We first calculated IC50 (50 % inhibitory concentration) values for BYL719 using pAKT Ser473 immunoblotting as a readout of p110α inhibition (Fig. 1a). We next developed BYL719-resistant cell lines by chronically treating parental T47D and MCF7 cells with BYL719 at IC90 (2 μM for T47D; 5 μM for MCF7) (Fig. 1b). BYL719 blocked T47D and MCF7 cells in the G1 phase, causing proliferation arrest for 5–6 weeks (data not shown). Thereafter, inhibition of PI3K by chronic BYL719 treatment was overcome and cells started to grow. Four months later, cells became resistant to the compound, with a change in GI50 values relative to parental lines of 5.2-fold for T47D-resistant (T47Dr) cells and 9.4-fold for MCF7-resistant (MCF7r) cells (Fig. 1c). GI50 values calculated in our experiments correlate with values reported by Vora et al. [11]. IC50 values for BYL719 measured by the AKT phosphorylation level are different between these two cell lines, but the GI50 values for BYL719-mediated inhibition of cell proliferation are identical. This suggests a differential requirement between these two cell lines for AKT signaling to drive cell proliferation (Fig. 1a, c). Interestingly, resistant cells cultured for 2 weeks in the absence of BYL719 completely lost their resistance, showing that the mechanism of resistance was reversible (see Additional file 1A).

We next investigated the effects of BYL719 on components of the PI3K pathway in resistant and parental cells. Phosphorylation of AKT at Ser473 was reduced to a similar extent in parental and resistant cells (Fig. 1d). In some experiments we did see a slightly higher level of AKT phosphorylation in the resistant cells compared with the parental cells after 6–24 hours of treatment (as in Fig. 1d), but this was not consistently observed, leading us to conclude that the pathway responses at the level of Akt were similar between parental and resistant cells. In contrast, whereas a 70 % decrease in S6 phosphorylation at Ser235/236 was observed in parental cell lines, a smaller 20 % decrease occurred in resistant cells (Fig. 1d; see also Additional file 1B) suggesting that sustained mTOR activity leads to BYL719 resistance in *PIK3CA* mutant breast cancer cells.

### Phosphoproteomics revealed increases in IGF1R, IRS, and p85 PI3K tyrosine phosphorylation in resistant cell lines

**Fig. 2:**
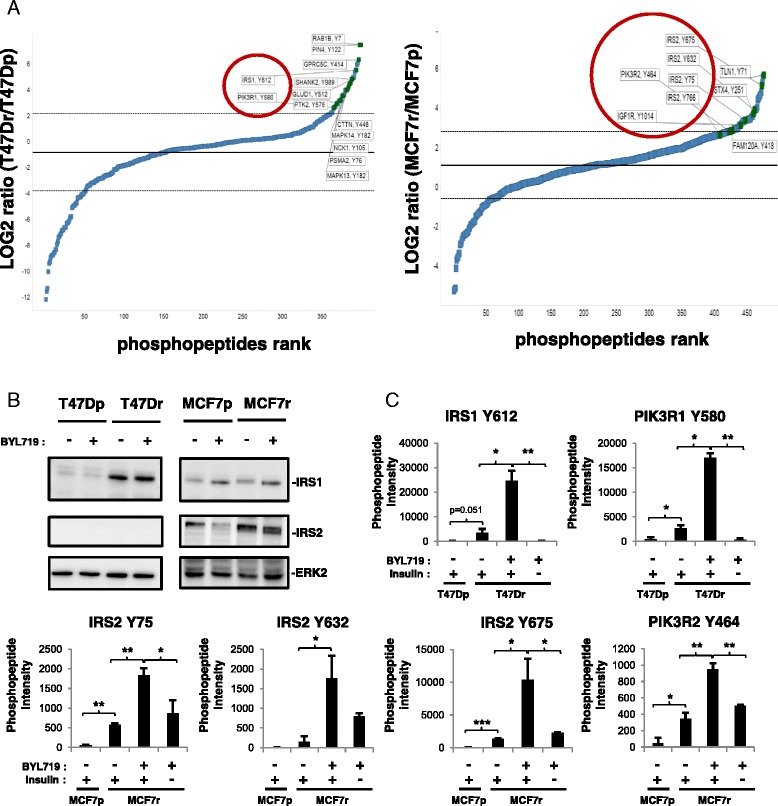
BYL719 resistance correlates with increases in IGF1R, IRS (insulin receptor substrate), and p85 PI3K tyrosine phosphorylation. **a** Fold changes in tyrosine phosphorylation in resistant cells compared with parental cells without BYL719 treatment, expressed as the log_2_ resistant/parental ratio and displayed in rank order from low to high. Graphs were generated with TIBCO Spotfire software (TIBCO Software, Boston MA, USA). **b** Immunoblots of lysates from parental and resistant lines treated for 24 hours with 2 μM (T47D) or 5 μM (MCF7) BYL719 as indicated. **c** Bar graphs representing intensities of phosphopeptides bearing the indicated tyrosine phosphosites as identified by LC-MS/MS. T47D and MCF7 parental and resistant cells were treated for 24 hours with respective IC90 concentrations of BYL719 in the presence or absence of insulin in the media. Data are mean ± STD of technical duplicates (*n* >2, *P <0.05, **P <0.01, ***P ˂0.001). *MCF7p* MCF7-parental, *MCF7r* MCF7-resistant, *PI3K* phosphatidylinositol 3-kinase, *T47Dp* T47D-parental, *T47Dr* T47D-resistant

Unbiased tyrosine phosphoproteomics was used to identify the molecular mechanism underlying sustained mTOR activity in BYL719-resistant lines. Combined totals of 398 and 475 phosphotyrosine peptides, derived from 266 and 307 proteins, were identified in parental and resistant T47D and MCF7 cells, respectively (see Additional files 2 and 3). Increased phosphorylation on tyrosine phosphosites of IGF1R, IRS1, IRS2, and p85 PI3K were found in T47Dr and MCF7r cells relative to the parental lines (Fig. 2a). Immunoblotting analysis demonstrated that the increases in IRS (Insulin receptor substrate) phosphorylation correlated with increases in IRS1 and IRS2 protein expression in T47Dr and MCF7r cells, respectively (Fig. 2b). Treatment of T47Dr and MCF7r cells with BYL719 for 24 hours increased IRS1 and IRS2 tyrosine phosphorylation but had no effect on IRS1 and IRS2 protein expression levels (Fig. 2b, c). Moreover, BYL719 treatment also induced an increase in p85 PI3K tyrosine phosphorylation in the resistant lines (Fig. 2c). Interestingly, insulin withdrawal abrogated IRS1, IRS2, or p85 tyrosine phosphorylation observed in resistant cells upon BYL719 treatment, suggesting that the insulin/IGF1R/IRS pathway contributes to BYL719 resistance. Taken together, our results suggest an important effect of the IGF1R/IRS tandem on resistance to BYL719.

### IGF1R mediates resistance to BYL719

**Fig. 3:**
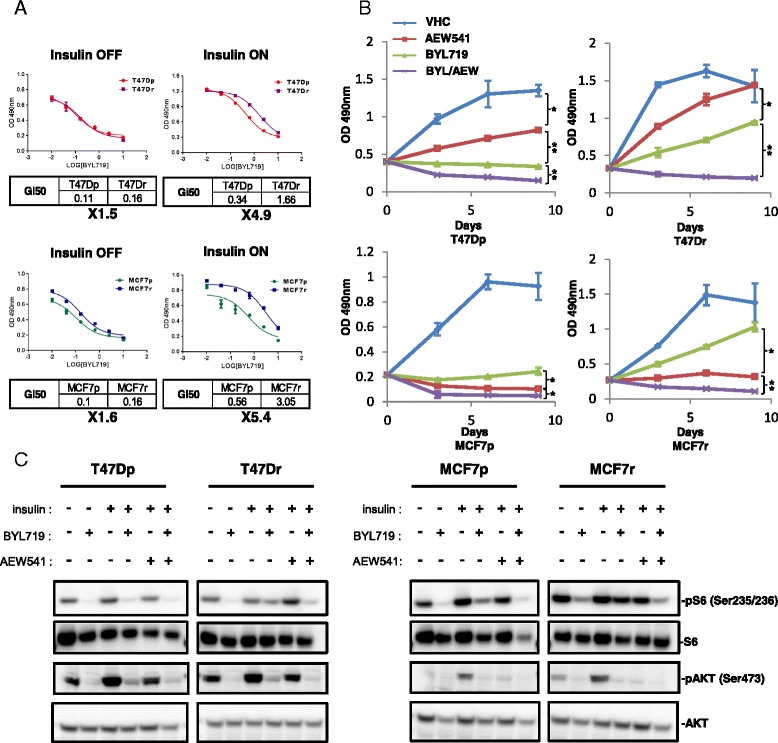
BYL719 resistance of breast cancer cell lines is dependent on IGF1R activity. **a** BYL719 dose-responses of parental and resistant lines after 3 days of treatment in the presence or absence of insulin in the culture media. Cell numbers were evaluated using the sulforhodamide B assay. GI50 (50 % growth inhibition) values were calculated using GraphPad Prism 6 software. Data are mean ± SEM (*n* = 3). **b** Proliferation of parental and resistant cells treated with vehicle (*VHC*), AEW541 (1 μM), BYL719 (IC90), or a combination of BYL719 and AEW541 for 9 days. Cell numbers were evaluated using the sulforhodamide B assay. Data are mean ± SEM (*n* >3, *P <0.01, **P <0.001). **c** Immunoblots of lysates from parental and resistant cells treated for 24 hours as indicated. BYL719 (IC90) and AEW541 (1 μM). *MCF7p* MCF7-parental, *MCF7r* MCF7-resistant, *T47Dp* T47D-parental, *T47Dr* T47D-resistant

The phosphoproteomic data suggested the insulin/IGF1R/IRS axis as a driver of resistance to BYL719. Interestingly, removal of insulin from the culture media restored the sensitivity of resistant cells to BYL719 (Fig. 3a). This observation correlates with the decrease of tyrosine phosphorylation of IRS1, IRS2 and p85 in resistant cell lines upon insulin withdrawal (Fig. 2c). We then compared the effects of BYL719 and the IGF1R inhibitor AEW541, alone or in combination, on parental and resistant cell lines. Combined inhibition of p110α and IGF1R reversed BYL719 resistance (Fig. 3b) and increased cell death of resistant cells, as shown by an increase in PARP cleavage and apoptosis assay (PI and Annexin V) (see Additional file 4A, B). Although the combination BYL719/AEW541 resulted in significant growth inhibition, MCF7 cells appear to be more dependent on the IGF1R pathway as suggested by the sensitivity of MCF7 cells to AEW541 when used as a single agent (Fig. 3b). This observation is in line with previous studies [12] showing that the *PIK3CA* E545K mutant (as in MCF7) requires IRS tyrosine phosphorylation to be fully activated and drive the downstream pathway. Combination of BYL719 with either insulin withdrawal or AEW541 treatment resulted in decreases in AKT Ser473 and S6 Ser235/236 phosphorylation (Fig. 3c). Moreover, either insulin withdrawal in the absence of BYL719 or AEW541 single-agent treatment decreased AKT phosphorylation in MCF7 cells but not in T47D cells, confirming the dependency of MCF7 cells on IGF1R-driven IRS tyrosine phosphorylation. These data show that IGF1R-evoked mTOR activation is a key driver of resistance to BYL719 in these models.

### Activation of IGF1R/p110β/AKT/mTOR produces resistance to BYL719

**Fig. 4:**
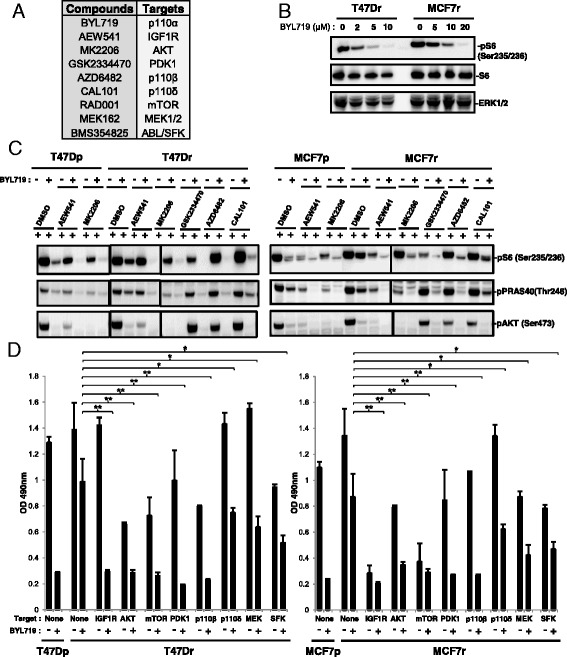
Activation of IGF1R/p110β/AKT/mTOR produces resistance to BYL719. **a** Compounds used and their respective targets. **b** Immunoblots of lysates from parental and resistant cells treated for 24 hours as indicated. **c** Immunoblots of lysates from parental and resistant cells treated for 24 hours with the respective IC90 concentrations of BYL719 and/or 1 μM of the indicated compounds. **d** Proliferation of parental and resistant cells treated with the respective IC90 concentrations of BYL719 in combination with 1 μM of the indicated inhibitors. Cell numbers were evaluated using the sulforhodamide B assay. Data are mean ± SEM (*n* >2, *P <0.05, **P <0.01). *DMSO* dimethyl sulfoxide, *IGF1R* insulin growth factor receptor, *MCF7p* MCF7-parental, *MCF7r* MCF7-resistant, *mTOR* mechanistic target of rapamycin, *SFK* Src family kinases, *T47Dp* T47D-parental, *T47Dr* T47D-resistant

To address the mechanism of resistance to BYL719, we used inhibitors of molecules and pathways known to result in resistance to PI3K inhibition (Fig. 4a). Concentrations of BYL719 that have been shown to inhibit the other p110 isoforms [9] also downregulated mTOR activity (Fig. 4b). PDK1-specific, AKT-specific, and p110β-specific inhibitors in combination with BYL719 abrogated S6 and PRAS40 phosphorylation, whereas the p110δ inhibitor did not (Fig. 4c; see also Additional file 5). These data suggest that an IGF1R/p110β/AKT/mTOR pathway increases resistance to BYL719. Inhibition of IGF1R, p110β, PDK1, AKT, or mTOR in combination with BYL719 reduced T47Dr and MCF7r cell growth, whereas inhibition of p110δ, MEK, or Src family kinases (SFK) showed only a partial effect (Fig. 4d). Accordingly, MEK and SFK inhibition in combination with BYL719 did not decrease S6 or PRAS40 phosphorylation (see Additional file 6). Taken together, our data highlight the activation of IGF1R/p110β/AKT/mTOR as a mechanism of resistance to BYL719 in *PIK3CA*-mutated breast cancer cells.

### Phosphorylated IRS and p85 recruit p110β in BYL719-resistant lines

**Fig. 5:**
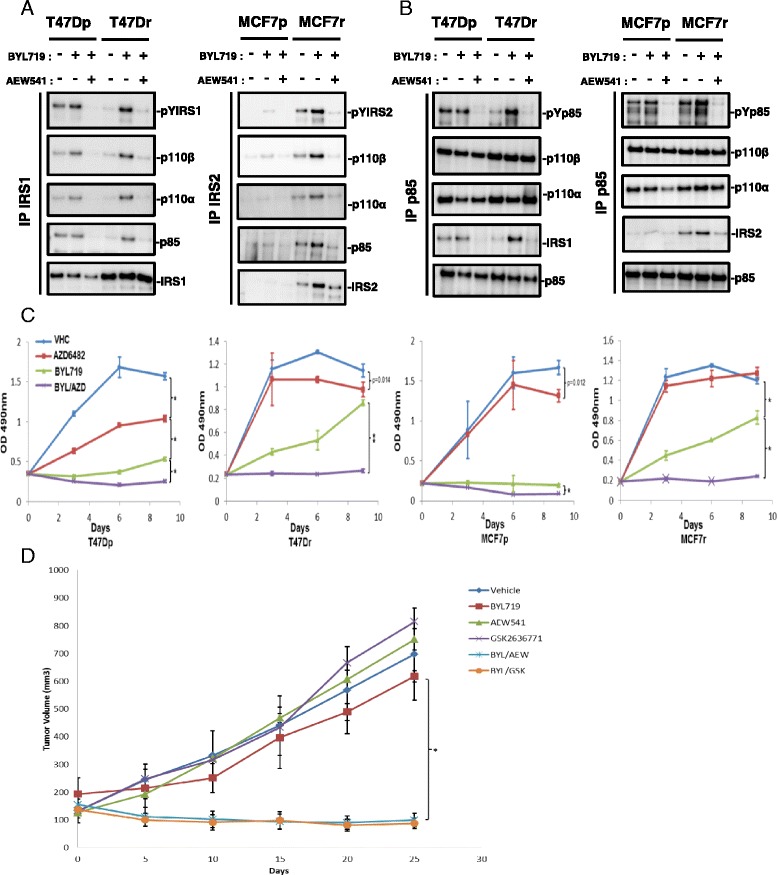
p110β is activated in an IGF1R/IRS (insulin receptor substrate)-dependent manner and promotes resistance to BYL719. **a** Immunoblots of coimmunoprecipitated p110β, p110α, or p85 PI3K from lysates of parental and resistant cells treated for 24 hours with or without IC90 concentrations of BYL719 combined with or without 1 μM of AEW541. **b** Immunoblots of immunoprecipitated p85 PI3K antibody from lysates of parental and resistant cells treated for 24 hours with IC90 concentrations of BYL719 combined with or without 1 μM AEW541. **c** Proliferation of parental and resistant cells treated with vehicle (*VHC*), AZD6482 (1 μM), BYL719 (IC90), or a combination of BYL719 and AZD6482 for 9 days. Cell numbers were evaluated using the sulforhodamide B assay. Data are mean ± SEM (*n* >3, *P <0.01, **P <0.001). **d** Growth curves of MCF7 tumors from Balb-c nude mice treated with VHC, 25 mg/kg BYL719 (p110α inhibitor) daily, 75 mg/kg AEW541 (IGF1R inhibitor) daily, 100 mg/kg GSK2636771 (p110β inhibitor) daily, alone or in combination as indicated. Data are mean ± SEM (*n* >5, *P <0.001). *IP* immunoprecipitation, *MCF7p* MCF7-parental, *MCF7r* MCF7-resistant, *T47Dp* T47D-parental, *T47Dr* T47D-resistant

We next assessed the mechanism of p110β activation in BYL719-resistant cell lines. Because we had found that IGF1R/IRS mediates resistance to BYL719 and that p85 and IRS tyrosine phosphorylation are increased in an insulin-dependent manner in resistant cells (Fig. 2c), we hypothesized that an IGF1R/IRS/p85 complex, active through tyrosine phosphorylation of IRS and p85, recruits and activates p110β. To test this possibility, we treated parental and resistant cells with BYL719 alone or in combination with AEW541 and performed IRS1 (T47D lysates), IRS2 (MCF7 lysates), or p85 immunoprecipitation, followed by anti-phosphotyrosine immunoblotting. This confirmed the increased tyrosine phosphorylation of IRS and p85 previously observed in resistant cells upon BYL719 treatment (Figs. 2c and 5a, b). Interestingly, we observed coimmunoprecipitation of p110β/p85 with IRS when IRS and p85 were tyrosine phosphorylated. Conversely, the IRS/p110β/p85 complex was disrupted upon IGF1R inhibition (Fig. 5a, b).

Loss of PTEN expression was demonstrated recently in metastases from a patient showing relapsed response to BYL719 therapy [13], and PTEN null breast cancers have been shown to be driven by p110β and not by p110α [14, 15]. We tested whether the p110β dependency of BYL719-resistant cells is due to PTEN loss but found no difference in PTEN expression between resistant and parental lines (see Additional file 7). Our results indicate that IRS and p85 tyrosine phosphorylation by IGF1R are necessary for p110β recruitment and activation in BYL719-resistant lines.

### p110β promotes resistance to BYL719 in an IGF1R/IRS-dependent manner

We next tested the effect of p110β inhibition alone or in combination with BYL719 on the survival of parental and resistant T47D and MCF7 cells. Notably, combined p110α and p110β inhibition decreased cell numbers in both parental and resistant cell lines (Fig. 5c). Inhibition of p110β had no effect on T47Dr, MCF7-parental (MCF7p), or MCF7r cell lines (Fig. 5c). This inhibition partially decreased the number of T47D-parental (T47Dp) cells (Fig. 5c), which is in agreement with the observed coimmunoprecipitation of p110β with IRS1 only in T47Dp cells (Fig. 5a). We conclude that p110β inhibition circumvents the resistance of *PIK3CA*-mutated breast cancer cells to BYL719. We next confirmed our observations in an in vivo xenograft mouse experiment with MCF7 cells. A combination of BYL719 with IGF1R (AEW541) or p110β inhibitors (GSK2636771 was used as a p110β inhibitor instead of AZD6482 to ensure better tolerability in combination) strongly decreased tumor growth whereas either agent alone did not (Fig. 5d). Our results show that IGF1R-evoked p110β activation produces resistance to BYL719 in vivo.

## Discussion

Mechanism-based inhibitors that target cancer dependencies have been shown to be effective and well tolerated in several clinical trials but, unfortunately, most did not provide long-lasting therapy. Emergent resistance can result in relapse of disease within a few months [16, 17]. Anticipation of potential molecular mechanisms of resistance to a given targeted therapy is therefore crucial for the selection of patients who may benefit from the treatment and, when necessary, for the rational design of combination therapies [18].

The PI3K pathway is one of the most attractive therapeutic targets and numerous inhibitors are being evaluated in clinical trials [8]. Pan-PI3K inhibitors are often used at a dose that does not completely block PI3K activity, which may not result in acceptable efficacy. Isoform-specific inhibitors are considered to have a better therapeutic index than dual PI3K/mTOR or pan-PI3K inhibitors currently used in the clinic [10]. Accordingly, α-specific PI3K inhibitors like BYL719 have shown promising preclinical and clinical results with cancers harboring mutated and/or amplified *PIK3CA* [9, 10]. Unfortunately, clinical responses to α-specific PI3K inhibitors as single agents have not met expectations because of intrinsic and/or developed resistance. Therefore, in addition to aiding patient stratification and the identification of appropriate biomarkers, anticipating mechanisms of resistance to PI3K inhibition is essential for the rational design of combination therapies.

**Fig. 6:**
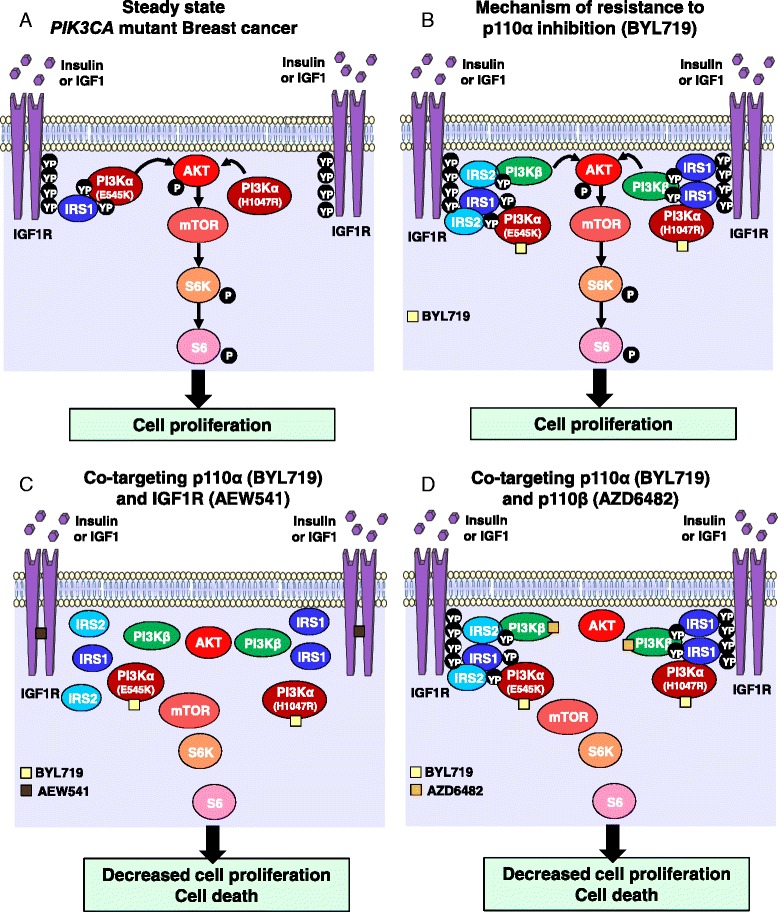
Schematic model of IGF1R-evoked and p110β-evoked resistance to p110α inhibition in *PIK3CA* mutant breast cancer cells. Schematics illustrating **a** the steady state of the PI3K pathway in *PIK3CA* mutant breast cancer, and **b** the identified feedback loop triggered by inhibition of p110α (BYL719) and offset by **c** IGF1R (AEW541) or **d** p110β (AZD6482) inhibition at the cellular level. *IGF1R* insulin growth factor receptor, *mTOR* mechanistic target of rapamycin, *PI3K* phosphatidylinositol 3-kinase

In the present study, we have discovered that IGF1R-evoked p110β activation compensates for selective p110α inhibition. Evidence is provided by our findings that inhibition of members of the IGF1R/p110β/AKT/mTOR pathway improves the efficacy of p110α inhibition. These data provide a rationale for combining α-specific PI3K inhibitors with inhibitors of the IGF1R/p110β/AKT/mTOR axis (Fig. 6).

Various mechanisms of resistance to dual PI3K/mTOR inhibitors have been reported, such as *MYC* amplification [19, 20], MEK/ERK/RSK pathway activation [21], or BCL2, epidermal growth factor, and IGF1R [22]. Recently, mTORC [23] or CDK4/6 [11] inhibition has been shown to sensitize *PIK3CA* mutant breast cancers to α-specific PI3K inhibitors. PTEN loss has also been identified as the basis of resistance to BYL719 in a small set of patients with breast cancer that progressed further after an initial beneficial response to BYL719 [24]. Recently, measurement of PIP3 levels revealed that the efficiency of p110α inhibition was compensated by the activation of p110β in an early adaptive response which mitigates the anti-tumor efficacy of BYL719 [25]. Here, we demonstrate the importance of p110β in *PIK3CA*-mutated breast cancer cell lines. Although in PTEN null breast cancers, p110β activity was induced by G protein coupled receptor (GPCR) [14, 15], in our models of resistance to BYL719, p110β is activated in an IGF1R/IRS-dependent manner and contributes to AKT and mTOR activation, and ultimately to resistance to p110α inhibition. Since blocking IGF1R was sufficient to abrogate the resistance, it is likely that GPCRs are not involved in p110β activation in our system.

Our data suggest that inhibition of various components of the IGFR/PI3K/mTOR pathway is more efficacious than inhibition of p110α alone. Vertical inhibition of members of the same pathway was shown previously to efficiently abrogate mitogen-activated protein kinase (MAPK) signaling [26–28], and this scenario has also been suggested recently for the PI3K pathway [11, 29]. We find that inhibition of AKT reverses resistance to BYL719. This observation is surprising as resistant cells showed complete reduction of AKT phosphorylation upon treatment with BYL719, suggesting that AKT was inactive. Equally surprising was the observation that PRAS40, a direct substrate of AKT, was phosphorylated on Thr246 but AKT was not. Further analysis of AKT phosphorylation sites by LC-MS/MS may resolve this paradox.

BYL719-resistant breast cancer cells showed a high dependency on the insulin pathway; removal of insulin from the medium was sufficient to abrogate resistance. Hyperglycemia is the most frequently observed side effect in patients treated with PI3K inhibitors and is an indication of effective PI3K inhibition. Our data highlight the importance of insulin levels for the response to BYL719 and its potential implication in intrinsic or developed resistance. Thus, a combination of PI3K and IGF1R inhibitors would be an attractive therapeutic strategy to counteract insulin-dependent resistance. IGF1R inhibition blocks both the PI3K and the MAPK pathways when RAF and RAS are not mutated, suggesting that combination of p110α and IGF1R inhibitors may be more efficacious. Given that hyperglycemia has been reported for both PI3K and IGF1R inhibitors, combination of the two may result in increased side effects. A phase I/II clinical trial has started recently for patients with ovarian cancer or breast cancer to evaluate the therapeutic benefit of combining BYL719 with the anti-IGF1R antibody AMG479 (ClinicalTrials.gov NCT01708161). The results should indicate whether inhibition of IGF1R improves BYL719 efficacy and is well tolerated by patients. Testing a combination of continuous p110α inhibition and intermittent IGF1R blockade is also warranted. Alternatively, continuous treatment with BYL719 and either continuous p110β inhibition or intermittent pan-PI3K inhibition may reduce potential side effects.

In the era of precision medicine, the next challenge will be to identify appropriate biomarkers that will indicate the right combination for the right patient. As S6 phosphorylation and Rb phosphorylation have been used recently to stratify patients who could benefit from combination of BYL719 with mTOR or CDK4/6 inhibitors, our data suggest that the level of expression and the tyrosine phosphorylation of IRS1 and IRS2 would be an excellent biomarker for the combination of p110α/IGF1R or p110α/p110β inhibitors.

## Conclusions

Our study demonstrates that p110β can produce resistance to α-specific PI3K inhibition in an IGF1R/p85/IRS-dependent manner in *PIK3CA*-mutant breast cancer. These data provide a rationale for combining p110α inhibition with IGF1R or p110β in patients with breast tumors harboring *PIK3CA* mutations.

## Additional files

Additional file 1: is a figure showing BYL719 resistance in *PIK3CA* mutant breast cancer cells. **A** BYL719 resistance is reversible. BYL719 dose-response of parental and resistant lines (on or off BYL719 for 2 weeks) after 3 days of treatment. Cell numbers were evaluated using the sulforhodamide B assay. GI50 values were calculated using GraphPad Prism 6 software. Data are mean ± SEM (*n* >2). **B** pS6/S6 ratios in cells treated with DMSO or BYL719 (IC90) for 24 hours. Data are mean of pS6/S6 ratio ± SEM (*n* >3, **P* <0.01). Immunoblots from three independent experiments have been quantified using the ImageJ software. (PDF 117 kb)
Additional file 2: is a table presenting phosphopeptide intensity of T47Dp with insulin but without BYL719; and T47Dr with or without insulin treated for 24 hours with or without 2 μM BYL719. (PDF 258 kb)
Additional file 3: is a table presenting phosphopeptide intensity of MCF7p with insulin but without BYL719; and MCF7r with or without insulin treated for 24 hours with or without 5 μM BYL719. (PDF 308 kb)
Additional file 4: is a figure showing that a combination of BYL719/AEW541 triggers apoptosis in BYL719-resistant cell lines. **A** Anti-cleaved PARP (T47D) or anti-PARP (MCF7) immunoblots of lysates from parental and resistant cells treated for 24 hours as indicated. BYL719 (IC90), AEW541 (1 μM). **B** Parental and resistant cells were treated with BYL719 (2 μM for T47D and 5 μM for MCF7), AEW541 (1 μM), or the combination BYL719/AEW541 for 72 hours. Percentage of apoptotic (Annexin V-positive and PI-negative) and necrotic cells (Annexin V and PI-positive) are indicated. (PDF 158 kb)
Additional file 5: is a figure showing that activation of IGF1R/p110β/AKT/mTOR produces resistance to BYL719. Immunoblots of lysates from parental and resistant cells treated for 24 hours with the respective IC90 concentrations of BYL719 and/or 1 μM of the indicated compounds. (PDF 142 kb)
Additional file 6: is a figure showing that inhibition of MEK or SFK signaling pathways in combination with BYL719 does not alter mTOR-sustained activity in resistant cells. Immunoblots of lysates from parental and resistant cells treated for 24 hours with the respective IC90 concentration of BYL719 and/or 1 μM of AEW541, 1 μM MK2206, 10 nM RAD001, 500 nM MEK162, or 1 μM BMS354825. Immunoblots have been developed simultaneously with the same time of exposure. (PDF 176 kb)
Additional file 7: is a figure showing that p110β activation does not correlate with loss of PTEN. Immunoblots of lysates from parental and resistant cells treated for 24 hours as indicated. BYL719 (IC90) and AEW541 (1 μM). (PDF 140 kb)

## Abbreviations

DMSO: Dimethyl sulfoxide
GPCR: G protein coupled receptor
IGF1R: Insulin growth factor receptor
LC: Liquid chromatography
MAPK: Mitogen-activated protein kinase
MCF7p: MCF7-parental
MCF7r: MCF7-resistant
MS: Mass spectrometry
mTOR: Mechanistic target of rapamycin
PARP: Poly(ADP-ribose) polymerase
PBS: Phosphate-buffered saline
PI: Propidium iodide
PI3K: Phosphatidylinositol 3-kinase
PIP2: Phosphatidylinositol-4,5-bisphosphate
PIP3: Phosphatidylinositol-3,4,5-trisphosphate
PTEN: Phosphatase and tensin homolog
RTK: Receptor tyrosine kinases
SEM: Standard error of the mean
SFK: Src family kinases
T47Dp: T47D-parental
T47Dr: T47D-resistant
VHC: Vehicle
