# Supplementary figures and images for: Activation of IGF1R/p110β/AKT/mTOR confers resistance to α-specific PI3K inhibition
Source: Breast Cancer Res. 2016 Apr 5;18:41. doi: 10.1186/s13058-016-0697-1 (PMC4820873; doi:10.1186/s13058-016-0697-1)

# Leroy *et al.* Additional file 1

A

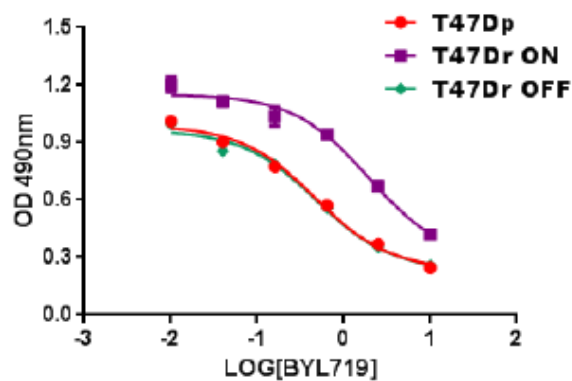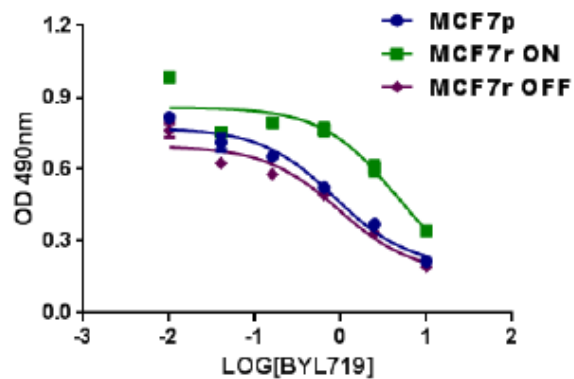

B

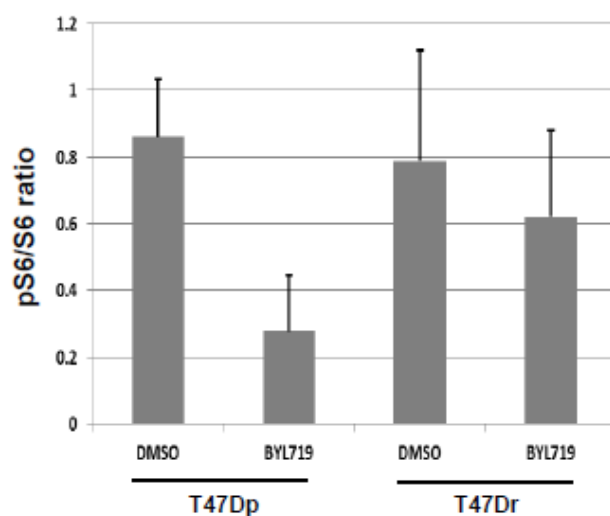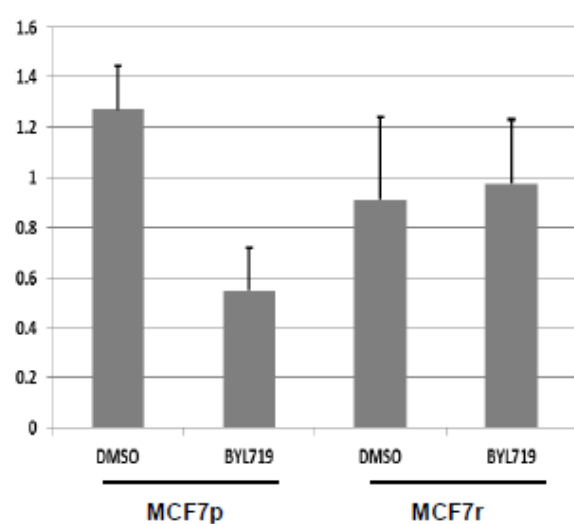

Supplement: Additional file 1: — is a figure showing BYL719 resistance in PIK3CA mutant breast cancer cells. A BYL719 resistance is reversible. BYL719 dose-response of parental and resistant lines (on or off BYL719 for 2 weeks) after 3 days of treatment. Cell numbers were evaluated using the sulforhodamide B assay. GI50 values were calculated using GraphPad Prism 6 software. Data are mean ± SEM (n >2). B pS6/S6 ratios in cells treated with DMSO or BYL719 (IC90) for 24 hours. Data are mean of pS6/S6 ratio ± SEM (n >3, *P <0.01). Immunoblots from three independent experiments have been quantified using the ImageJ software. (PDF 117 kb) [file 13058_2016_697_MOESM1_ESM.pdf]

# Leroy *et al.* Additional file 4

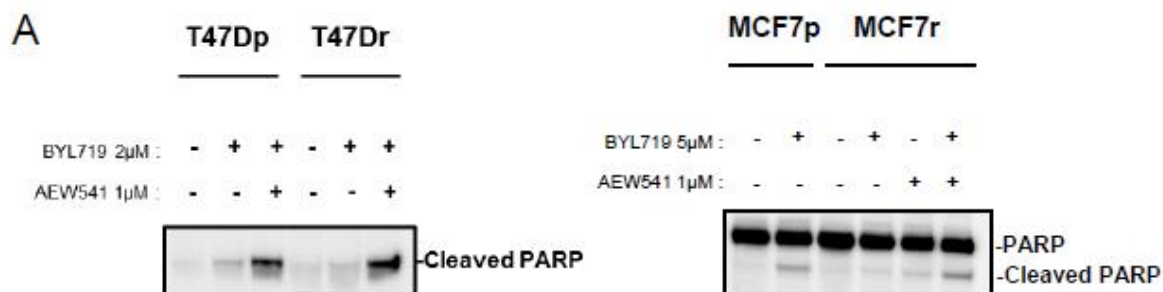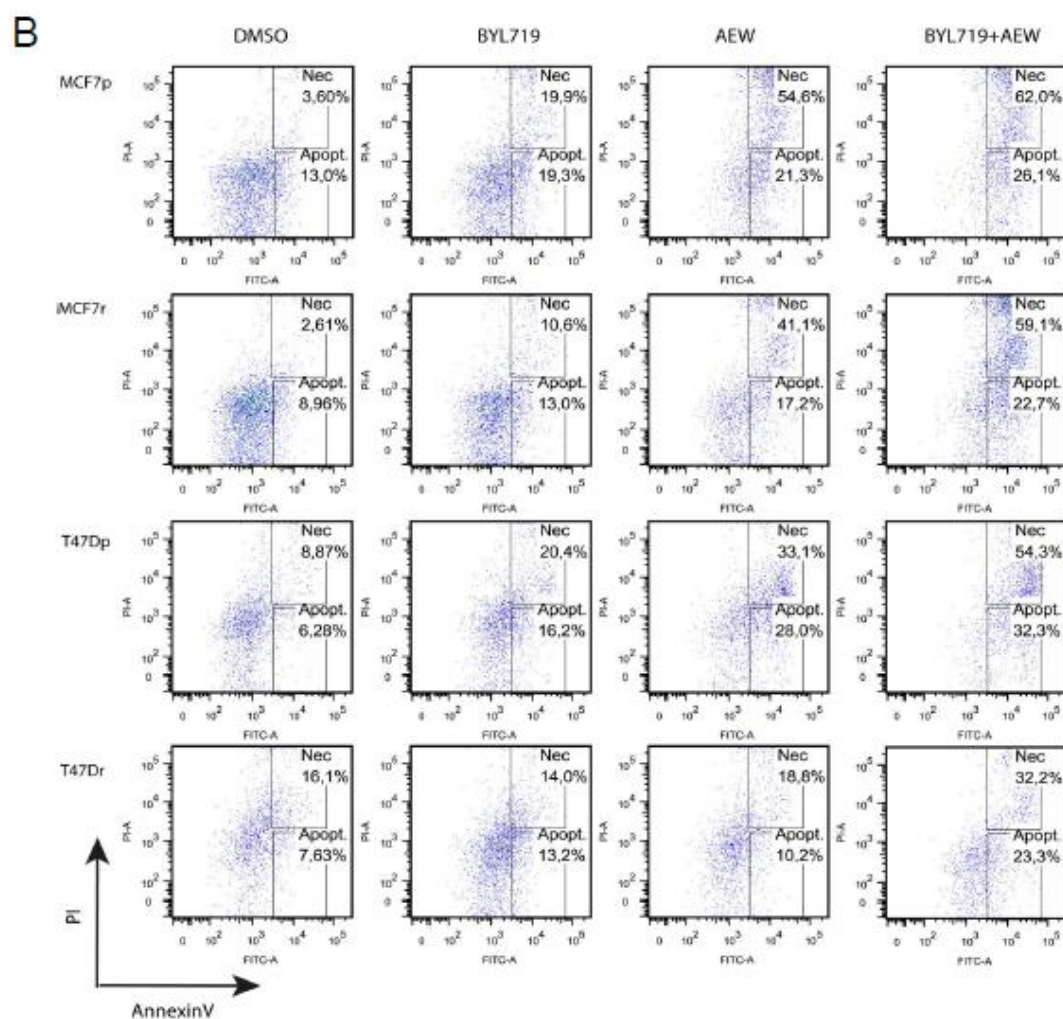

Supplement: Additional file 4: — is a figure showing that a combination of BYL719/AEW541 triggers apoptosis in BYL719-resistant cell lines. A Anti-cleaved PARP (T47D) or anti-PARP (MCF7) immunoblots of lysates from parental and resistant cells treated for 24 hours as indicated. BYL719 (IC90), AEW541 (1 μM). B Parental and resistant cells were treated with BYL719 (2 μM for T47D and 5 μM for MCF7), AEW541 (1 μM), or the combination BYL719/AEW541 for 72 hours. Percentage of apoptotic (Annexin V-positive and PI-negative) and necrotic cells (Annexin V and PI-positive) are indicated. (PDF 158 kb) [file 13058_2016_697_MOESM4_ESM.pdf]

# Leroy *et al.* Additional file 5

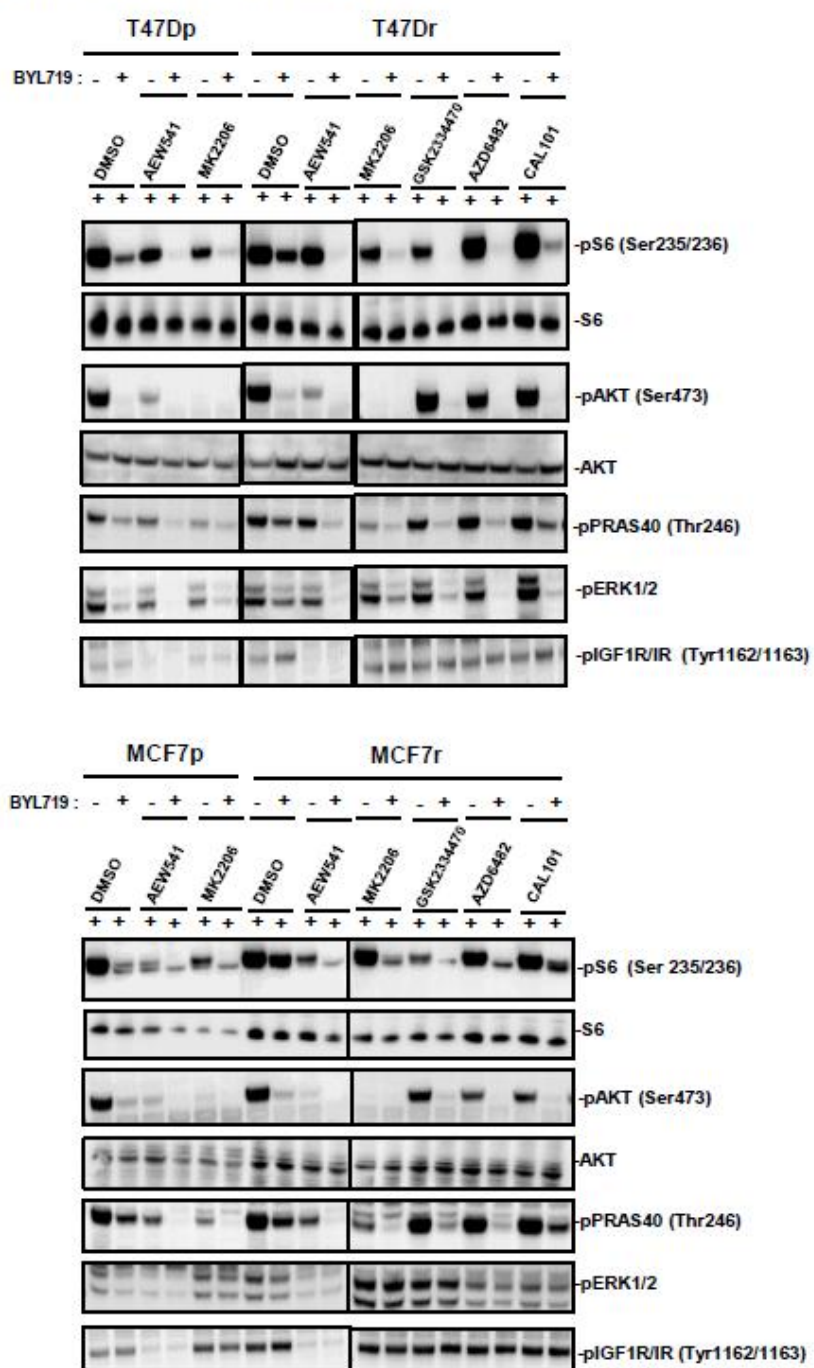

Supplement: Additional file 5: — is a figure showing that activation of IGF1R/p110β/AKT/mTOR produces resistance to BYL719. Immunoblots of lysates from parental and resistant cells treated for 24 hours with the respective IC90 concentrations of BYL719 and/or 1 μM of the indicated compounds. (PDF 142 kb) [file 13058_2016_697_MOESM5_ESM.pdf]

## Leroy *et al.* Additional file 6

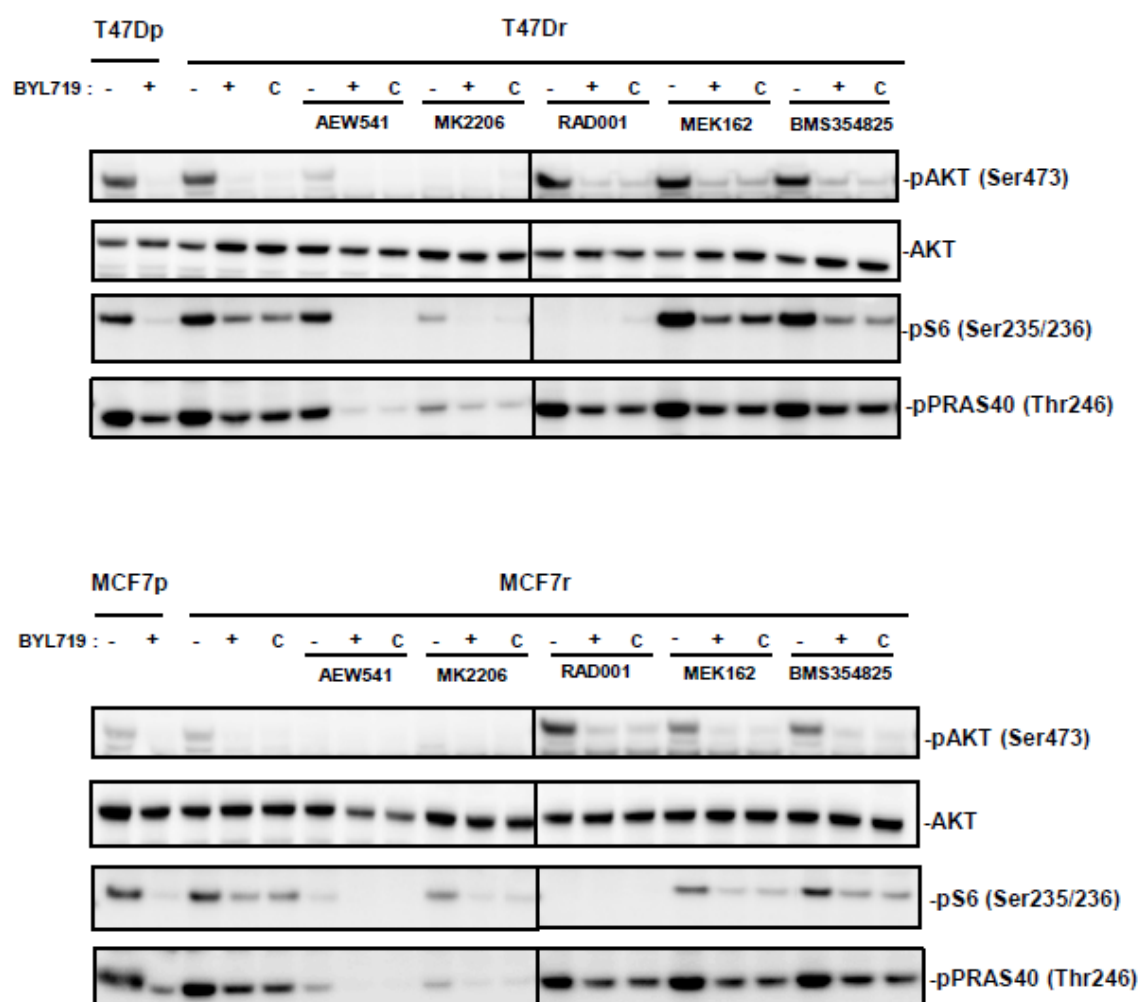

Supplement: Additional file 6: — is a figure showing that inhibition of MEK or SFK signaling pathways in combination with BYL719 does not alter mTOR-sustained activity in resistant cells. Immunoblots of lysates from parental and resistant cells treated for 24 hours with the respective IC90 concentration of BYL719 and/or 1 μM of AEW541, 1 μM MK2206, 10 nM RAD001, 500 nM MEK162, or 1 μM BMS354825. Immunoblots have been developed simultaneously with the same time of exposure. (PDF 176 kb) [file 13058_2016_697_MOESM6_ESM.pdf]

## Leroy *et al.* Additional file 7

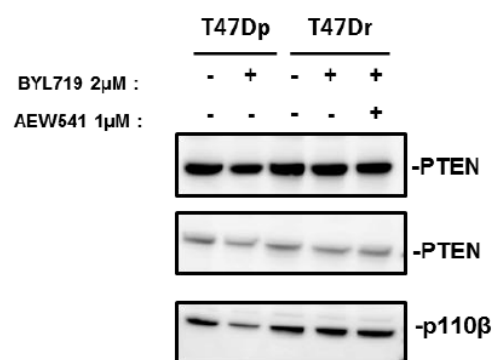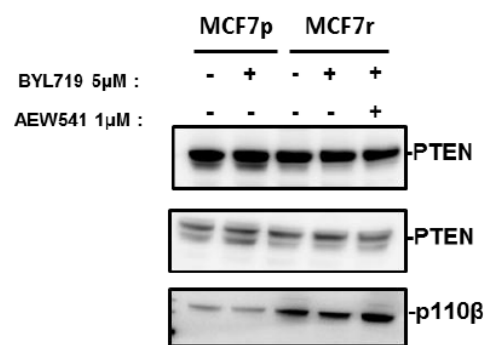

Supplement: Additional file 7: — is a figure showing that p110β activation does not correlate with loss of PTEN. Immunoblots of lysates from parental and resistant cells treated for 24 hours as indicated. BYL719 (IC90) and AEW541 (1 μM). (PDF 140 kb) [file 13058_2016_697_MOESM7_ESM.pdf]
