# Supplementary material for: Activation of IGF1R/p110β/AKT/mTOR confers resistance to α-specific PI3K inhibition
Source: Breast Cancer Res. 2016 Apr 5;18:41. doi: 10.1186/s13058-016-0697-1 (PMC4820873; doi:10.1186/s13058-016-0697-1)
Supplement: Additional file 2: — is a table presenting phosphopeptide intensity of T47Dp with insulin but without BYL719; and T47Dr with or without insulin treated for 24 hours with or without 2 μM BYL719. (PDF 258 kb) [file 13058_2016_697_MOESM2_ESM.pdf]

Genes names	Sequence	Variablemodifications(positiondescription)	Sum(ve131135_T47D_Resitante_1)	Sum(ve131136_T47D_Resitante_2)	T47D Resistant -INS +BYL	Sum(ve131138_T47D_Res_BYL_INS1)	Sum(ve131139_T47D_Res_BYL_INS2)	T47D Resistant +INS +BYL	Sum(ve131141_T47D_Parent_INS1)	Sum(ve131142_T47D_Parent_INS2)	T47D Parental +INS -BYL	Sum(ve131144_T47D_Res_INS1)	Sum(ve131145_T47D_Res_INS2)	T47D Resistant +INS -BYL	log 2 Res +INS +BYL / Res -INS +BYL	log 2 Res +INS +BYL / Res +INS -BYL	log 2 Res / Par
ACAP2	WFSIQNNQLVYQK	Phospho(Y)	557.31	1153.42	855.365	1240.96	1332.29	1286.625	3649.09	3038.04	3343.565	3356.74	3963.58	3660.16	0.58898	-1.50832	0.13052
ACLY	TTDGVYEGVAIGGDR	Phospho(Y)	92.37	40.43	66.4	597.02	722.36	659.69	145.18	87.97	116.575	703.89	870.13	787.01	3.312533	-0.25459	2.755124
ACTG1	GYSFTTTAER	Phospho(Y)	3261.39	3576.39	3418.89	2314.39	2274.15	2294.27	9114.58	6517.32	7815.95	493.43	349.46	421.445	-0.57549	2.444619	-4.213
ADAM9	HVSPVTPPREVPIYANR	Phospho(ST)Phospho(Y)	832.66	1286.95	1059.805	2421.69	2831.09	2626.39	733.38	235.88	484.63	119.9	2360.86	1240.38	1.309282	1.082299	1.355827
ADAM9	FAVPTYAAK	Phospho(Y)	97.33	134.62	115.975	929.12	545.79	737.455	1223.27	613.21	918.24	298.52	512.56	405.54	2.668741	0.862711	-1.17903
AFAP1L2	VAQQPLSLVGCEVVPDPSPDHLYSFR	Phospho(Y)	0	290.78	145.39	145.15	534.11	339.63	672.25	1045.92	859.085	776.5	779.88	778.19	1.224036	-1.19616	-0.14268
AGR2	LAEQFVLLNLVYETTDK	Phospho(Y)	1953.84	7126.9	4540.37	2174.17	2200.92	2187.545	29999.76	33802.6	31901.18	2321.14	2314.9	2318.02	-1.0535	-0.08358	-3.78264
AGR2	HLSPDGQYVPR	Phospho(Y)	1682.75	1395.71	1539.23	33.71	51.2	42.455	924.23	6205.93	3565.08	25.35	36.4	30.875	-5.18013	0.459495	-6.85135
AHCY	FDNLYGCR	Phospho(Y)	98.32	69.32	83.82	13.29	156.6	84.945	904.56	75.47	490.015	85.97	180.67	133.32	0.019235	-0.65029	-1.87793
AKR7A2	FYAYNPLAGGLLTGK	Phospho(Y)	1	1	1	5.33	0.32	2.825	364.03	368.31	366.17	1	1	1	1.498251	1.498251	-8.51637
ANKS1A	EEDEHPYELLLTAETK	Phospho(Y)	10428.31	7778.3	9103.305	37858.67	37161.19	37509.93	5127.67	5493.27	5310.47	20763.95	25965.66	23364.81	2.04281	0.682936	2.137426
ANKS1A	EEDEHPYELLLTAETKK	Phospho(Y)	3877.87	4931.91	4404.89	1001.33	896.67	949	2427.11	2820.79	2623.95	1825.85	1385.27	1605.56	-2.21463	-0.7586	-0.70866
ANKS1A	IHGSAAREEDEHPYELLLTAETK	Phospho(Y)	21985	25021.39	23503.2	1027.09	1205.89	1116.49	20817.57	20449.13	20633.35	2220.7	2555.43	2388.065	-4.39581	-1.09687	-3.11106
ANKS1A	IHGSAAREEDEHPYELLLTAETKK	Phospho(Y)	5647.32	5838.26	5742.79	44.85	78.77	61.81	6727.11	5456.97	6092.04	109.7	112.01	110.855	-6.53777	-0.84276	-5.78018
ANXA2P2	AYTNFDAER	Phospho(Y)	7574.59	7870.08	7722.335	48442.31	58111.5	53276.91	15963.53	12369.18	14166.36	9852.4	8536.29	9194.345	2.786401	2.534692	-0.62365
ANXA2P2	ALLYLCGGDD	Phospho(Y)	113.3	24.51	68.905	7.5	117.52	62.51	785.74	701.21	743.475	449.45	415.36	432.405	-0.14052	-2.79022	-0.7819
ANXA2P2	SLYYYIQQDTK	Phospho(Y)	2673.23	2154.04	2413.635	1902.81	2342.94	2122.875	3621.48	4331.88	3976.68	1419.04	1698.22	1558.63	-0.18519	0.445741	-1.35129
ANXA2P2	AYTNFDAERDALNIETAIK	Phospho(Y)	10945.08	19647.73	15296.41	7083.74	6621.99	6852.865	7418.06	7846.98	7632.52	2509.24	2389.6	2449.42	-1.15841	1.484267	-1.63972
ANXA5	LYDAYELK	Phospho(Y)	346.05	246.55	296.3	650.74	1218.13	934.435	1225.04	1127.81	1176.425	1005.76	866.2	935.98	1.657036	-0.00238	-0.32986
ARAP2	MQDIPIYANVHK	Phospho(Y)	146.8	338.26	242.53	2954.95	2607.44	2781.195	716.77	806.05	761.41	2511.06	4730.65	3620.855	3.51947	-0.38063	2.249585
ARHGAP35	NEEENIYSVPHDSTQGK	Phospho(Y)	195565.4	229272.9	212419.1	451618.9	290191	370905	434260.1	437032.6	435646.4	419403.5	493043	456223.2	0.804136	-0.29869	0.066582
ARHGEF10L	NLLYEDAHR	Phospho(Y)	761.82	701.2	731.51	6107.9	6387	6247.45	5101.17	5415.02	5258.095	4729.37	3400.09	4064.73	3.094318	0.620108	-0.37138
ARPC3	DTDIVDEAIYYFK	Phospho(Y)	55.12	238.99	147.055	1347.88	1222.93	1285.405	2354.55	1904.66	2129.605	2494.42	3485.91	2990.165	3.127795	-1.218	0.489639
ATP13A1	QFLPVAFPVGNAFSYYQSNR	Phospho(Y)	835.68	2916.62	1876.15	5595.56	6418.6	6007.08	15161.43	12712.84	13937.14	21689.72	27624.31	24657.02	1.678889	-2.03726	0.823064
ATP13A1	ATTGLKPVDSGCVAYVLR	Phospho(Y)	265.23	60.48	162.855	521.78	652.82	587.3	307.17	316.86	312.015	382.96	479.27	431.115	1.85051	0.446025	0.466457
ATP13A1	LGSTDLCYIAAVK	Phospho(Y)	193.21	33.89	113.55	315.34	135.79	225.565	517.58	123.58	320.58	257.69	436.05	346.87	0.990216	-0.62085	0.113711
ATP1A1	GIVVYTGDR	Phospho(Y)	63826.85	55940.78	59883.82	138181.2	131148.9	134665	132762.3	142016.3	137389.3	41695.27	37890.08	39792.68	1.169137	1.7588	-1.7877
ATP5B	VVDLLAPYAK	Phospho(Y)	129.43	214.14	171.785	1	1	1	488.8	384.47	436.635	0	1	1	-7.42446	0	-8.77028
ATP5H	LAALPENPPAIDWAYYK	Phospho(Y)	240.1	958.28	599.19	377.53	317.79	347.66	1372.04	1240.44	1306.24	515.63	268.57	392.1	-0.78534	-0.17354	-1.73613
BCAR1	DPLLEVYDVPPSVEK	Phospho(Y)	372.57	426.06	399.315	2831.64	3170.61	3001.125	122.06	201.33	161.695	1651.84	2339.92	1995.88	2.909904	0.588478	3.625678
BCAR1	RPGPGTLYDVPR	Phospho(Y)	26352	30636.04	28494.02	53772.5	40318.85	47045.68	24384.37	16742.8	20563.59	29474.01	26110.52	27792.27	0.723403	0.759379	0.434592
BCAR1	EETYDVPPAFAK	Phospho(Y)	129846.1	208556	169201	221763.9	222019.3	221891.6	132481.6	128419.9	130450.7	148565.4	165057.6	156811.5	0.391117	0.500824	0.265526
BCAR1	HLLAPGPQDIYDVPPVR	Phospho(Y)	132267.7	127131.3	129699.5	198461.6	180746.2	189603.9	140631.5	125335.9	132983.7	144361	173846.3	159103.6	0.547816	0.253022	0.258717
BCAR1	GLPPSNHHAVYDVPPSVSK	Phospho(Y)	46472.51	55838.28	51155.4	78990.44	88002.13	83496.29	73849.98	64340.27	69095.13	79811.49	84555.79	82183.64	0.706826	0.022861	0.250267
BCAR1	AQQGLYQVPGPSPQFQSPPAK	Phospho(Y)	69624.97	78338.33	73981.65	124065.3	97748.38	110906.8	110381	124577.3	117479.2	127412	142858.9	135135.5	0.584109	-0.28506	0.202001
BCAR1	VLPPEVADGGVVDSGVYAVPPPAER	Phospho(Y)	152219.2	168798.6	160508.9	184533.6	178544.9	181539.2	139889.6	133804.4	136847	134580.5	159264.9	146922.7	0.177628	0.305224	0.102494
BCAR3	CLEEHYGTSPGQAR	Phospho(Y)	0	14.56	7.28	106.95	0	53.475	17.45	0	8.725	155.39	0	77.695	2.876854	-0.53896	3.154595
BCAR3	VPSSPSAWLNSEANYCELNPAFATGCGR	Phospho(Y)	4002.06	18940.34	11471.2	9884.18	8429.05	9156.615	6871.29	6416.48	6643.885	5779.48	6477.16	6128.32	-0.32513	0.579323	-0.11654
BCR	GHGQPGADAEKPFYVNVEFHHER	Phospho(Y)	4312.15	5689.44	5000.795	23083.38	26281.21	24682.3	18228.42	9292.75	13760.59	13992.64	9344.54	11668.59	2.303247	1.080846	-0.23791
C11orf52	SPGLMSEDSNLHYADIQVCSRPHAR	Phospho(Y)	1377.1	1707.04	1542.07	97430.97	101366.9	99398.95	1873.9	1100.73	1487.315	23249.79	24054.51	23652.15	6.01029	2.071259	3.991189
C11orf52	HVHLENATEYATLR	Phospho(Y)	27472.14	31115.42	29293.78	146694.4	145711.5	146202.9	40212.19	34366.85	37289.52	27723.44	23665.97	25694.71	2.319306	2.508429	-0.5373
C9orf78	NAEDCLYELPENIR	Phospho(Y)	882.09	301.89	591.99	2494.06	2247.44	2370.75	4486.14	4174.6	4330.37	4311.49	5006.69	4659.09	2.001699	-0.9747	0.105558
CALCR	AAAAAAEAGDIPIYICHQELR	Phospho(Y)	2654.1	2758.05	2706.075	5525.64	4762.39	5144.015	1999.61	2076.6	2038.105	2329.47	2269.61	2299.54	0.926693	1.16155	0.174117
CALM2	VFDKDGNGYISAAELR	Phospho(Y)	91.04	690.67	390.855	1022.97	1114.32	1068.645	1461.52	1288.6	1375.06	1290.32	901.26	1095.79	1.451077	-0.03619	-0.32752
CALR	FYALSASFEPFSNK	Phospho(Y)	717.85	1324.91	1021.38	74.27	109.08	91.675	3656.2	3425.19	3540.695	34.24	0	17.12	-3.47785	2.420846	-7.69221
CALR	VHVIFNYK	Phospho(Y)	57.02	144.82	100.92	13.71	0	6.855	2920.72	2999.98	2960.35	0	0	1	-3.87991	2.777157	-11.5316
CANX	APVPTGEVYFADSFDR	Phospho(Y)	508.63	910.26	709.445	175.3	443.76	309.53	1212.37	1855.29	1533.83	1097.55	1248.38	1172.965	-1.19661	-1.92201	-0.38698
CARKD	IGVVGGCQEYTGAPYFAAISALK	Phospho(Y)	12464.34	45246.28	28855.31	37031.8	30948.16	33989.98	27027.48	22747.74	24887.61	24385.39	30345.29	27365.34	0.236273	0.31276	0.136922
CASKIN2	NTYNQTALDIVNQFTTSQASR	Phospho(Y)	3380.69	9818.2	6599.445	13861.98	14647.66	14254.82	9760.92	8195.78	8978.35	13981.12	13319.35	13650.24	1.111033	0.062524	0.604404
CBL	IKPSSSANAIYSLAARPLPVPK	Phospho(Y)	269.33	862.31	565.82	321.64	295.78	308.71	1959.93	1554.53	1757.23	605.28	453.41	529.345	-0.87409	-0.77796	-1.73102
CCT8	HFSGLEEAVYR	Phospho(Y)	162.15	41.79	101.97	21.07	14.64	17.855	3161.73	3073.76	3117.745	990.8	98.37	544.585	-2.51374	-4.93076	-2.51727
CDK1	IGEGTYGVVYK	Phospho(Y)	605207.4	578017.2	591612.3	3511062	5441101	4476082	7046212	6086052	6566132	6522314	6288063	6405188	2.919512	-0.517	-0.0358
CDK1	IGEGTYGVVYK	Phospho(ST)Phospho(Y)	668.43	731.04	699.735	297.89	465.16	381.525	5863.23	4251.47	5057.35	3141.04	5141.03	4141.035	-0.87503	-3.44014	-0.28839
CDK1	IGEGTYGVVYKGR	Phospho(Y)	980.72	1700.68	1340.7	502.83	473.23	488.03	14955.38	11579.14	13267.26	2342.45	1019.37	1680.91	-1.45794	-1.7842	-2.98056
CDK5	IGEGTYGTVFK	Phospho(Y)	1262.93	2215.44	1739.185	6498.96	7395.17	6947.065	6778.81	4791.22	5785.015	4102.58	2418.1	3260.34	1.997992	1.091381	-0.8273
CDV3	LQLDNQYAVLENQK	Phospho(Y)	947.43	506.05	726.74	3026.28	2833.78	2930.03	1447	1918.88	1682.94	3159.48	3627.95	3393.715	2.011404	-0.21195	1.011882
CDV3	KTPQGPPEIYSDTQFPSLQSTAK	Phospho(Y)	30937.2	39364.49	35150.85	53724.46	59683.96	56704.21	49299.65	50785.96	50042.81	62687.74	72300.29	67494.02	0.689896	-0.2513	0.431597
CFL1	HELQANCYEEVKDR	Phospho(Y)	28310.8	41791.2	35051	69615.55	69484.85	69550.2	56470.95	49501.96	52986.46	51892.38	36557.41	44224.9	0.988599	0.653196	-0.26076
CFL1	YALYDATYETK	Phospho(Y)	6053.05	7819.3	6936.175	6705.08	8026.77	7365.925	35394.74	27153.4	31274.07	16386.22	13981.34	15183.78	0.086726	-1.04359	-1.04244
CLDN3	VVYSAPR	Phospho(Y)	1398.36	1223.47	1310.915	5715.89	5036.04	5375.965	13452.33	12476.14	12964.24	4191.01	2649.56	3420.285	2.03595	0.652407	-1.92235
CLTC	ALEHFTDLYDIKR	Phospho(Y)	1319.6	1366.72	1343.16	3462.38	3386.7	3424.54	5415.57	4554.06	4984.815	3610.58	3612.19	3611.385	1.350279	-0.07664	-0.46499
CLTC	FNALFAQGNYSEAAK	Phospho(Y)	708.08	362.73	535.405	117.73	4.6	61.165	1709.03	1334.66	1521.845	20.11	11.54	15.825	-3.12985	1.950501	-6.58747
CLTC	LLYNNVSNFGR	Phospho(Y)	936.42	519.49	727.955	21.75	1	10.875	2179.95	2708.45	2444.2	0	49.88	24.94	-6.06476	-1.19745	-6.61476
CLTC	VANVELYYR	Phospho(Y)	31.45	0.09	15.77	4.84	1	2.42	1177.43	882.73	1030.08	1	1	1	-2.7041	1.275007	-10.0085
COX7A2	GGVADALLYR	Phospho(Y)	276.74	167.39	222.065	1	1	6.575	421.05	420.86	420.955	0	1	1	-5.07785	2.716991	-8.71752
CREBBP	AHLLAEKIYK	Phospho(Y)	4519.03	4398.41	4458.72	5934.11	6631.89	6283	627.49	480.34	553.915	662.33	711.11	686.72	0.494824	3.19366	0.310057
CREBBP	GHLLAEKIYK	Phospho(Y)	1612.42	3019.13	2315.775	3041.63	3762.47	3402.05	5028.48	4888.99	4958.735	1660.37	3215.82	2438.095	0.554909	0.48065	-1.02422
CRIP2	GVNTGAVGSYIYDRDPEGK	Phospho(Y)	324.23	583.71	453.97	1089.03	1267.66	1178.345	456.25	853.19	654.72	857.83	745.66	801.745	1.376093	0.555547	0.292265
CRIP2	GVNIGGAGSYIYEKPLAEGPQVTGPIEVPAAR	Phospho(Y)	5826.16	27591.97	16709.07	22025.98	22015.27	22020.63	15088.13	13351.86	14220	11222.77	14384.11	12803.44	0.398224	0.782324	-0.15139
CRK	YRPASASVSALIGGNQEGSHPQPLGGPEPGPYAQPSVNTPLPNLQNGPIYAR	Phospho(Y)	61074.69	240119.7	150597.2	231367.5	222427	226897.3	339273.2	309730.6	324501.9	410154.6	457167.2	433660.9	0.591344	-0.93453	0.418341
CSNK2A1	EPFFHGHDNYDQLVR	Phospho(Y)	506.61	227.43	367.02	2164.14	1745.77	1954.955	916.38	441.49	678.935	343.66	199.08	271.37	2.413205	2.848802	-1.32301
CSNK2A2	REPFFHGQDNYDQLVR	Phospho(Y)	822.85	740.58	781.715	2619.31	1634.5	2126.905	1046.01	962.88	1004.445	581.74	884.09	732.915	1.444041	1.537038	-0.45468
CTNNA1	QLQQAVTGISNAAQATASDDASQHQGGGGGELAYALNNFDK	Phospho(Y)	447.38	3463.02	1955.2	4678.62	4400.27	4539.445	4726.36	5624.29	5175.325	4809.4	5440.42	5124.91	1.2152	-0.17501	-0.01412
CTNNA1	NAGNEQDLGIQYK	Phospho(Y)	482.28	558.89	520.585	1009.05	1148.87	1078.96	823.71	372.43	598.07	609.46	261.84	435.65	1.051436	1.3084	-0.45714
CTNND1	APSRQDVYGPQPQVR	Phospho(ST)Phospho(Y)	121.01	95.29	108.15	607.19	869.35	738.27	238.13	179.82	208.975	891.34	913.57	902.455	2.771115	-0.28971	2.110525
CTNND1	QDVYGPQPQVR	Phospho(Y)	1619.74	1455.29	1537.515	4083.89	4188.95	4136.42	2549.51	1956.92	2253.215	3654.16	1831.85	2743.005	1.427782	0.592625	0.283772
CTNND1	LNGPQDHSHLLYSTIPR	Phospho(Y)	4652.95	5948.62	5300.785	9060.98	8808.98	8934.98	15612.01	12608.4	14110.21	8786.45	5995.62	7391.035	0.753258	0.273688	-0.93289
CTNND1	APSRQDVYGPQPQVR	Phospho(Y)	43996.14	43543.29	43769.72	2196.5	2562.99	2379.745	10237.5	9308.69	9773.095	5118.92	4001.98	4560.45	-4.20105	-0.93837	-1.09964
CTNND1	QDVYGPQPQVR	Phospho(Y)	683.42	243.42	463.42	2260.71	1353.37	1807.04	650.2	334.7	492.45	1957.3	1771.89	1864.595	1.963236	-0.04523	1.920813
CTNND1	FHPEPYGLEDDQR	Phospho(Y)	0	26.51	13.255	109.75	706.23	407.99	187.9	94.48	141.19	414.73	19.15	216.94	4.943925	0.911238	0.619658
CTNND1	SLDNNYSTPNER	Phospho(Y)	2943.5	3234.28	3088.89	7194.39	7602.72	7398.555	6106.73	6005.08	6055.905	1413.4	1991.94	1702.67	1.260155	2.119445	-1.83054
CTTN	LPSSPVYEDAASFK	Phospho(ST)Phospho(Y)	17.79	73.44	45.615	185.03	796.66	490.845	89.59	88.58	89.085	1239.09	1854.35	1546.72	3.427687	-1.65587	4.117886
CTTN	LPSSPVYEDAASFK	Phospho(Y)	12970.08	18386.69	15678.39	55473.17	55948.04	55710.61	18252.62	21193.4	19723.01	51731.8	62433.74	57082.77	1.829175	-0.0351	1.533176
CTTN	VDQSAVGFEYQGK	Phospho(Y)	805.91	818.89	812.4	895.65	1554.05	1224.85	1194.25	1068.8	1131.525	1076.51	772.29	924.4	0.592343	0.406016	-0.29168
CTTN, Y446	GPVSGTEPEPVYSMEAADYR	Phospho(Y)	246.46	405.33	325.895	35369.31	36790.89	36080.1	1347.1	1709.86	1528.48	33248.46	35167.88	34208.17	6.790652	0.076862	4.484171
CYTL1	EPGPLPVLLLLLAGAPAARPTPPTCYSRMRALSQEITR	Phospho(ST)Phospho(ST)Phosph	27180.99	35411.26	31296.13	8902.16	9952.54	9427.35	14345.91	11300.91	12823.41	23736.79	18264.78	21000.79	-1.73106	-1.15552	0.711663
DBNL	FQDVGPQAPVGSVYQK	Phospho(Y)	1027.51	1085.22	1056.365	4425.94	4459.47	4442.705	2745.11	4374.81	3559.96	1364.53	1517.04	1440.785	2.07233	1.624583	-1.30501
DCBLD2	AGKPGLPAPDELVYQVPQSTQEVSGAGR	Phospho(Y)	5766.62	7207.68	6487.15	6613.94	5620.07	6117.005	12315.58	9744.65	11030.12	14452.87	16820.94	15636.91	-0.08476	-1.35406	0.503507
DDR1	NLYAGDYYR	Phospho(Y)Phospho(Y)	19.11	19.67	19.39	0	14.54	7.27	715.26	330.13	522.695	545.73	0	272.865	-1.41529	-5.23009	-0.93778
DDX6	FGHLGLAINLITYDDRFNLK	Phospho(ST)	0	52.01	26.005	438.54	4.18	221.36	50.25	1283.87	667.06	0	0	1	3.089534	7.790251	-9.38167
DHX9	QLYHLGVVEAYSGLTK	Phospho(Y)	191.63	809.78	500.705	117.03	145.33	131.18	454.97	524.82	489.895	0	0	1	-1.93241	7.035404	-8.93633
DIABLO	AVYTLTSLYR	Phospho(Y)	181.93	180.27	181.1	130.91	183.14	157.025	276.05	286.83	281.44	371.38	523.43	447.405	-0.20579	-1.51059	0.668754
DLG1	NTSDFVYLK	Phospho(Y)	197.15	73.36	135.255	297.07	284.21	290.64	480.21	580.96	530.585	908.52	1306.73	1107.625	1.103551	-1.93016	1.061814
DLG3	RDNEVDGQDYHFVVSR	Phospho(Y)	5665.88	7844.18	6755.03	11387.74	17462.61	14425.18	3876.78	4213.06	4044.92	4235.91	4099.62	4167.765	1.094555	1.791243	0.043163
DNAJA1	NVVHQLSVTLEDLYNGATR	Phospho(Y)	1.03	1.42	1.225	104.19	564.83	334.51	352.29	313.3	332.795	792.97	695.32	744.145	8.093124	-1.15353	1.16095
DNAJC5	EATDTPIVIQPASATETTQLTADSHPSYHTDGFN	Phospho(ST)	1064.38	910.36	987.37	2506.98	2788.58	2647.78	1173.93	1614.75	1394.34	1403.84	2595.83	1999.835	1.423121	0.404902	0.520299
DSTN	YALYDASFETK	Phospho(Y)	1019.01	1085.05	1052.03	594.04	908.84	751.44	3597.08	4172.41	3884.745	553.34	586.4	569.87	-0.48545	0.399025	-2.76912
DYRK1A	IYQYIQSR	Phospho(Y)	228946.3	379801.6	304373.9	1147952	1192202	1170077	930275.6	1123083	1026679	765494.7	887109.3	826302	1.942687	0.501863	-0.31324
DYRK2	VYTYIQSR	Phospho(Y)	598.92	393.86	496.39	4148.14	4406.15	4277.145	8465.38	7478.91	7972.145	6413.76	4714.78	5564.27	3.107102	-0.37954	-0.51878
ECHS1	LFYSTFATDDRK	Phospho(Y)	1.18	58.43	29.805	72	55.46	63.73	693.64	1165.04	929.34	6.37	59.87	33.12	1.096418	0.94427	-4.81043
EEF1A2	EHALLAYTLGVK	Phospho(Y)	62247.91	54210.22	58229.07	61830.82	62927.2	62379.01	69247.1	64503.65	66875.38	52135.78	58169.22	55152.5	0.099321	0.177634	-0.27805
EEF1A2	STTTGHLIYK	Phospho(Y)	13791.04	13030.73	13410.89	14676.83	10705.27	12691.05	61072.73	65198.98	63135.86	10422.39	5946	8184.195	-0.07959	0.632899	-2.94755
EEF1A2	LPLQDVYK	Phospho(Y)	1139.99	1194.62	1167.305	581.44	679.19	630.315	5658.44	4098.78	4878.61	176.6	187.02	181.81	-0.88904	1.793641	-4.74597
EEF1G	AAGTLYTYPENWR	Phospho(Y)	5488.93	6258.17	5873.55	1226.95	1329.79	1278.37	12110.19	10878.87	11494.53	1229	1322.13	1275.565	-2.19993	0.003169	-3.17174
EEF1G	ALIAAQYSGAQVR	Phospho(Y)	603.72	762.48	683.1	25.03	63.9	44.465	3144.74	2480.97	2812.855	20.52	15.94	18.23	-3.94135	1.286356	-7.26958
EEF2	EDLYLKPIQR	Phospho(Y)	1238.75	1253.7	1246.225	1636.6	1165.35	1400.975	5324	3243.21	4283.605	481.98	608.36	545.17	0.168867	1.361653	-2.97405
EEF2	ALLELQLEPEELYQTFQR	Phospho(Y)	1	1	1	17.92	9.22	13.57	623.29	0	311.645	1	1	1	3.762349	3.762349	-8.28376
EFHD1	VFNPYTEFPEFSR	Phospho(Y)	13255.36	15863.96	14559.66	18386.38	17134.34	17760.36	23574.73	22272.33	22923.53	10261.56	11865.58	11063.57	0.286684	0.682844	-1.05101
EFS	DALEVYDVPPTALR	Phospho(Y)	4729.29	3973.32	4351.305	8979.24	8855.35	8917.295	7203.97	6836.52	7020.245	13851.11	17479.66	15665.39	1.035158	-0.8129	1.157987
EFS	VPSSGPYDCPASFSHPLTR	Phospho(Y)	2171.34	2768.14	2469.74	4748.35	6261.57	5504.96	7574.59	6888.38	7231.485	8188.24	8014.41	8101.325	1.156373	-0.55743	0.163866
EGFR	RPAGSVQNPVYHNQPLNPAPSR	Phospho(Y)	2212.81	1815.41	2014.11	8859.03	10289.38	9574.205	2059.02	1067.98	1563.5	2422.48	1674.22	2048.35	2.24901	2.22469	0.389683
EGFR	GSHQISLDNPDYQQDFFPK	Phospho(Y)	2290.8	2821.16	2555.98	3378.43	4101.31	3739.87	1182.49	869.11	1025.8	881.79	1264.31	1073.05	0.549112	1.801271	0.064968
EIF3A	FNVLQYVVPEVK	Phospho(Y)	323.28	425.33	374.305	297.27	524.4	410.835	390.63	875.01	632.82	403.37	641.82	522.595	0.134345	-0.34713	-0.2761
EIF5A	NDFQLIGIQDGYLSLLQDSGEVR	Phospho(Y)	401.55	750.5	576.025	931.06	778.71	854.885	1727.93	1888.58	1808.255	1055.1	1745.97	1400.535	0.569599	-0.71218	-0.36862
ELAVL1	NVALLSQLYHSPAR	Phospho(Y)	1120.75	984.65	1052.7	1140.25	1558.01	1349.13	2877.81	2669.72	2773.765	1314.71	1649.78	1482.245	0.357935	-0.13575	-0.90406
ENO1	AAVPSGASTGIYEALELR	Phospho(Y)	14727.63	20608.54	17668.09	13610.98	14606.8	14108.89	19212.19	19159.33	19185.76	12144.75	13475.78	12810.27	-0.32454	0.139304	-0.58274
ENO1	IGAEVYHNLK	Phospho(Y)	2087.08	1387.99	1737.535	112.27	84.3	98.285	3329.79	2871.5	3100.645	151.11	158.05	154.58	-4.14393	-0.65331	-4.32614
ENO1	YISPDQLADLYK	Phospho(Y)	645.47	499.52	572.495	55.36	16.55	35.955	2993.59	2938.39	2965.99	50.52	28.96	39.74	-3.993	-0.1444	-6.22178
EPB41L1	IRPGEYEQFESTIGFK	Phospho(Y)	118.21	213.67	165.94	186.82	307.93	247.375	163.55	141.2	152.375	492.25	438.05	465.15	0.576038	-0.911	1.61007
EPHA1	LLDDFDGTYETQGGK	Phospho(Y)	2402.94	3095.19	2749.065	14064.62	12445.1	13254.86	6554.35	6511.87	6533.11	4848.68	5657.32	5253	2.269509	1.335308	-0.31463
EPHA1	LWLKPYVDLQAYEDPAQGALDFTR	Phospho(Y)	0	598.96	299.48	964.17	911.04	937.605	0	659	329.5	0	45.07	22.535	1.646521	5.378741	-3.87004
EPHA2	VLEDDPEATYTTSGGK	Phospho(ST)	5963.66	4504.39	5234.025	7533.49	7369.49	7451.49	4589.21	5224.69	4906.95	6232.57	5094.77	5663.67	0.509608	0.395792	0.206911
EPHA4	VLEDDPEAAYTTR	Phospho(Y)	15138.74	14517.99	14828.37	13653.76	14507.78	14080.77	14601.48	14806.24	14703.86	3591.58	3141.5	3366.54	-0.07463	2.064388	-2.12686
EPHA4	TYVDPFTYEDPNQAVR	Phospho(Y)	22504.19	19353.37	20928.78	12614.47	11408.29	12011.38	5209.33	3689.74	4449.535	198.94	534.06	366.5	-0.80109	5.034445	-3.60177
EPHA4	TYVDPFTYEDPNQAVR	Phospho(ST)Phospho(Y)	3396.05	7490.37	5443.21	1183.21	1785.8	1484.505	287.78	568.99	428.385	0	0	1	-1.87448	10.53577	-8.74276
EPHB3	FLEDDPSDPTYTSSLGGK	Phospho(Y)	633.47	761.05	697.26	1097.08	1838.79	1467.935	717.06	677.42	697.24	1497.76	2191.63	1844.695	1.074019	-0.32959	1.403655
ERBB2	LLDIDETEYHADGGKVPIK	Phospho(Y)	3283.54	4391.35	3837.445	6033.81	5762.04	5897.925	2921.63	5109.58	4015.605	4626.07	4725.07	4675.57	0.620061	0.335065	0.219525
ERBB2	GTPTAENPEYLGLDVPV	Phospho(Y)	13497.07	20258.4	16877.74	23733.41	24791.27	24262.34	5841.14	5895.27	5868.205	5361.86	6277.35	5819.605	0.523597	2.059726	-0.012
ERBB2IP	AQIPEGDYLSYR	Phospho(Y)	4822.04	5987.71	5404.875	11841.87	15115.17	13478.52	9043.86	8985.05	9014.455	10622.75	10170.89	10396.82	1.318329	0.37452	0.20583
ERBB2IP	RAQIPEGDYLSYR	Phospho(Y)	2804.89	2379.01	2591.95	2968.62	4599.98	3784.3	752.41	785.3	768.855	119.08	170.62	144.85	0.545989	4.707395	-2.40815
ERBB3	SLEATDSAFDNPDYWHSR	Phospho(Y)	618.01	126.36	372.185	1467.88	1694.51	1581.195	218.05	72.08	145.065	258.05	278.49	268.27	2.086923	2.559258	0.886986
ERBB3	AFQGPGHQAPHVHYAR	Phospho(Y)	2765.82	1228.94	1997.38	3686.05	3577.19	3631.62	819.08	673.9	746.49	1258.75	652.36	955.555	0.862504	1.926202	0.356216
ESYT1	HLSPYATLTVGDSSHK	Phospho(Y)	1149.65	890.35	1020	2732.69	3995.62	3364.155	3433.31	3989.89	3711.6	4335.64	4276.97	4306.305	1.721675	-0.35621	0.214409
F11R	VIYSQPSAR	Phospho(Y)	1953.6	988.97	1471.285	9301.73	11376.29	10339.01	16424.23	17979.81	17202.02	5471.26	3801.71	4636.485	2.812949	1.156995	-1.89147
FAM83F	AKAPAPAPAESGESLAYWPDR	Phospho(Y)	789.99	648.76	719.375	7780.3	9611.36	8695.83	2225.68	2293.3	2259.49	8817.02	10356.26	9586.64	3.595508	-0.1407	2.085028
FBL	DHAVVVGVYRPPPK	Phospho(Y)	2050.72	2183.55	2117.135	10.62	26.84	18.73	5233.61	3874.44	4554.025	0	0	1	-6.82062	4.227279	-12.1529
FGB	YRGTAGNALMDGASQLMGENR	Phospho(Y)Phospho(ST)	935.96	194.64	565.3	2329.44	412.15	1370.795	98.04	619.99	359.015	1004.8	4815.28	2910.04	1.277924	-1.08603	3.018923
FKBP4	FEIGEGENLDLPYGLER	Phospho(Y)	1457.86	4344.9	2901.38	1365	1967.47	1666.235	6643.23	6299.45	6471.34	1846.21	1571.65	1708.93	-0.80015	-0.0365	-1.92097
FKBP4	VFVHYTGWLLDGTK	Phospho(Y)	34.46	200.7	117.58	64.53	0	32.265	2015.99	616.07	1316.03	0	159.62	79.81	-1.8656	-1.3066	-4.04348
FKBP5	FGIEPNAELIYEVTLK	Phospho(Y)	1086.6	2626.76	1856.68	1127.1	834.11	980.605	3384.98	3521.97	3453.475	1590.22	1385.28	1487.75	-0.92098	-0.60139	-1.21492
FRK	HGHYFVALFDYQAR	Phospho(Y)	9333	16729.3	13031.15	16935.41	18226.54	17580.98	14224.68	12931.02	13577.85	11658.98	14968.41	13313.7	0.432051	0.401104	-0.02834
FRS2	LVYENINGLSIPSASGVR	Phospho(Y)	1951.37	3062.55	2506.96	4747.96	4311.33	4529.645	1464.59	5090.15	3277.37	6346.24	6583.66	6464.95	0.853459	-0.51324	0.980101
FYN	LIEDNEYTAR	Phospho(Y)	25750.5	31934.51	28842.51	81016.67	72760.62	76888.65	183542.9	189464	186503.4	142807.9	122364.2	132586	1.414574	-0.78609	-0.49227
FYN	KLDNGGYYITTR	Phospho(Y)	2626.01	2356.23	2491.12	2262.57	2717.96	2490.265	3132.13	2704.79	2918.46	2560.65	1447.64	2004.145	-0.0005	0.313312	-0.54222
FYN	LDNGGYYITTR	Phospho(Y)	1983.54	1597.87	1790.705	2522.58	4209.35	3365.965	4726.8	4629.87	4678.335	3457.19	2718	3087.595	0.910492	0.124537	-0.59951
G6PD	VGFQYEGTYK	Phospho(Y)	3048.76	2756.82	2902.79	5508.51	4747.04	5127.775	9396.15	8834.54	9115.345	6086.18	10910.22	8498.2	0.820893	-0.72882	-0.10114
G6PD	RVGFQYEGTYK	Phospho(Y)	993.02	1215.09	1104.055	849.39	964.05	906.72	1447.09	1628.8	1537.945	371.87	58.61	215.24	-0.28408	2.074711	-2.83699
GAB1	DASSQDCYDIPR	Phospho(Y)	40.69	42.13	41.41	1143.5	939.97	1041.735	1813.33	886.52	1349.925	1135.07	1584.85	1359.96	4.652865	-0.38458	0.010685
GAB1	APSASVDSSLYNLPR	Phospho(Y)	388.4	285.78	337.09	3017.12	2570.33	2793.725	3304.93	3132.79	3218.86	2424.95	3290.55	2857.75	3.050984	-0.03269	-0.17167
GAPDH	LISWYDNEFGYSNR	Phospho(Y)	1430.89	1454.71	1442.8	401.38	509.47	455.425	21792.8	21066.19	21429.5	2848.01	3118.83	2983.42	-1.66359	-2.71168	-2.84456
GINS2	EETFTPMPSPYYMELTK	Phospho(ST)Phospho(ST)	8520.14	2363.57	5441.855	180.18	202.51	191.345	1994.46	3684.8	2839.63	813.21	833.9	823.555	-4.82985	-2.10569	-1.78577
GIT1	LOPFHSTELEDDAIYSVHVPAGLYR	Phospho(Y)	1037.38	7586.34	4311.86	7027.5	5558.9	6293.2	13604.59	12650.55	13127.57	8211.91	8507.42	8359.665	0.545483	-0.40965	-0.65108
GIT1	LOPFHSTELEDDAIYSVHVPAGLYR	Phospho(Y)	6760.88	26251.54	16506.21	28614.24	22350.04	25482.14	41695.97	33375.11	37535.54	25785.62	27520.79	26653.21	0.626478	-0.06482	-0.49395

GLUD1, Y512	DIVHSGLAYTMER	Phospho(Y)	315.09	278.72	296.905	21065.25	22491.87	21778.56	294.88	318.23	306.555	10348.39	7411.11	8879.75	6.196763	1.294318	4.856301
GOLGA4	EAEEKILTLENQVYSMK	Phospho(Y)Phospho(ST)	1410.74	1047.97	1229.355	5201.84	4282.72	4742.28	337.03	186.58	261.805	107.43	77.74	92.585	1.947679	5.678659	-1.49964
GOLGA5	NTDYTELHQQNTDLIYQTGPK	Phospho(Y)	316.1	1243.54	779.82	1667.86	1027.02	1347.44	2788.57	2756.59	2772.58	3709.23	4060.82	3885.025	0.789008	-1.5277	0.486695
GPRC5C	VPSEGAYDIILPR	Phospho(Y)	3821.85	4319.4	4070.625	9527.24	8131.7	8829.47	7126.58	6796.35	6961.465	17572.55	18749.64	18161.1	1.117077	-1.04045	1.383388
GPRC5C, Y414	AEDMYSAQSHQAATPPK	Phospho(Y)	28.7	25.28	26.99	17.64	945.17	481.405	0.08	10.02	5.05	35.96	799.44	417.7	4.156754	0.204784	6.37004
GSK3A	GEPNVSYICSR	Phospho(Y)	1026708	1045290	1035999	2561566	3229987	2895777	2539680	2243350	2391515	2709005	3148900	2928952	1.482927	-0.01643	0.29246
GSTM3	LDLDFPNLPYLLDGK	Phospho(Y)	296.74	1681.84	989.29	247.16	160.21	203.685	5994.59	7802.81	6898.7	80.2	23.04	51.62	-2.28005	1.980338	-7.06225
HARS	LIYDLKDQGGELLSLR	Phospho(Y)	129.44	381.68	255.56	566.56	456.52	511.54	943.39	1112.85	1028.12	1251.48	1125.91	1188.695	1.001185	-1.21646	0.20937
HDAC10	EEALTALGKLLYLLDGMLDGQVNSGIAATPASAAAATLDVAVR	Phospho(ST)Phospho(ST)Phosph	14123.24	77.28	7100.26	2569.54	5.37	1287.455	127.66	337.64	232.65	3.66	9832.74	4918.2	-2.46335	-1.93361	4.401897
HGS	VCEPCYEQLNR	Phospho(Y)	678.83	547.77	613.3	1332.55	1653.31	1492.93	1289.89	2236.67	1763.28	3135.74	3022.82	3079.28	1.283482	-1.04445	0.804331
HIPK2	AVCSTYLQSR	Phospho(Y)	7324.2	13430.08	10377.14	33199.71	36946.7	35073.21	58612.42	58952.81	58782.62	38507.25	22821.32	30664.29	1.75696	0.19381	-0.93883
HIPK3	TVCSTYLQSR	Phospho(Y)	3046.99	2813.96	2930.475	10460.44	8345.75	9403.095	14585.69	16586.3	15586	10777.3	6535.07	8656.185	1.682001	0.119404	-0.84845
HIST1H2BO	ESYSIYVYK	Phospho(Y)	0.03	101.09	50.56	0	2.81	1.405	675.87	935.65	805.76	29.71	0	14.855	-5.16935	-3.40231	-5.76133
HIST1H4A	ISGLIYEETR	Phospho(Y)	3489.32	5720.37	4604.845	960.43	1059.85	1010.14	10686.24	11096.41	10891.33	1163.23	1296.29	1229.76	-2.1886	-0.28382	-3.14673
HIST2H2BF	ESYSVYVYK	Phospho(Y)	1171.98	1058.33	1115.155	61.95	121.45	91.7	4039.43	2827.18	3433.305	48.08	63.09	55.585	-3.60418	0.722226	-5.94876
HMGB1	RPPSAFFLFCSEYRPK	Phospho(Y)	1002.57	1164.63	1083.6	520.91	479.6	500.255	1024.7	1227.18	1125.94	349.04	376.27	362.655	-1.1151	0.464066	-1.63446
HNRNPA2B1	NMGGPYGGGNYGPGGSGGSGGYGGR	Phospho(Y)	434.85	32.75	233.8	7682.35	6136.64	6909.495	185.19	32.76	108.975	3867.96	6372.52	5120.24	4.885233	0.432369	5.554142
HNRNPH1	ATENDIYNFFSPLNPVR	Phospho(Y)	371.94	832.98	602.46	302.49	495.38	398.935	1123.95	1117.97	1120.96	176.76	454.07	315.415	-0.59471	0.338902	-1.82941
HSP90AA1	HIYYITGETK	Phospho(Y)	1997.15	1894.81	1945.98	800.14	1020.52	910.33	11998.72	11751.55	11875.14	1292.41	1312.94	1302.675	-1.09604	-0.51702	-3.18839
HSP90AA1	HSQFIGYPITLFVEK	Phospho(Y)	1275.65	2892.98	2084.315	22.51	136.14	79.325	4652.19	4712.02	4682.105	5.17	60.68	32.925	-4.71565	1.268592	-7.15183
HSP90AB1	HSQFIGYPITLYLEK	Phospho(Y)	152.12	593.41	372.765	0	193.39	96.695	2097.84	1820.59	1959.215	51.78	0	25.89	-1.94675	1.901046	-6.24174
HSP90AB2P	SIYYITGESK	Phospho(Y)	79.01	49.76	64.385	35.78	220.82	128.3	1107.15	777.25	942.2	58.08	138.5	98.29	0.994725	0.384405	-3.26092
HSP90B1	DISTNYYASQK	Phospho(Y)	777.02	606.09	691.555	251.56	331.5	291.53	5997.24	5692.6	5844.92	1506.12	828.92	1167.52	-1.2462	-2.00173	-2.32374
HSPA1B	AAAIGIDLGTTYSCVGVFQHGK	Phospho(ST)	890.11	1910.66	1400.385	956.49	1064.08	1010.285	511.58	1485.67	998.625	1211.12	1603.19	1407.155	-0.47106	-0.47802	0.494766
HSPA1B	ELEQVCNPIISGLYQGAGGPGPGGFGAQGPK	Phospho(Y)	6822.74	20000.54	13411.64	5171.9	4774.59	4973.245	26685.17	23218.32	24951.75	6874.02	8028.53	7451.275	-1.43123	-0.5833	-1.74358
HSPA1B	TTPSYVAFTDTER	Phospho(Y)	1214.82	2188.9	1701.86	50.76	95.7	73.23	5224.06	3533.91	4378.985	249.61	201.93	225.77	-4.53853	-1.62435	-4.27767
HSPA4	KEDIYAVEIVGGATR	Phospho(Y)	1152.71	713.54	933.125	2975.13	3203.36	3089.245	1271.07	1366.61	1318.84	4511.61	4943.81	4727.71	1.727112	-0.61389	1.841872
HSPA5	VTHAVVTVPAYFNDAQR	Phospho(Y)	3131.7	5029.25	4080.475	41.47	26.71	34.09	4918.44	5041.02	4979.73	173.35	153.61	163.48	-6.90324	-2.26169	-4.92888
HSPA5	ITPSYVAFTPEGER	Phospho(Y)	2247.51	3157	2702.255	39.68	25.54	32.61	4044.47	3266.52	3655.495	0	23.1	11.55	-6.37271	1.497422	-8.30603
HSPA8	GPAVGIDLGTTYSCVGVFQHGK	Phospho(Y)	5188.22	9526.98	7357.6	4784.76	3948.48	4366.62	8587.5	8294.6	8441.05	4545.07	4688.01	4616.54	-0.75272	-0.08029	-0.87061
IFNGR1	APTSFGYDKPHVLVDLLVDDSGK	Phospho(Y)	0	88.57	44.285	750.03	700.52	725.275	271.24	185.13	228.185	1401.01	1555.24	1478.125	4.033638	-1.02717	2.695492
IFNGR1	YVSLITSYQPFSLEK	Phospho(Y)	83.27	128.79	106.03	489.03	151.38	320.205	544.6	315.25	429.925	1397.43	1163.03	1280.23	1.594523	-1.99934	1.574246
IFT27	ASDGAHFQKSYTLTTGMDLVVK	Phospho(Y)Phospho(ST)	913514.9	1467596	1190555	1974534	1829400	1901967	1044428	901030.5	972729.4	1078583	1065152	1071868	0.675857	0.827365	0.140016
IFT27	RSDGAHFQKSYTLTTGMDLVVK	Phospho(Y)	95811.36	68854.25	82332.81	94588.34	96029.84	95309.09	76183.4	70256.49	73219.95	62626.72	62750.86	62688.79	0.211146	0.604406	-0.22403
IFT27	SSDGAHFQKSYTLTTGMDLVVK	Phospho(Y)Phospho(ST)	99037.39	113027.8	106032.6	177111.2	193532	185321.6	102558.4	96195.84	99377.1	80060.66	82101.53	81081.1	0.805523	1.192593	-0.29355
IGF1R	DIYETDYYRK	Phospho(Y)	1583.98	880.76	1232.37	10142.41	13158.36	11650.39	8963.77	8837.25	8900.51	6635.37	6330.58	6482.975	3.24087	0.84565	-0.45723
IGF1R	DIYETDYYRK	Phospho(Y)Phospho(Y)	111.57	46.45	79.01	10489.05	12786.73	11637.89	17339.31	15480.49	16409.9	6034.02	4415.23	5224.625	7.202579	1.15543	-1.65117
INPPL1	LYEWISIDKDEAGAK	Phospho(Y)	55.48	99.65	77.565	1141.58	1161.49	1151.535	501.5	385.7	443.6	512.95	631.98	572.465	3.892009	1.008299	0.367928
INPPL1	ERLYEWISIDKDEAGAK	Phospho(Y)	1075.68	1058.29	1066.985	5805.62	5049.9	5427.76	1883.35	2794.32	2338.835	2213.35	2305.39	2259.37	2.346817	1.264436	-0.04987
INPPL1	NSFNNPAYYVLEGVPHQLLPPEPPSPAR	Phospho(Y)	18635.14	46350.92	32493.03	82508.59	86023.18	84265.89	62508.41	54781.74	58645.08	47148.36	47281.85	47215.11	1.374818	0.8357	-0.31276
IQGAP1	LGLAPQIQDLYGK	Phospho(Y)	464.03	301.33	382.68	560.36	612.77	586.565	473.15	515.71	494.43	562.91	889.75	726.33	0.616152	-0.30833	0.554859
IRS1	GEEELSNYICMGGK	Phospho(Y)	0	46.64	23.32	1852.14	2318.04	2085.09	0	11.95	5.975	19.37	0	9.685	6.482398	7.750142	0.696813
IRS1, Y612	GGHHRPDSSTLHTDDGYMPMSPGVAPVPSGR	Phospho(Y)	92.39	73.34	82.865	21832.47	27538.35	24685.41	110.04	33.27	71.655	4531.03	2225.43	3378.23	8.21868	2.869319	5.559056
ITGB1	WDTGENPIYK	Phospho(Y)	843.54	1042.31	942.925	1517.81	1722.87	1620.34	2468.87	3786.57	3127.72	2218.63	1678.7	1948.665	0.781082	-0.26619	-0.68263
ITSN2	EEPEALYAAVNK	Phospho(Y)	1507.22	1516.13	1511.675	7041.33	7638.71	7340.02	2564.84	2056.62	2310.73	2887.2	3100.02	2993.61	2.279636	1.293898	0.373538
ITSN2	LIYLVPEK	Phospho(Y)	573.86	463.64	518.75	1411.23	1777.01	1594.12	2448.71	3309.26	2878.985	1981.05	2324.04	2152.545	1.619649	-0.43328	-0.41952
ITSN2	REEPEALYAAVNK	Phospho(Y)	51867.21	63752.17	57809.69	343264.7	371810.1	357537.4	107743	125338.8	116540.9	49301.76	50150.33	49726.05	2.628711	2.846021	-1.22876
KATNAL2	GLLLYGPPGTGK	Phospho(Y)	1255.84	1263.07	1259.455	222.69	411.87	317.28	2521.66	3455.48	2988.57	366.87	571.45	469.16	-1.98897	-0.56432	-2.6713
KIAA1217	NEGFYADPYLYHEGR	Phospho(Y)	14512.91	15822.69	15167.8	19133.82	16315.6	17724.71	14519.47	16355	15437.24	15103.34	16717.74	15910.54	0.22475	0.155779	0.043568
KIAA1217	NVYYELNDVR	Phospho(Y)	12449.06	13281.08	12865.07	23642.27	22194.53	22918.4	19700.15	17036.06	18368.11	12050.75	13947.26	12999.01	0.833047	0.818105	-0.4988
LASP1	RPLEQQQPHHIPTSAPVYQQPQQQPVAQSYGGYKEPAAPVSIQR	Phospho(Y)	13101.59	17245.83	15173.71	20450.77	16132.34	18291.56	19921.26	16948.91	18435.09	17984.22	16921.81	17453.02	0.269604	0.067701	-0.07898
LDHA	DQLIYNLLKEEQTPQNK	Phospho(Y)	5988.93	10236.19	8112.56	7706.51	8436.08	8071.295	13730.97	12188.49	12959.73	10881.79	11905.78	11393.79	-0.00736	-0.49738	-0.18579
LDHA	DQLIYNLLK	Phospho(Y)	3444.84	4107.81	3776.325	3405.39	3155.39	3280.39	5753.9	5714.14	5734.02	3290.36	3566.75	3428.555	-0.20312	-0.06373	-0.74195
LGALS3	VAVNDAHLLQYNHR	Phospho(Y)	2645.32	2374.84	2510.08	0	44.75	22.375	2635.06	2435.5	2535.28	0	21.5	10.75	-6.8097	1.057551	-7.88166
LMNA	NIYSEELR	Phospho(Y)	860.61	566.11	713.36	229.43	141.56	185.495	2555.8	2887.75	2721.775	163.74	48.7	106.22	-1.94325	0.804325	-4.67942
LPHN1	NPLQGYYQVR	Phospho(Y)	867.62	993.28	930.45	4863.72	5858.77	5361.245	4030.93	4605.74	4318.335	5760.96	3856.46	4808.71	2.526568	0.156918	0.155175
LPP	NDSDPTYGQQGHPNTWK	Phospho(Y)	26148.69	23930.36	25039.53	61025.47	71349.76	66187.62	27184.05	19858.31	23521.18	31951.64	30901.34	31426.49	1.402354	1.07458	0.418021
LTF	YYGYTGAFR	Phospho(Y)	1128.01	732.95	930.48	334.46	584.1	459.28	7344.4	6507.84	6926.12	1744.45	1610.18	1677.315	-1.0186	-1.86871	-2.04589
LYN	VIEDNEYTAR	Phospho(Y)	464.83	136.74	300.785	2115.94	3732.45	2924.195	5357.83	5273.63	5315.73	4160.88	3568.16	3864.52	3.281235	-0.40225	-0.45998
LYN	TNADVMTALSQGYRMPR	Phospho(ST)Phospho(ST)Phosph	6510.91	2903.93	4707.42	65.33	155.39	110.36	1374.06	2969.24	2171.65	688.52	713.28	700.9	-5.41465	-2.66699	-1.63151
MAGED2	VPNSNPPEYEFFWGLR	Phospho(Y)	106.85	89.47	98.16	492.25	0	246.125	32.15	74.49	53.32	0.95	0	0.475	1.326184	9.017248	-6.81061
MAPK1	VADPDHDHTGFLTEYVATR	Phospho(Y)	31561.99	30579.51	31070.75	305446.8	244054.8	274750.8	219866.2	253479.8	236673	152994.7	177526.5	165260.6	3.144495	0.733381	-0.51815
MAPK13, Y182	HADAEMTGYVVTR	Phospho(Y)	517.93	661.94	589.935	35614.86	38638.12	37126.49	735.43	1008.83	872.13	8545.38	7604.75	8075.065	5.975749	2.200903	3.210859
MAPK14, Y182	HTDDEMTGYVATR	Phospho(Y)	793.32	799.24	796.28	138832.3	143726.1	141279.2	2962.81	3287.09	3124.95	66229.55	53186.11	59707.83	7.471058	1.242557	4.256015
MAPK3	IADPEHDHTGFLTEYVATR	Phospho(Y)	24424.09	39793.72	32108.91	225727.2	155520	190623.6	120710.9	111495.2	116103	103527.5	89621.68	96574.58	2.569682	0.981012	-0.26569
MDH2	GYLGPEQLPDCLK	Phospho(Y)	4391.37	5348.21	4869.79	1628.3	1448.1	1538.2	4715.58	4987.26	4851.42	672.95	727.84	700.395	-1.66262	1.135002	-2.79217
MPP5	VYESIGQYGGETVK	Phospho(Y)	39.61	41.73	40.67	100.13	0	50.065	209.6	0	104.8	703.3	0	351.65	0.299837	-2.81227	1.746502
MPP7	NNYYGTSIDSVR	Phospho(Y)	285.53	137.4	211.465	478.07	305.91	391.99	1417.1	1183.63	1300.365	275.71	161.96	218.835	0.890398	0.840973	-2.571
MPZL1	SLPSGSHQGPVIYAQLDHSGGHHSDK	Phospho(Y)	3314.42	4272.52	3793.47	9663.31	9662.77	9663.04	26504.24	17616.38	22060.31	12394.28	12571.85	12483.07	1.348959	-0.36942	-0.82148
MPZL1	SESVVYADIR	Phospho(Y)	1099.78	1305.03	1202.405	5208.82	3967.87	4588.345	17663.5	10196.4	13929.95	3489.8	5756.78	4623.29	1.932051	-0.01095	-1.5912
MSN	APDFVFYAPR	Phospho(Y)	554	411.94	482.97	483.26	628.07	555.665	326.65	370.5	348.575	233.71	436.34	335.025	0.202282	0.729947	-0.0572
MSN	KAPDFVFYAPR	Phospho(Y)	830.79	755.15	792.97	709.97	826.21	768.09	1286.38	1148.14	1217.26	707.72	824.51	766.115	-0.04599	0.003714	-0.668
MST1R	DILDREYYSVQQHR	Phospho(Y)	0	21.4	10.7	658.18	675.58	666.88	963.16	935.8	949.48	324.54	7.54	166.04	5.961744	2.005896	-2.51561
MYH9	ALELDSNLYR	Phospho(Y)	356.45	261.92	309.185	354.22	227.85	291.035	1880.07	1893.42	1886.745	860.49	847.08	853.785	-0.08728	-1.55268	-1.14395
MYO1D	HIDYFNNQIIVDLVEQQHK	Phospho(Y)	0.22	463.53	231.875	658.52	589	623.76	47.17	48.16	47.665	0	22.82	11.41	1.427644	5.77262	-2.06263
NCK1, Y105	LYDLNMPAYVK	Phospho(Y)	455.82	358.85	407.335	23331.54	28461.31	25896.43	1189.51	1081.04	1135.275	19272.26	17503.73	18388	5.990393	0.493989	4.01765
NCKIPSD	QHSLPSSEHLGADGGLYQIPPQPR	Phospho(Y)	33468.79	43102.21	38285.5	94905.49	84252.24	89578.87	59712.58	58460.66	59086.62	69562.83	75925.06	72743.95	1.22636	0.300331	0.299996
NEDD9	HQSLSPNHPPPQLGQSVGSQNDAYDVPR	Phospho(Y)	187.66	1607.64	897.65	254	774.27	514.135	27.14	0	13.57	16.43	22	19.215	-0.80401	4.741842	0.501812
NEDD9	LYQVPNPQAAPR	Phospho(Y)	517.2	472.2	494.7	511.41	306.18	408.795	21.44	25.19	23.315	17.25	33.65	25.45	-0.27518	4.00564	0.126407
NEDD9	QAGRPDLRPEGVYDIPPTCTKPAGK	Phospho(Y)	7361.19	6130.17	6745.68	1160.17	1209.01	1184.59	220.7	169.14	194.92	29.2	42.77	35.985	-2.50958	5.040848	-2.43741
NEDD9	ANPQERDGVYDVPLHNPPDAK	Phospho(Y)	7684.74	9485.35	8585.045	816.91	570.4	693.655	337.81	296.45	317.13	56.42	31.14	43.78	-3.62954	3.985874	-2.85673
NEDD9	DGVYDVPLHNPPDAK	Phospho(Y)	13923.55	17102.34	15512.95	21850.74	22250.55	22050.65	2137.23	1734.44	1935.835	130.82	247.17	188.995	0.507348	6.866329	-3.35654
NEDD9	RHQSLSPNHPPPQLGQSVGSQNDAYDVPR	Phospho(Y)	3989.04	5964.25	4976.645	1388.34	2896.91	2142.625	96.6	101.53	99.065	1.69	1.19	1.44	-1.21579	10.53909	-6.10423
NUMA1	VESLESLYFTPIPAR	Phospho(Y)	2020.41	2256.58	2138.495	1803.31	1219.02	1511.165	3727.4	3228.06	3477.73	2594.67	3025.44	2810.055	-0.50093	-0.89494	-0.30755
ORAI1	GDHPLTPGSHYA	Phospho(Y)	1036.47	909.13	972.8	4463.97	2777.05	3620.51	2111.92	1107.81	1609.865	2017.07	1546.68	1781.875	1.895978	1.022797	0.146456
PABPC4	ALYDTFSAFGNILSCK	Phospho(Y)	137.3	79	108.15	0.04	79.93	39.985	1912.7	1487.7	1700.2	45.59	170.53	108.06	-1.4355	-1.4343	-3.9758
PAG1	AEFAEYASVDR	Phospho(Y)	125.38	125.29	125.335	278.89	164.11	221.5	820.25	355.95	588.1	610.42	941.71	776.065	0.821517	-1.80887	0.400116
PARD3	ERDYAEIQDFHR	Phospho(Y)	1084.07	878.42	981.245	4271.3	3188.16	3729.73	1346.29	827.72	1087.005	228.09	394.8	311.445	1.926386	3.582022	-1.80331
PARD3B	DHLEGLYAK	Phospho(Y)	417.03	412.63	414.83	2995.48	2463.59	2729.535	842.66	299.15	570.905	1245.85	1289.76	1267.805	2.718063	1.106322	1.15101
PARD3B	DGHPLSPERDHLEGLYAK	Phospho(Y)	1013.62	1267.42	1140.52	1633.45	1465.6	1549.525	432.4	414.2	423.3	493.84	56.8	275.32	0.442134	2.492645	-0.62057
PDCD6IP	LANQAADYFGDAFK	Phospho(Y)	795.6	792.38	793.99	75.7	184.03	129.865	1823.21	1431.91	1627.56	307.36	432.24	369.8	-2.61211	-1.50973	-2.13789
PDIA3	ELSDFISYLQR	Phospho(Y)	0	53.64	26.82	29.65	0	14.825	41.43	1099.85	570.64	0	38.11	19.055	-0.85528	-0.36214	-4.90434
PDIA3	IFRDGEEAGAYDGPR	Phospho(Y)	9.84	157.3	83.57	16.11	22.54	19.325	27.48	1464.01	745.745	9	0	4.5	-2.11252	2.102472	-7.37261
PDIA4	VEGFPTIYFAPSGDK	Phospho(Y)	70.75	116.42	93.585	79.4	71.21	75.305	279.59	940.77	610.18	1	1	1	-0.31353	6.234674	-9.25309
PEAK1	FNSYNNAGMPPFPIIIHDEPTYAR	Phospho(Y)	337.81	246.17	291.99	3506.03	2942.19	3224.11	0	61.77	30.885	1720.08	2156.64	1938.36	3.46491	0.734064	5.971786
PEAK1	VPIVINPNAYDNLAIYK	Phospho(Y)	7856.02	17979.21	12917.62	26870.13	24650.44	25760.29	12297.82	12669.83	12483.83	9177.23	9936.56	9556.895	0.995809	1.430535	-0.38545
PEAK1	NAIKVPIVINPNAYDNLAIYK	Phospho(Y)	666.36	3580.57	2123.465	1321.23	1163.76	1242.495	2571.53	2247.68	2409.605	591.72	1098.63	845.175	-0.77318	0.555918	-1.51147
PEAK1	VPIVINPNAYDNLAIYK	Phospho(Y)Phospho(Y)	0	1284.18	642.09	1530.9	1062.7	1296.8	0	3114.45	1557.225	289.55	253.78	271.665	1.014109	2.255055	-2.51908
PGAM1	FSGWYDADLSPAGHEEAK	Phospho(Y)	3988.88	3676.76	3832.82	5750.26	6792.21	6271.235	5798.66	6074.86	5936.76	6170.54	5812.54	5991.54	0.710343	0.065823	0.013251
PGAM1	HYGGLTGLNK	Phospho(Y)	2246.44	2042.7	2144.57	8833.53	10899.13	9866.33	6881.07	6549.37	6715.22	4990.55	2727.15	3858.85	2.201825	1.354343	-0.79926
PGK1	KELNYFAK	Phospho(Y)	3985.16	5696.5	4840.83	10047.21	9601.79	9824.5	11898.09	11978.13	11938.11	4395.62	4025.52	4210.57	1.02113	1.222368	-1.50349
PGRMC2	FYGPAGPYGIFAGR	Phospho(Y)	189.78	297.5	243.64	277.26	201.82	239.54	2108.92	2029.71	2069.315	254.12	139.79	196.955	-0.02448	0.282401	-3.39322
PIK3CB	VPFILTYDFIHVIQQGK	Phospho(Y)	514.22	2651.13	1582.675	16902.37	20908.47	18905.42	10135.52	9367.3	9751.41	4237.95	5196.24	4717.095	3.578363	2.002829	-1.04771
PIK3R1, Y580	DQYLMWLTQK	Phospho(Y)	176.02	521.98	349	16455	17673.8	17064.4	91.45	737.92	414.685	2146.54	3118.55	2632.545	5.611619	2.69646	2.66637
PIN4, Y122	FGYHIIMVEGR	Phospho(Y)	28.96	17.48	23.22	1280.61	1176.2	1228.405	6.04	4.5	5.27	879.36	1019.43	949.395	5.725275	0.371706	7.493062
PKM	EAEAAIYHLQLFEELR	Phospho(Y)	1743.53	5883.56	3813.545	1471.77	1577.97	1524.87	7337.95	6652.35	6995.15	1155.85	1224.78	1190.315	-1.32245	0.357343	-2.55501
PKP3	GQYHTLQAGFSSR	Phospho(Y)	4014.72	2939.54	3477.13	12167.03	13117.1	12642.07	8494.82	9494.76	8994.79	6881.8	6462.64	6672.22	1.862263	0.921993	-0.43092
PKP3	NLIYDNADNK	Phospho(Y)	1345.89	1361.92	1353.905	5816.92	7564.15	6690.535	14026.93	14233.69	14130.31	3877.04	2566.47	3221.755	2.304995	1.054275	-2.13287
PKP3	ADYDTLSLR	Phospho(Y)	587.41	701.16	644.285	2886.9	3922.1	3404.5	1473.71	1492.04	1482.875	247.47	248.21	247.84	2.401672	3.779962	-2.58092
PKP4	SAVSPDLHITPIYEGR	Phospho(Y)	6164.99	5588.02	5876.505	9391.68	9687.73	9539.705	5870.88	5735.24	5803.06	11650.09	11539.21	11594.65	0.698986	-0.28144	0.998574
PKP4	VHFPASTDYSTQYGLK	Phospho(Y)	785.14	1405.18	1095.16	3004.57	3356.14	3180.355	651.36	926.15	788.755	1159.85	1246.07	1202.96	1.538046	1.402599	0.60894
PKP4	NNYALNTTATYAEPYRPIQYR	Phospho(Y)Phospho(Y)	1463.58	3030.7	2247.14	3380.66	2822.42	3101.54	1902.6	1918.31	1910.455	528.97	1018.86	773.915	0.464895	2.002738	-1.30367
PKP4	STTNYVDFYSTK	Phospho(Y)	1391.76	978.48	1185.12	1688.87	1650.05	1669.46	166.18	295.31	230.745	120.22	526.42	323.32	0.494348	2.368347	0.486663
PKP4	LQHQQLYYSQDDSNRK	Phospho(Y)	10737.64	12302.98	11520.31	23131.74	23166.42	23149.08	14671.26	13197.9	13934.58	17507.2	10242.33	13874.77	1.006775	0.738492	-0.00621
PKP4	NNYALNTTATYAEPYRPIQYR	Phospho(Y)	36599.23	53141.93	44870.58	51115.92	42614.46	46865.19	22632.64	24081.84	23357.24	18591.61	18782.8	18687.21	0.062747	1.326466	-0.32182
PKP4	TYYSPVYR	Phospho(Y)	792.16	617.27	704.715	1295.48	1771	1533.24	2167.18	1776.48	1971.83	1223.59	989.58	1106.585	1.121472	0.470469	-0.83342
PLCG2	RQEELNNQLFLYDTHQNLR	Phospho(Y)	108.82	25.21	67.015	1577.66	1661.02	1619.34	377.93	270.68	324.305	1336.19	799.75	1067.97	4.594778	0.600535	1.719448
PLEKHA6	SEDIYADPAAYVMR	Phospho(Y)	4596.67	3832.72	4214.695	81548.6	78601.89	80075.25	4524.71	3515.34	4020.025	63521.17	66548.85	65035.01	4.247856	0.30014	4.01594
PLEKHA6	LPPRSEDIYADPAAYVMR	Phospho(Y)	2199.56	1564.36	1881.96	12010.97	9950.51	10980.74	2024.04	2169.57	2096.805	12254.99	13088.49	12671.74	2.544667	-0.20664	2.59535
PLEKHA6	QPVYYDELDAASSSLR	Phospho(Y)	553.46	694.96	624.21	1919.48	2421.42	2170.45	229.63	522.18	375.905	896.09	806.78	851.435	1.797891	1.350026	1.179528
PLEKHS1	QTHLQDLSEATQDVKEENHYLTPR	Phospho(Y)	162.43	247.69	205.06	241.28	240.8	241.04	69.63	106.18	87.905	2781.85	2749.89	2765.87	0.233226	-3.52039	4.975644
PRKD2	SVVGTPAYLAPEVLLNQGYNR	Phospho(Y)	156.16	632.23	394.195	594.26	465.03	529.645	1034.61	1014.21	1024.41	1043.1	1099.15	1071.125	0.426116	-1.01603	0.064334
PRLR	LQLGGLDYLDPACFTHSFH	Phospho(Y)	9200.8	44662.85	26931.83	34824.32	32963.38	33893.85	81129.54	69266.72	75198.13	46812.69	52807.98	49810.34	0.331712	-0.55542	-0.59425
PRPF4B	LCDFGSASHVADNDITPYLVSR	Phospho(Y)	2172121	2477657	2324889	3554840	3386360	3470600	2798520	2696043	2747281	3173495	2855209	3014352	0.578023	0.203337	0.133843
PSMA2	LAQQYYLVYQEPIPTAQLVQR	Phospho(Y)	954.15	2587.85	1771	1476.89	1400.19	1438.54	3326.81	3625.72	3476.265	2690.12	2671.41	2680.765	-0.29996	-0.89804	-0.37489
PSMA2, Y76	HIGLVYSGMGPDYR	Phospho(Y)	3.64	0	1.82	289	414.02	351.51	27.77	80.91	54.34	630.85	654.67	642.76	7.593483	-0.87071	3.564194
PSMC5	GVLLYGPPGTGK	Phospho(Y)	7.95	15.86	11.905	17.59	128.6	73.095	68.56	1388.04	728.3	149.24	88.93	119.085	2.618205	-0.70415	-2.61254
PSMC6	GCLLYGPPGTGK	Phospho(Y)	564.52	537.73	551.125	220.13	271.03	245.58	2324.03	1522.65	1923.34	246.98	182.75	214.865	-1.16619	0.192763	-3.16211
PTBP1	NNQFQALLQYADPVSAQHAK	Phospho(Y)	2098.86	4595.99	3347.425	92.36	63.99	78.175	3805.53	3936.1	3870.815	0	71.1	35.55	-5.4202	1.136858	-6.76664
PTK2	FLIGNQHIYQPVGKPDPAAPPK	Phospho(Y)	92852.9	105004.4	98928.66	152848.5	135258.1	144053.3	95348.63	74630.01	84989.32	64694.19	70946.1	67820.15	0.542142	1.086816	-0.32557
PTK2	GSIDREDGSLQGPIGNQHIYQPVGKPDPAAPPK	Phospho(ST)Phospho(Y)	305.67	713.96	509.815	2476.06	1233.6	1854.83	519.41	619.9	569.655	2225.34	1328.42	1776.88	1.863241	0.061941	1.641186
PTK2	EDGSLQGPIGNQHIYQPVGKPDPAAPPK	Phospho(Y)	15617.29	17304.58	16460.94	53085.13	40526.64	46805.89	12224.56	11480.33	11852.45	22977.46	26502.88	24740.17	1.507644	0.919835	1.061671
PTK2	AAAYLDPNLNHTPNSSTK	Phospho(Y)	4176	3662.03	3919.015	5666.88	5164.55	5415.715	3748.69	4826.01	4287.35	5365.02	6842.13	6103.575	0.466661	-0.1725	0.509568
PTK2	GSIDREDGSLQGPIGNQHIYQPVGKPDPAAPPK	Phospho(Y)	162001.7	179779.7	170890.7	218083	189767.1	203925.1	267528.4	201236.8	234382.6	200904.5	168657	184780.8	0.254965	0.142225	-0.34305
PTK2	LSRGSIDREDGSLQGPIGNQHIYQPVGKPDPAAPPK	Phospho(ST)Phospho(Y)	5883.36	6720.3	6301.83	3358.67	3646.67	3502.67	6338.07	6620.44	6479.255	2403.6	2526.82	2465.21	-0.84732	0.506745	-1.39412
PTK2, Y576	YMEDSTYYK	Phospho(Y)	86.94	23.26	55.1	5238.91	6181.2	5710.055	3836	4431.13	4133.565	35673.14	28341.21	32007.18	6.695309	-2.48682	2.952937
PTK6	VSEKPSADYVLSVR	Phospho(Y)	1474.73	665.39	1070.06	1805.4	3278.44	2541.92	326.36	0	163.18	183.72	1042.53	613.125	1.248227	2.051665	1.909717
PTK6	LSSFTSYENPT	Phospho(Y)	6751.97	9569.01	8160.49	15794.04	18089.66	16941.85	59536.54	54371.82	56954.18	81197.18	63171.51	72184.35	1.053864	-2.09109	0.341884
PTPN11	IQNTGDYYDLYGGEK	Phospho(Y)	18192.05	18052.28	18122.17	45116.51	47258.78	46187.65	49104.6	25620.28	37362.44	46753.4	36837.41	41795.41	1.349752	0.144163	0.161756
PTPRA	VVQEYIDAFSDYANFK	Phospho(Y)	39701.66	95661.22	67681.44	116003.7	112985.9	114494.8	99036.5	89392.25	94214.38	119426.3	131379.7	125403	0.75845	-0.13129	0.412553
PTTG1IP	YGLFKEENPYAR	Phospho(Y)	1982.67	2548.77	2265.72	2242.8	2756.9	2499.85	6416.85	6520.06	6468.455	5641.21	3384.62	4512.915	0.141872	-0.85222	-0.51936
PXN	FIHQQPQSSSPVYGSSAK	Phospho(Y)	40823.69	41742.4	41283.05	94341.03	134529.7	114435.4	53915.76	55015.79	54465.78	62983.71	58637.75	60810.73	1.470912	0.912135	0.158976
PXN	LLLELNAVQHNPPGFPADEANSSPPLPGALSPLYGVPETNSPLGGK	Phospho(Y)	7942.6	23518.03	15730.32	25538.5	26596.87	26067.69	21828.23	20742.22	21285.23	16780.04	22047.72	19413.88	0.728715	0.425174	-0.13276
PXN	VGEEEHVYSFPNK	Phospho(Y)	1761703	1605501	1683602	2445925	2590868	2518396	1501079	1647813	1574446	1231312	1317517	1274414	0.580954	0.982671	-0.30501
PXN	FIHQQPQSSSPVYGSSAK	Phospho(ST)Phospho(Y)	4476.32	6335.94	5406.13	15188.3	17505.69	16347	14269.98	13966.68	14118.33	11707.64	8456.76	10082.2	1.596357	0.697215	-0.48576
RAB1B, Y7	MNPEYDYLFK	Phospho(Y)	1	1	1	2479.31	2799.4	2639.355	19.49	1	9.745	1428.21	2101.77	1764.99	11.36597	0.580525	7.500782
RAB2A	GAAGALLVYDITR	Phospho(Y)	571.88	613.86	592.87	45.04	0	22.52	993.25	1136.16	1064.705	28.1	0	14.05	-4.71844	0.680637	-6.24374
RAB7A	QETEVELYNEFPEPIK	Phospho(Y)	425.83	1097.57	761.7	2520.62	2691.44	2606.03	360.84	778.89	569.865	1735.64	1556.01	1645.825	1.774559	0.663043	1.530119
RAP1B, Y159	INVNEIFYDLVR	Phospho(Y)	8.78	471.01	239.895	39.14	163.88	101.51	2012.63	1871.57	1942.1	451.96	706.36	579.16	-1.24078	-2.51234	-1.74558
RARS	GNTAAYLLYAFTR	Phospho(Y)	221.35	315.9	268.625	258	477.16	367.58	581.74	871.97	726.855	659.69	894.6	777.145	0.452465	-1.08013	0.096516
RASAL2	RPLHPALNQPGGLQPLSFQNPVYHLNNPIPAMPK	Phospho(Y)	565.38	2225.41	1395.395	52402.92	40822.03	46612.48	5791.88	4651.07	5221.475	25327.69	25808.59	25568.14	5.061971	0.866369	2.291818
RBM14	ASYVAPLTAQPATYR	Phospho(Y)	1349.64	1338.25	1343.945	4023.11	4108.4	4065.755	3457.04	3983.02	3720.03	1242.65	2474.36	1858.505	1.597049	1.129381	-1.00117
REPS2	KDVLYSQPPSKPIR	Phospho(Y)	3875.79	3433.64	3654.715	8877.81	9256.67	9067.24	2818.96	2774.23	2796.595	3215.61	3366.21	3290.91	1.310905	1.462177	0.234815
RET	RPAQAFPVSYSSSGAR	Phospho(Y)	795.35	664.17	729.76	2180.91	2020.5	2100.705	2083.26	2319.04	2201.15	8554.01	9343.48	8948.745	1.52538	-2.09081	2.023428
RHOC	ISAFGYLECSAK	Phospho(Y)	156.99	238.55	197.77	1156.7	1116.99	1136.845	758.44	647.33	702.885	850.07	787.82	818.945	2.52314	0.473197	0.220478
RIN1	EKPAQDPLYDVPNASGGQAGGPQRPGR	Phospho(Y)	30894.61	36059.9	33477.26	55298.2	82793.89	69046.05	73972.17	66880.04	70426.11	52936.01	46624.18	49780.1	1.044378	0.47199	-0.50054
RPL15	QGYVIYR	Phospho(Y)	125.97	94.86	110.415	312.58	593.83	453.205	1058.64	1303.23	1180.935	618.55	568.83	593.69	2.037228	-0.38955	-0.99215
RPL31	LYTLVTYVPVTTFK	Phospho(Y)	780.32	1539.34	1159.83	1673.62	1323.41	1498.515	2130.68	2413.49	2272.085	828.2	1111.27	969.735	0.36962	0.627871	-1.22835
RPLP0	IIQLLDDYPK	Phospho(Y)	571.87	471.42	521.645	986.53	913.68	950.105	2748.5	2373.6	2561.05	2902.96	3084.34	2993.65	0.865019	-1.65575	0.22517
RPS13	GLSQSALPYR	Phospho(Y)	60.59	201.42	131.005	0	1	1	2577.7	1685.91	2131.805	44.09	0	22.045	-7.03348	-4.46238	-6.59548
RPS2	AFVAIGDYNGHVGLGVK	Phospho(Y)	8320.43	7089.76	7705.095	9126.75	10080.19	9603.47	29867.38	29342.34	29604.86	16824.25	22659.24	19741.75	0.317743	-1.03962	-0.58458
RPS27L	LVQSPNSYFMDVK	Phospho(Y)	237.89	246.77	242.33	15309.99	19935.85	17622.92	2828.63	2648.9	2738.765	22433.64	20222.73	21328.19	6.184336	-0.27531	2.961164
RPS29	GHQQLYWSHPR	Phospho(Y)	1102.35	933.86	1018.105	277.19	148.17	212.68	2798.54	1943.05	2370.795	315.02	113.03	214.025	-2.25913	-0.00909	-3.46952
RPS3	ELAEDGYSGVEVR	Phospho(Y)	43.9	410.71	227.305	0.03	31.41	15.72	1141.79	1078.73	1110.26	65.35	0	32.675	-3.85396	-1.05559	-5.08657
RPS7	VETFSGVYK	Phospho(Y)	81.34	27.57	54.455	0.01	0	0.005	1368.76	1125.18	1246.97	0	1	0.5	-13.4108	-6.64386	-11.2842
RPSAP58	ADHQPLTEASYVNLPTIALCNTDSPLR	Phospho(Y)	1216.72	6297.57	3757.145	6146.47	6053.12	6099.795	5785.75	4365.18	5075.465	4221.57	5318.48	4770.025	0.699124	0.354764	-0.08954
S100A11	DGYNYTLSK	Phospho(Y)	1609.81	1619.26	1614.535	2829.34	3602.12	3215.73	12403.82	10231.95	11317.89	722.53	455.46	588.995	0.994028	2.448819	-4.26421
SCAMP3	QYATLDVYNPFETR	Phospho(Y)	954.7	1963.28	1458.99	6403.01	7472.24	6937.625	4617.46	4587.04	4602.25	4830.13	6079.11	5454.62	2.249472	0.346963	0.245139
SCARB1	DKEAIQAYSESLMTSAPK	Phospho(Y)	0	27.21	13.605	0	125.51	62.755	0	1	1	330.39	30.04	180.215	2.205593	-1.52192	7.493575

SDC4	KAPTNEFYA	Phospho(Y)	1286.34	1141.75	1214.045	3793.69	3797.88	3795.785	4159.33	3271.56	3715.445	3635.48	2772.91	3204.195	1.644576	0.244436	-0.21357
SDCBP	LYPELSQYMGLSLNEEEIR	Phospho(Y)	34.64	32.72	33.68	41.38	93.27	67.325	32.11	20.59	26.35	389.15	899.11	644.13	0.99925	-3.25814	4.611477
SDCBP	VIQAQTAFSANPANPAILSEASAPIPHDGNLYPR	Phospho(Y)	268.38	1730.65	999.515	500.31	428.68	464.495	331.64	261.13	296.385	3466.45	3421.6	3444.025	-1.10557	-2.89036	3.538551
SEC16A	ANHSSHQEDTYGALDFTLSR	Phospho(Y)	632.33	455.26	543.795	1362.51	1410.57	1386.54	1486.06	1200.23	1343.145	2283.2	3555.9	2919.55	1.350354	-1.07426	1.120131
SEPT2	QQPTQFINPETPGYVGFANLPNQVHR	Phospho(Y)	4705.35	18359.68	11532.52	19972.88	16006.96	17989.92	12308.46	11137.27	11722.87	14080.81	14678.75	14379.78	0.641482	0.323147	0.294716
SEPT7	NLEGYVGFANLPNQVYR	Phospho(Y)	975.01	2915.34	1945.175	2814.3	1813.54	2313.92	2545.06	2063.36	2304.21	2498.8	2104.29	2301.545	0.250439	0.007736	-0.00167
SEPT7	LAAVTYNGVDNNK	Phospho(Y)	175.07	288.87	231.97	139.19	211.73	175.46	127.34	483.78	305.56	13.78	46.2	29.99	-0.4028	2.548589	-3.3489
SET	IDFYFDENPYFENK	Phospho(Y)	576.22	1573.25	1074.735	1054.2	786.69	920.445	1606.98	1742.5	1674.74	901.24	1333.32	1117.28	-0.22358	-0.27959	-0.58395
SGK223	QEDAPVVYLGSFR	Phospho(Y)	348.6	321.7	335.15	1033.81	716.6	875.205	246.31	411.2	328.755	530.71	543.01	536.86	1.384814	0.705075	0.707533
SGK223	LNLSHSETNVHDESHFSYSLSPGNR	Phospho(Y)	4755.29	10320.43	7537.86	8494.29	9715.04	9104.665	9530.15	8313.82	8921.985	10496.92	7379.1	8938.01	0.272451	0.026652	0.002589
SHANK2	YSLDSEDLYSR	Phospho(Y)	654.59	406.95	530.77	736.56	785.35	760.955	2192.27	2103.08	2147.675	3865.32	3058.1	3461.71	0.519724	-2.1856	0.688709
SHANK2, Y989	RGQMPENPYSEVGK	Phospho(Y)	107.79	105.46	106.625	9753.6	8805.06	9279.33	290.68	164.12	227.4	7062.99	5753.49	6408.24	6.443403	0.534092	4.816624
SHB	GESAGYMEPYEAQR	Phospho(Y)	0	76.77	38.385	7280.6	6850.37	7065.485	0	24.51	12.255	197.29	20.01	108.65	7.524102	6.023029	3.148246
SHB	VTIADDYSDPFDAK	Phospho(Y)	10472.97	12826.93	11649.95	33257.84	34803.65	34030.75	7110.87	9597.13	8354	5782.31	7285.37	6533.84	1.546515	2.380836	-0.35454
SHB	LDYCGGSGEPGGVQR	Phospho(Y)	14636.58	19976.34	17306.46	29617.28	31037.85	30327.57	1884.33	1569.99	1727.16	681.72	261.64	471.68	0.809319	6.006677	-1.87252
SHB	LPQDDDRPADEYDQPWEWNR	Phospho(Y)	2684.5	3081.88	2883.19	7191.2	8268.37	7729.785	1068.19	719.91	894.05	606.66	679.95	643.305	1.422762	3.586853	-0.47485
SHC1	ELFDDPSYVNVQNLDK	Phospho(Y)	24059.81	34169.18	29114.5	67417.02	75200.29	71308.66	42180.81	44599.32	43390.07	46092.36	59994.97	53043.67	1.29234	0.426897	0.289816
SLC25A4	AAYFGVYDTAK	Phospho(Y)	645.96	872.53	759.245	419.22	573.87	496.545	2801.7	2390.26	2595.98	1285.51	1236.68	1261.095	-0.61264	-1.34468	-1.0416
SLC25A5	AAYFGIYDTAK	Phospho(Y)	715.7	799.84	757.77	899.37	1245.42	1072.395	1279.38	1259.32	1269.35	1408.48	1556.28	1482.38	0.501004	-0.46708	0.223825
SMC3	LFYHIVDSDEVSTK	Phospho(Y)	388.6	31.75	210.175	343.66	78.24	210.95	1071.44	1694.69	1383.065	876.39	129.27	502.83	0.00531	-1.25317	-1.45973
SNRNP70	EFEVYGPIKR	Phospho(Y)	2827.28	2313.79	2570.535	4068.18	5513.95	4791.065	3446.18	3458.86	3452.52	6176.58	4574.7	5375.64	0.898278	-0.16609	0.638787
SPDEF	LVYQFVHPI	Phospho(Y)	118.47	158.17	138.32	108.28	145.97	127.125	947.96	875.11	911.535	715.04	589.29	652.165	-0.12176	-2.35899	-0.48306
SRCIN1	AAGGGGPLYGDGYGFR	Phospho(Y)	0	1	1	49.66	185.86	117.76	79.84	57.79	68.815	669.28	689.26	679.27	6.879706	-2.52814	3.30319
SRCIN1	EPLYAAFPGSHLTNGDLR	Phospho(Y)	6852.68	6133.8	6493.24	21314.74	23078.79	22196.77	13413.61	10070.91	11742.26	18847.64	25559.29	22203.47	1.773339	-0.00044	0.919075
SRCIN1	GEGLYADPYGLLHEGR	Phospho(Y)	9946.07	12510	11228.04	18122.19	16469.55	17295.87	10231.09	10315.36	10273.23	11525.66	12933.87	12229.77	0.623322	0.500031	0.251508
SRSF3	AFGYYGPLR	Phospho(Y)	13.8	33.08	23.44	13.22	99.36	56.29	3255.93	3696.26	3476.095	2038.99	2314.84	2176.915	1.263906	-5.27326	-0.67518
SRSF7	AFSYYGPLR	Phospho(Y)	52.7	59.89	56.295	284.85	315.46	300.155	2583.35	2419.25	2501.3	1423.4	2080.78	1752.09	2.414629	-2.5453	-0.5136
SSB	ICHQIEYYFGDFNLPR	Phospho(Y)	26.65	1	13.325	1	1	1	479.19	24.83	252.01	29.63	1	14.815	-3.73606	-3.88899	-4.08835
STAT3	YCRPESQEHPEADPGAAPYLK	Phospho(Y)	367.5	1149.06	758.28	2457.45	1890.96	2174.205	919.67	740.42	830.045	3416.69	4387.04	3901.865	1.519685	-0.84368	2.232902
STAT3	YCRPESQEHPEADPGSAAPYLK	Phospho(Y)	1460.2	1442.72	1451.46	8036.11	6810.16	7423.135	2772.54	2497.64	2635.09	8658.62	11914.62	10286.62	2.354524	-0.47067	1.964845
STAT5B	AVDGYVKPQIK	Phospho(Y)	2740.05	2658.7	2699.375	7067.18	9905.24	8486.21	2007.33	1816.93	1912.13	2994.66	1535.58	2265.12	1.652495	1.905533	0.244407
STIP1	LAYINPDLALEEK	Phospho(Y)	1639.79	1425.06	1532.425	1053.31	1079.76	1066.535	3670.04	3305.34	3487.69	791.92	1040.65	916.285	-0.52289	0.219063	-1.9284
STIP1	LDPHNHVLYSNR	Phospho(ST)	742.63	638.95	690.79	580.46	246.53	413.495	3466.1	3765.34	3615.72	773.73	240.16	506.945	-0.74038	-0.29396	-2.83438
SYAP1	DFGNYLFNFASAATK	Phospho(Y)	67.56	0	33.78	44.88	52.91	48.895	91.33	213.24	152.285	676.56	441.77	559.165	0.533518	-3.51552	1.8765
SYK	QESTVSFNPYEPELAPWAADKGPQR	Phospho(Y)	1819.23	3655.98	2737.605	3214.67	2730.9	2972.785	2213.62	1963.25	2088.435	3612.1	4071.65	3841.875	0.118901	-0.37	0.879388
TACSTD2	AAGDVDIGDAAYYFER	Phospho(Y)	857.93	953.8	905.865	898.15	1286.22	1092.185	1259.85	1860.52	1560.185	448.83	780.82	614.825	0.269849	0.82897	-1.34347
TENC1	GPLDGSPYAQVQRPPR	Phospho(Y)	23429.86	26218.83	24824.35	35446.96	50947.53	43197.25	9403.18	9587.69	9495.435	14324.96	15265.73	14795.35	0.799184	1.545796	0.639837
TFRC	SAFSNLFGGEPLSYTR	Phospho(Y)	7883.91	16793.4	12338.66	20104.22	20335.48	20219.85	30048.62	27584.29	28816.46	34474.46	42370.98	38422.72	0.712587	-0.92619	0.415067
TFRC	VSASPLLYTLIEK	Phospho(Y)	372.7	949.94	661.32	613.76	618.35	616.055	2856.95	3198.32	3027.635	1410.79	932.1	1171.445	-0.10229	-0.92716	-1.3699
TJP2	HPDIYAVPIK	Phospho(Y)	6325.21	7649.28	6987.245	29794.66	29504.12	29649.39	21168.06	23960.57	22564.32	10200.43	8932.03	9566.23	2.085207	1.63198	-1.23802
TJP2	IEIAQKHPDIYAVPIK	Phospho(Y)	2147.77	1647.44	1897.605	159.66	231.66	195.66	5638.39	5929.14	5783.765	931.64	874.17	902.905	-3.27776	-2.20623	-2.67936
TKT	NMAEQIIQEIYSQIQSK	Phospho(Y)	8.15	10.24	9.195	529.65	661.71	595.68	171.33	52.54	111.935	1707.6	1143.52	1425.56	6.017544	-1.25892	3.670796
TLN1	ALDYYMLR	Phospho(Y)	625.28	792.2	708.74	1083.05	1164.66	1123.855	1552.39	1602.99	1577.69	4094.7	6346.26	5220.48	0.665128	-2.21573	1.726369
TLN1	AVSSAIAQLLGEVAQGNENYAGIAAR	Phospho(Y)	258.41	1711.41	984.91	2110.55	3141.92	2626.235	1859.6	2151.85	2005.725	1420.79	1303.72	1362.255	1.414932	0.946999	-0.55813
TLN2	AVGSSMAQLLTCAAQGNEHYTGVAAR	Phospho(Y)	0	185.51	92.755	1674.21	1718.87	1696.54	35.01	0	17.505	828.03	640.47	734.25	4.193027	1.208252	5.390432
TLN2	ECDYSIDGINR	Phospho(Y)	1768.7	2376.22	2072.46	3051.5	3892.69	3472.095	946.46	658.86	802.66	918.91	924.12	921.515	0.744462	1.913727	0.199219
TM9SF1	AYQDMDIDK	Phospho(Y)	50.82	63.05	56.935	1463.58	1173.22	1318.4	32.35	95.06	63.705	98.62	13.99	56.305	4.533329	4.549381	-0.17814
TNK2	ALPQNDDHYVMQEHR	Phospho(Y)	336.02	231.45	283.735	58316.22	54862.89	56589.56	639.18	564.34	601.76	23483.05	14645.83	19064.44	7.639848	1.569652	4.985552
TNK2	KPTYDPVSEDQDPLSSDFK	Phospho(Y)	18051.27	22362.99	20207.13	54252.59	51946.41	53099.5	46934.06	54585.76	50759.91	49915.99	52395.46	51155.73	1.393834	0.053803	0.011206
TNK2	KVSSTHYYLLPERPSYLER	Phospho(Y)	11838.38	13712.09	12775.24	25606.73	29049.58	27328.16	29620.92	30615.09	30118.01	24391.01	22846.22	23618.62	1.097038	0.210464	-0.3507
TNS1	HVAYGGYSTPEDR	Phospho(Y)	2969.77	3342.98	3156.375	12591.7	7548.37	10070.04	4687.49	3735.99	4211.74	21270.67	15018.9	18144.79	1.673728	-0.84949	2.107067
TNS1	HPAGVYQVSGLHNK	Phospho(Y)	8152.87	11037.04	9594.955	19413.75	23584.06	21498.91	14572.04	13755.74	14163.89	28281.03	15843.53	22062.28	1.163915	-0.03732	0.639364
TNS3	LSLGQYDNDAGGQLPFSK	Phospho(Y)	20582.37	23794.46	22188.42	27014.82	27305.92	27160.37	11944.15	11047.03	11495.59	3895.59	4573.27	4234.43	0.291697	2.681263	-1.44084
TOM1L2	TVTYEDPQAVGGLASALDNR	Phospho(Y)	3245.67	2517.74	2881.705	6517.88	7916.54	7217.21	2534.65	2954.47	2744.56	2792.3	3550.12	3171.21	1.324519	1.186408	0.208459
TPX2	AQPVPHYGVPFKPQIPEAR	Phospho(Y)	65.63	0	32.815	1686.82	1508.14	1597.48	4612.93	4048.81	4330.87	3155.34	3323.35	3239.345	5.605299	-1.0199	-0.41895
TRIM25	FDTIYQILLK	Phospho(Y)	478.08	856.15	667.115	1350.84	1317.3	1334.07	1344.77	1077.27	1211.02	1949.29	2272.79	2111.04	0.999827	-0.66212	0.801731
TUBA3C	QLFHPEQLITGKEDAANNYAR	Phospho(Y)	67.68	278.11	172.895	170.69	0.23	85.46	86.79	1586.52	836.655	254.69	417.9	336.295	-1.01657	-1.97641	-1.31491
TUBA3C	IHFPLATYAPVISAEK	Phospho(Y)	6462.13	10587.88	8525.005	2782.59	2260.16	2521.375	14564.29	13658.63	14111.46	2666.14	2750.64	2708.39	-1.75749	-0.10322	-2.38136
TUBA3C	VGINYQPPTVVPGGDLAK	Phospho(Y)	6551.22	6481.69	6516.455	2217.74	4047.71	3132.725	9918.54	14006.13	11962.34	1597.39	1897.14	1747.265	-1.05667	0.84232	-2.77533
TUBA8	NLDIERPTYTNLNR	Phospho(Y)	2125.14	4175.16	3150.15	178.88	305.13	242.005	8070.98	6261.67	7166.325	311.34	420.66	366	-3.70231	-0.59681	-4.29132
TUBB	ISVYYNEATGGK	Phospho(Y)	1196.87	1181.06	1188.965	395.88	400.63	398.255	5978.35	5061.98	5520.165	870.83	499.97	685.4	-1.57794	-0.78325	-3.00969
TUBB	FWEVISDEHGIDPTGTYHGDSDLQLDR	Phospho(Y)	389.97	865.42	627.695	79.61	92.67	86.14	2040.35	1901.81	1971.08	47.24	73.87	60.555	-2.86531	0.508437	-5.0246
TUBB4B	INVYYNEATGGK	Phospho(Y)	1333.77	2414.03	1873.9	215.78	451	333.39	5236.88	4615.43	4926.155	398.11	210.98	304.545	-2.49076	0.130555	-4.01573
TUBB6	NSSYFVEWIPNNVK	Phospho(Y)	5723.35	7380.95	6552.15	4250.71	3925.26	4087.985	12319.5	10192.08	11255.79	3777.97	3897.37	3837.67	-0.68058	0.091159	-1.55236
TXNRD1	KVVYENAYGQFIGPHR	Phospho(Y)	738.14	803.96	771.05	976.47	735.37	855.92	2010.87	1401.32	1706.095	1286.19	999.97	1143.08	0.150652	-0.41738	-0.57777
TYK2	LLAQAEGEPCYIR	Phospho(Y)	28445.02	38485.01	33465.02	122407	136581.6	129494.3	46440.43	34158.29	40299.36	61599.43	47336.67	54468.05	1.952163	1.249406	0.434653
UBAP2L	FPLDYYSIPFPTPTTPLTGR	Phospho(Y)	226.31	1454.72	840.515	2160.85	3045.73	2603.29	6788.63	5973.9	6381.265	3372.55	2891.61	3132.08	1.630991	-0.26679	-1.02672
USP9Y	FNDYFEFPR	Phospho(Y)	485.04	790.37	637.705	1387.11	1217.02	1302.065	955.3	546.89	751.095	782.66	893.78	838.22	1.02984	0.635401	0.158334
VASP	VQIYHNPTANSFR	Phospho(Y)	4598.02	5340.91	4969.465	11543.95	8349.38	9946.665	11634.55	7930.01	9782.28	11892.74	10146.2	11019.47	1.001122	-0.14777	0.171812
VCL	NPGNQAAYEHFETMK	Phospho(Y)	161.73	203.25	182.49	34672.98	33468.75	34070.87	453.56	177.15	315.355	19375.25	24543.46	21959.36	7.544577	0.633703	6.121715
VCL	SFLDSGYR	Phospho(Y)	1508.38	1771.9	1640.14	7389.63	6192.92	6791.275	11630.78	6673.9	9152.34	3741.53	5815.9	4778.715	2.049863	0.50706	-0.93752
WASL	FYGPQVNNISHTK	Phospho(Y)	1433.23	1311.72	1372.475	4126.92	5677.63	4902.275	2221.9	2347.57	2284.735	4990.69	4162.08	4576.385	1.836672	0.099243	1.002182
WASL	VIYDFIEK	Phospho(Y)	31338.91	47944.91	39641.91	115469	106579.7	111024.4	75568.8	87117.41	81343.11	57203.32	58039.54	57621.43	1.485778	0.946199	-0.49741
WDR1	AHDGGIYAISWSPDSTHLLSASGDK	Phospho(Y)	835.1	1777.63	1306.365	1155.93	2093.46	1624.695	2230.89	2605.27	2418.08	1721.28	2558.79	2140.035	0.314611	-0.39747	-0.17623
YES1	GAYSLSIR	Phospho(Y)	578.53	778.63	678.58	3046.26	3283.75	3165.005	2413.55	1907.85	2160.7	1331.64	1660	1495.82	2.221617	1.081271	-0.53056
YWHAG	NLLSVAYK	Phospho(Y)	0	0	1	16.12	9.94	13.03	1345.22	1189.73	1267.475	6.62	1	3.31	3.703765	1.976934	-8.58091
ZDHHC5	LVPTGPTHREPSPVRYDNLSR	Phospho(ST)Phospho(Y)	13374.61	13598.04	13486.33	13343.87	14375.71	13859.79	18954.12	16419.51	17686.82	21144.24	21648.5	21396.37	0.039408	-0.62646	0.274692
