# Supplementary material for: Activation of IGF1R/p110β/AKT/mTOR confers resistance to α-specific PI3K inhibition
Source: Breast Cancer Res. 2016 Apr 5;18:41. doi: 10.1186/s13058-016-0697-1 (PMC4820873; doi:10.1186/s13058-016-0697-1)
Supplement: Additional file 3: — is a table presenting phosphopeptide intensity of MCF7p with insulin but without BYL719; and MCF7r with or without insulin treated for 24 hours with or without 5 μM BYL719. (PDF 308 kb) [file 13058_2016_697_MOESM3_ESM.pdf]

| Genes names | Sequence | Variablemodifications(positiondescription) | Sum(ve131151_MCF7_Resistante_BYL) | Sum(ve131152_MCF7_Resistante_BYL) | MCF7 Resistant -INS +BYL | Sum(ve131157_MCF7_Parent_INS) | Sum(ve131158_MCF7_Parent_INS) | MCF7 Parental +INS -BYL | Sum(ve131160_MCF7_Resistante_INS_BYL) | Sum(ve131161_MCF7_Resistante_INS_BYL) | MCF7 Resistant +INS +BYL | Sum(ve131163_MCF7_Resistante_INS) | Sum(ve131164_MCF7_Resistante_INS2) | MCF7 Resistant +INS -BYL | ratios Res +INS +BYL / Res -INS +BYL | ratios Res -BYL +INS / Res +BYL +INS | ratios Res / Par |
|---|---|---|---|---|---|---|---|---|---|---|---|---|---|---|---|---|---|
| ACLY | TTDGVYEGVAIGGDR | Phospho(Y) | 665.72 | 170.56 | 418.14 | 287.03 | 273.01 | 280.02 | 1187.28 | 929.65 | 1058.465 | 17.2 | 756.26 | 386.73 | 1.339916 | -1.45257 | 0.465797 |
| ACTB | LCYVALDFEQEMATAASSSSLEK | Phospho(Y) | 519.22 | 992.53 | 755.875 | 418.86 | 321.97 | 370.415 | 468.92 | 400.34 | 434.63 | 601.19 | 838.93 | 720.06 | -0.79836 | 0.728329 | 0.958975 |
| ACTG1 | GYSFTTTAER | Phospho(Y) | 33.82 | 95.18 | 64.5 | 447.12 | 679.28 | 563.2 | 76.86 | 284 | 180.43 | 308.41 | 275.11 | 291.76 | 1.484068 | 0.693343 | -0.94887 |
| ACTG1 | QEYDESGPSIVHR | Phospho(Y) | 0 | 135.83 | 67.915 | 242.21 | 574.34 | 408.275 | 22.2 | 0 | 11.1 | 219.79 | 0 | 109.895 | -2.61317 | 3.307494 | -1.89342 |
| ADAM9 | HVSPVTPPREVPIYANR | Phospho(ST)Phosp | 1179.31 | 2197.5 | 1688.405 | 325.64 | 338.04 | 331.84 | 1033.17 | 1644.42 | 1338.795 | 1615.06 | 1319.82 | 1467.44 | -0.33473 | 0.132366 | 2.144742 |
| AGFG1 | AGLQTADKYAALANLDNIFSAGQGGDQGSGFGTTGK | Phospho(Y) | 23.48 | 3498.17 | 1760.825 | 1905.3 | 28.65 | 966.975 | 1285.91 | 1730.86 | 1508.385 | 1678.47 | 2642.44 | 2160.455 | -0.22325 | 0.51833 | 1.159785 |
| AK1 | FNSYNNAGMPPFPIIHDEPTYAR | Phospho(Y) | 3875.25 | 6356.07 | 5115.66 | 2845.88 | 3135.75 | 2990.815 | 2917.22 | 4334.48 | 3625.85 | 1978.64 | 2891.09 | 2434.865 | -0.4966 | -0.57448 | -0.2967 |
| AK1 | VPIVINPNAYDNLAIYK | Phospho(Y) | 5540.88 | 8949.21 | 7245.045 | 5390.77 | 6673.74 | 6032.255 | 6421.43 | 10896.72 | 8659.075 | 3816.81 | 4213.23 | 4015.02 | 0.257218 | -1.10881 | -0.58729 |
| ALDOA | YTPSGQAGAAASESLFVSNHAY | Phospho(Y) | 3010.27 | 3807.95 | 3409.11 | 2413.59 | 3095.22 | 2754.405 | 4508.2 | 5217.45 | 4862.825 | 4774.22 | 7027.94 | 5901.08 | 0.5124 | 0.279184 | 1.099238 |
| ALDOA | VNPCIGGVILFHETLYQK | Phospho(Y) | 743.99 | 1.98 | 372.985 | 0 | 115.03 | 57.515 | 520.65 | 138.89 | 329.77 | 147.22 | 0.22 | 73.72 | -0.17766 | -2.16133 | 0.358118 |
| ANKS1A | IIASLADRPYEEPPQKPPR | Phospho(Y) | 3129.56 | 3292.51 | 3211.035 | 598.61 | 693.1 | 645.855 | 5460.28 | 4291.87 | 4876.075 | 2791.19 | 4640.6 | 3715.895 | 0.602682 | -0.39201 | 2.524428 |
| ANKS1A | EEDEHPYELLLTAETKK | Phospho(Y) | 846.28 | 1342.14 | 1094.21 | 0 | 184.58 | 92.29 | 85.66 | 85.26 | 85.46 | 10.45 | 1049.39 | 529.92 | -3.6785 | 2.632453 | 2.521528 |
| ANKS1A | EEDEHPYELLLTAETK | Phospho(Y) | 12847.77 | 16585.32 | 14716.55 | 5564.2 | 6246.88 | 5905.54 | 12345.29 | 17109.76 | 14727.53 | 8992.4 | 11252.46 | 10122.43 | 0.001076 | -0.54096 | 0.777415 |
| ANXA2P2 | AYTNFDAERDALNIETAIK | Phospho(Y) | 443.26 | 1435.78 | 939.52 | 135.69 | 1032.51 | 584.1 | 720.26 | 817.74 | 769 | 1940.62 | 2223.68 | 2082.15 | -0.28894 | 1.437019 | 1.833787 |
| ANXA2P2 | AYTNFDAER | Phospho(Y) | 2416.71 | 2781.85 | 2599.28 | 3936.1 | 5538.4 | 4737.25 | 1417.26 | 1869.56 | 1643.41 | 11538.3 | 6596.21 | 9067.255 | -0.66142 | 2.463973 | 0.936616 |
| AP2B1 | DSDYYNMLLK | Phospho(Y) | 434.66 | 731.79 | 583.225 | 552.66 | 521.67 | 537.165 | 1715.01 | 1693.92 | 1704.465 | 2343.24 | 2683.44 | 2513.34 | 1.547195 | 0.560287 | 2.226169 |
| ARAP1 | HYSVVLPTVSHSGFLYK | Phospho(Y) | 12.27 | 665.81 | 339.04 | 272.37 | 186.01 | 229.19 | 0 | 344.64 | 172.32 | 9.24 | 452.38 | 230.81 | -0.97637 | 0.421616 | 0.010162 |
| ARFGAP2 | LAYQELQIDR | Phospho(Y) | 300.17 | 346.24 | 323.205 | 121.78 | 123.15 | 122.465 | 364.44 | 510.14 | 437.29 | 34.05 | 574.78 | 304.415 | 0.436141 | -0.52255 | 1.31367 |
| ARHGAP12 | ATTPPNQGRPDSPVYANLQELK | Phospho(ST)Phosp | 5405.87 | 8531.07 | 6968.47 | 1928.08 | 3653.39 | 2790.735 | 3413.41 | 5817.34 | 4615.375 | 7081.45 | 16178.1 | 11629.78 | -0.59439 | 1.333303 | 2.059106 |
| ARHGAP33 | RPTPPEPLYVNLALGPR | Phospho(Y) | 26.83 | 680.07 | 353.45 | 425.03 | 476.63 | 450.83 | 1 | 0 | 0.5 | 59.99 | 10.51 | 35.25 | -9.46536 | 6.139551 | -3.67689 |
| ARHGAP35 | NEEENIYSVPHDSTQGK | Phospho(Y) | 222704.8 | 205859.1 | 214282 | 83777.02 | 126772.6 | 105274.8 | 174797.8 | 182086.5 | 178442.1 | 282429.1 | 271220.7 | 276824.9 | -0.26405 | 0.633517 | 1.394813 |
| ARPC3 | DTDIVDEAIYYFK | Phospho(Y) | 1536.21 | 1873.22 | 1704.715 | 934.04 | 913.8 | 923.92 | 2164.12 | 4833 | 3498.56 | 2268.97 | 3955.72 | 3112.345 | 1.037231 | -0.16876 | 1.752162 |
| ARRB2 | VDPVDGVVLVDPDYLK | Phospho(Y) | 120.92 | 1047.01 | 583.965 | 46.28 | 272.23 | 159.255 | 216.1 | 1107.13 | 661.615 | 239.22 | 574.3 | 406.76 | 0.18011 | -0.70181 | 1.352839 |
| ARRB2 | VDPVDGVVLVDPDYLKDR | Phospho(Y) | 1647.14 | 2143.17 | 1895.155 | 1594.73 | 1304.23 | 1449.48 | 2269.31 | 2968.77 | 2619.04 | 2137.26 | 2059.71 | 2098.485 | 0.466722 | -0.31969 | 0.533813 |
| ATP11C | IYGVAVYTGMETK | Phospho(Y) | 804.22 | 813.79 | 809.005 | 908.66 | 1025.62 | 967.14 | 2410.74 | 2326.14 | 2368.44 | 1752.63 | 2123.44 | 1938.035 | 1.549717 | -0.28934 | 1.002798 |
| ATP11C | INIYSNSLEAVAR | Phospho(Y) | 894.38 | 1039.63 | 967.005 | 1144.77 | 1216.93 | 1180.85 | 1953.01 | 2139.14 | 2046.075 | 1430.46 | 1416.72 | 1423.59 | 1.081264 | -0.52333 | 0.269708 |
| ATP13A1 | ATTGLKPVDSGCVAYVLR | Phospho(Y) | 21.94 | 636.05 | 328.995 | 258.12 | 366.74 | 312.43 | 21.63 | 36.69 | 29.16 | 84.78 | 0 | 42.39 | -3.496 | 0.539733 | -2.88174 |
| ATP1A1 | GIVVYTGDR | Phospho(Y) | 173688.7 | 202690.1 | 188189.4 | 47454.62 | 56539 | 51996.81 | 89545.7 | 107668.1 | 98606.92 | 179565.1 | 139535.2 | 159550.1 | -0.93242 | 0.694249 | 1.617515 |
| ATP1A1 | EQPLDEELKDAFQNAYLELGGLGER | Phospho(Y) | 1456.71 | 2510.15 | 1983.43 | 1048.39 | 1232.67 | 1140.53 | 3492.53 | 5388.63 | 4440.58 | 720.57 | 1582.59 | 1151.58 | 1.162751 | -1.94713 | 0.01391 |
| ATP2B1 | IQESYGDVYGICTK | Phospho(Y) | 687.94 | 644.87 | 666.405 | 339.55 | 308.87 | 324.21 | 1555.96 | 1215.06 | 1385.51 | 934.81 | 657.67 | 796.24 | 1.055946 | -0.79914 | 1.296275 |
| BAIAP2 | LSDSYSNTLPVR | Phospho(Y) | 37.67 | 220.82 | 129.245 | 446.4 | 335.98 | 391.19 | 263.87 | 0 | 131.935 | 0 | 608.66 | 304.33 | 0.029719 | 1.205809 | -0.36223 |
| BCAR1 | AQQGLYQVPGPSPQFQSPPAK | Phospho(Y) | 2190.64 | 3229.16 | 2709.9 | 366.2 | 335.77 | 350.985 | 2148.01 | 1856.01 | 2002.01 | 2918.22 | 3156.57 | 3037.395 | -0.43679 | 0.601385 | 3.113353 |
| BCAR1 | EETYDVPPAFAK | Phospho(Y) | 4238.28 | 4212.42 | 4225.35 | 1108.26 | 1435.41 | 1271.835 | 4086.18 | 5619.16 | 4852.67 | 6024 | 6520.83 | 6272.415 | 0.199708 | 0.370242 | 2.302109 |
| BCAR1 | AQQGLYQVPGPSPQFQSPPAK | Phospho(Y) | 20692.45 | 26986.43 | 23839.44 | 7528.98 | 7516.71 | 7522.845 | 22156.53 | 21352.65 | 21754.59 | 33990.07 | 34695.04 | 34342.56 | -0.13203 | 0.658678 | 2.190647 |
| BCAR1 | GLPPSNHHAVYDVPPSVSK | Phospho(Y) | 14043.81 | 19414.9 | 16729.36 | 5530.42 | 5991.7 | 5761.06 | 18666.78 | 26220.95 | 22443.87 | 25523.4 | 25750.94 | 25637.17 | 0.423939 | 0.191916 | 2.153831 |
| BCAR1 | HLLAPGPQDIYDVPPVR | Phospho(Y) | 39667.68 | 47182.19 | 43424.94 | 7240.05 | 7476.23 | 7358.14 | 29284.69 | 37466.71 | 33375.7 | 26658.99 | 32933.28 | 29796.14 | -0.37973 | -0.16367 | 2.017712 |
| BCAR1 | RPGPGTLYDVPR | Phospho(Y) | 10678.95 | 14149.86 | 12414.41 | 3120.24 | 3240.85 | 3180.545 | 12530.49 | 15599.39 | 14064.94 | 9927.68 | 12494.34 | 11211.01 | 0.180088 | -0.32719 | 1.81757 |
| BCAR1 | VLPPEVADGGVVDSGVYAVPPPAER | Phospho(Y) | 44114.44 | 46068.61 | 45091.53 | 14340.99 | 14313.07 | 14327.03 | 32272.99 | 39504 | 35888.5 | 37106.34 | 37585.66 | 37346 | -0.32933 | 0.057432 | 1.382214 |
| BCAR1 | DVPDGPLLREETYDVPPAFAK | Phospho(Y) | 35673.17 | 49147.1 | 42410.14 | 13710.45 | 13287.05 | 13498.75 | 24692.9 | 35002.47 | 29847.69 | 31958.04 | 34444.55 | 33201.3 | -0.50679 | 0.15362 | 1.298414 |
| BCR | GHGQPGADAEKPFYVNVEFHHER | Phospho(Y) | 7989.32 | 9658.33 | 8823.825 | 4270.13 | 3821.48 | 4045.805 | 4354.1 | 7887.02 | 6120.56 | 2235.2 | 760.96 | 1498.08 | -0.52774 | -2.03055 | -1.43331 |
| BCR | KGHGQPGADAEKPFYVNVEFHHER | Phospho(Y) | 0 | 0.04 | 0.02 | 11.88 | 25.39 | 18.635 | 0 | 187.62 | 93.81 | 0 | 1 | 0.5 | 12.19553 | -7.55167 | -5.21994 |
| BICD1 | YENEKAMVTETMTKLR | Phospho(Y) | 61.38 | 172.56 | 116.97 | 757.16 | 853.12 | 805.14 | 78.03 | 123.19 | 100.61 | 45.79 | 407.19 | 226.49 | -0.21736 | 1.170674 | -1.82979 |
| C11orf52 | SPGLMSEDSNLHYADIQVCSRPHAR | Phospho(Y) | 41126.31 | 47715.71 | 44421.01 | 33353.65 | 38723.05 | 36038.35 | 28592.25 | 55477.48 | 42034.87 | 53696.19 | 57866.9 | 55781.55 | -0.07966 | 0.408201 | 0.630255 |
| C11orf52 | HVHLENATEYATLR | Phospho(Y) | 16915.49 | 28227.15 | 22571.32 | 32115.55 | 36506.02 | 34310.79 | 44447.91 | 51464.1 | 47956.01 | 40897.03 | 39108.1 | 40002.57 | 1.087221 | -0.26162 | 0.22143 |
| C9orf169 | NPYAHISIPR | Phospho(Y) | 612.03 | 1162.1 | 887.065 | 1120.03 | 1022.07 | 1071.05 | 2198.44 | 2261.61 | 2230.025 | 3924.53 | 4436.71 | 4180.62 | 1.329948 | 0.906657 | 1.964691 |
| C9orf78 | NAEDCLYELPENIR | Phospho(Y) | 40294.89 | 45276.68 | 42785.79 | 4848.83 | 5249.94 | 5049.385 | 24154.32 | 37723.02 | 30938.67 | 30784.5 | 35962.74 | 33373.62 | -0.46772 | 0.109297 | 2.724529 |
| CALCR | AAAAAAEAGDIPIYICHQEPR | Phospho(Y) | 0 | 1 | 0.5 | 189.08 | 0 | 94.54 | 15.15 | 0 | 7.575 | 0 | 5.5 | 2.75 | 3.921246 | -1.46181 | -5.10342 |
| CALCR | AAAAAAEAGDIPIYICHQELR | Phospho(Y) | 100.29 | 309.76 | 205.025 | 678.5 | 659.86 | 669.18 | 0.81 | 58 | 29.405 | 36.81 | 1.11 | 18.96 | -2.80167 | -0.6331 | -5.14136 |
| CARKD | IGVVGGCQEYTGAPYFAAISALK | Phospho(Y) | 20178.11 | 27589.24 | 23883.68 | 6928 | 8843.28 | 7885.64 | 13615.69 | 20403.77 | 17009.73 | 16731.8 | 19487.6 | 18109.7 | -0.48966 | 0.090402 | 1.199463 |
| CASKIN2 | TPQPPAEEPPHPLTYSQLPR | Phospho(Y) | 1506.88 | 1339.15 | 1423.015 | 1558.69 | 1149.89 | 1354.29 | 2611.67 | 3463.7 | 3037.685 | 2771.02 | 2896.08 | 2833.55 | 1.094021 | -0.10036 | 1.065074 |
| CASKIN2 | NTYNQTALDIVNQFTTSQASR | Phospho(Y) | 21795.38 | 26643.3 | 24219.34 | 26112.95 | 26355.28 | 26234.12 | 35355.19 | 42590.46 | 38972.83 | 38598.39 | 36902.61 | 37750.5 | 0.686309 | -0.04597 | 0.525052 |
| CDC42BPB | NKPYISWPSSGGSEPSVTVPLR | Phospho(Y) | 501.92 | 120.53 | 311.225 | 45.07 | 18.68 | 31.875 | 740.19 | 973.11 | 856.65 | 148.34 | 916.27 | 532.305 | 1.460748 | -0.68645 | 4.061756 |
| CDK16 | LGEGTYATVYK | Phospho(Y) | 72146.59 | 63853.76 | 68000.18 | 36621.07 | 31653.41 | 34137.24 | 62942.63 | 72946.87 | 67944.75 | 89840.95 | 71014.86 | 80427.91 | -0.00118 | 0.243334 | 1.23635 |
| CDK2 | IGEGTYGVVYK | Phospho(Y) | 1879593 | 2038598 | 1959096 | 1261332 | 1151999 | 1206665 | 1711212 | 1979933 | 1845572 | 2982033 | 2365246 | 2673640 | -0.08612 | 0.534737 | 1.14778 |
| CDK3 | IGEGTYGVVYK | Phospho(Y) | 435.92 | 445.42 | 440.67 | 299.16 | 278.14 | 288.65 | 354.43 | 344.37 | 349.4 | 577.13 | 415.73 | 496.43 | -0.33482 | 0.506711 | 0.782269 |
| CDK5 | IGEGTYGTVFK | Phospho(Y) | 4483.91 | 3916.84 | 4200.375 | 3046.38 | 2814.34 | 2930.36 | 3950.22 | 4553.11 | 4251.665 | 5345.42 | 4017.57 | 4681.495 | 0.01751 | 0.138941 | 0.675891 |
| CDKL5 | NLSEGNNANYTEYVATR | Phospho(Y) | 1077.82 | 1260.03 | 1168.925 | 908.91 | 919.18 | 914.045 | 1276.59 | 1004.04 | 1140.315 | 56.07 | 1125.23 | 590.65 | -0.03575 | -0.94906 | -0.62996 |
| CDV3 | LQLDNQYAVLENQK | Phospho(Y) | 2807.93 | 3982.09 | 3395.01 | 1262.83 | 1200.56 | 1231.695 | 6885.03 | 6570.61 | 6727.82 | 8197.71 | 9504.23 | 8850.97 | 0.986723 | 0.395696 | 2.845191 |
| CDV3 | KTPQGPPEIYSDTQFPSLQSTAK | Phospho(Y) | 42963.53 | 46936.03 | 44949.78 | 12625.59 | 15016.07 | 13820.83 | 62733.15 | 80821.32 | 71777.24 | 68882.3 | 84422.93 | 76652.62 | 0.675212 | 0.094809 | 2.471491 |
| CELSR2 | SQPSYIPFLLR | Phospho(Y) | 895.48 | 1104.67 | 1000.075 | 42.97 | 48.97 | 45.97 | 830.33 | 1245.66 | 1037.995 | 221.86 | 446.1 | 333.98 | 0.053691 | -1.63597 | 2.860997 |
| CEP170 | EPSYFEIPTK | Phospho(Y) | 393.07 | 492.64 | 442.855 | 360.18 | 269.34 | 314.76 | 407.33 | 868.9 | 638.115 | 1166.4 | 1325.48 | 1245.94 | 0.526982 | 0.965346 | 1.98491 |
| CFL1 | YALYDATYETKESK | Phospho(Y) | 1708.92 | 1993.96 | 1851.44 | 126.89 | 111.72 | 119.305 | 1025.39 | 423.75 | 724.57 | 1590.91 | 1219.5 | 1405.205 | -1.35345 | 0.955584 | 3.558054 |
| CFL1 | YALYDATYETK | Phospho(Y) | 15027.33 | 16244.6 | 15635.97 | 3256.33 | 3260.21 | 3258.27 | 20676.44 | 20816.92 | 20746.68 | 17936.56 | 20682.45 | 19309.51 | 0.408012 | -0.10357 | 2.567133 |
| CFL1 | HELQANCYEEVKDR | Phospho(Y) | 58265.92 | 64119.6 | 61192.76 | 13391.29 | 20669.33 | 17030.31 | 40647.36 | 42448.89 | 41548.13 | 46127.3 | 21469.54 | 33798.42 | -0.55858 | -0.29783 | 0.988851 |
| CFL1 | NIILEEGKEILVGDVGQTVDDPYATFVK | Phospho(Y) | 1522.45 | 2141.72 | 1832.085 | 1049.19 | 1139.4 | 1094.295 | 1548.03 | 2163.3 | 1855.665 | 321.5 | 834.32 | 577.91 | 0.01845 | -1.68302 | -0.92108 |
| CLDN3 | STGPGASLGTGYDR | Phospho(Y) | 12365.33 | 13268 | 12816.67 | 7954.64 | 8911.99 | 8433.315 | 12887.42 | 13005.85 | 12946.64 | 16866.15 | 13654.77 | 15260.46 | 0.014556 | 0.237221 | 0.855627 |
| CLDN3 | STGPGASLGTGYDRK | Phospho(Y) | 2203.34 | 3461.55 | 2832.445 | 1740.66 | 2072.07 | 1906.365 | 2644.52 | 3351.09 | 2997.805 | 2966.17 | 2814.1 | 2890.135 | 0.081859 | -0.05277 | 0.600313 |
| CLTC | ALEHFTDLYDIKR | Phospho(Y) | 1759.24 | 2015.89 | 1887.565 | 398.02 | 376.69 | 387.355 | 1090.52 | 2236.45 | 1663.485 | 1142.54 | 1885.13 | 1513.835 | -0.18232 | -0.136 | 1.96648 |
| CMTM6 | MENGAVYSPTTEEDPGPAR | Phospho(Y) | 1905.13 | 952.57 | 1428.85 | 505.99 | 622.1 | 564.045 | 6633.75 | 2005.09 | 4319.42 | 2703.99 | 4350.23 | 3527.11 | 1.595983 | -0.29235 | 2.644604 |
| CPNE3 | LYGPTNFSPIINHVAR | Phospho(Y) | 349.97 | 466.36 | 408.165 | 470.69 | 509.56 | 490.125 | 305.93 | 337.21 | 321.57 | 690.33 | 477.51 | 583.92 | -0.34402 | 0.860638 | 0.252621 |
| CREBBP | AHLLAEKIYK | Phospho(Y) | 3288.65 | 3374.02 | 3331.335 | 2024.34 | 2153.56 | 2088.95 | 2557.03 | 3080.8 | 2818.915 | 3327.65 | 3714.91 | 3521.28 | -0.24096 | 0.32096 | 0.753322 |
| CRIP2 | GVNIGGAGSYIYEKPLAEGPQVTGPIEVPAAR | Phospho(Y) | 13252.51 | 17084.66 | 15168.59 | 11943.45 | 11860.58 | 11902.02 | 13382.38 | 15024.25 | 14203.32 | 14556.7 | 15118.04 | 14837.37 | -0.09486 | 0.063008 | 0.31803 |
| CRK | YRPASASVSALIGGNQEGSHPQPLGGPEPGPYAQPSVN | Phospho(Y) | 207151.6 | 304261.5 | 255706.5 | 181623.8 | 176985.2 | 179304.5 | 182692.5 | 254784.6 | 218738.6 | 207735.6 | 235785 | 221760.3 | -0.22528 | 0.019793 | 0.306589 |
| CRKL | NSNSYGIPEPAHAYAQPQTTTPLPAVSGSPGAAITPLPST | Phospho(Y) | 257259.4 | 329987 | 293623.2 | 243909.5 | 252747.4 | 248328.5 | 318405.8 | 423055.7 | 370730.7 | 346706 | 343086 | 344896 | 0.336406 | -0.10421 | 0.473912 |
| CSNK2B | IHPMAYQLQLQAASNFKSPVK | Phospho(Y) | 172.31 | 304.83 | 238.57 | 0 | 705.61 | 352.805 | 54.69 | 229.02 | 141.855 | 25.75 | 142.6 | 84.175 | -0.75 | -0.75295 | -2.06741 |
| CTNNA1 | QLQQAVTGISNAAQATASDDASQHQGGGGGELAYALNN | Phospho(Y) | 1531.19 | 1807.88 | 1669.535 | 478.31 | 937.09 | 707.7 | 1712.6 | 0 | 856.3 | 548.99 | 1444.91 | 996.95 | -0.96326 | 0.219405 | 0.494383 |
| CTNNA1 | NAGNEQDLGIQYK | Phospho(Y) | 147.13 | 393.45 | 270.29 | 340.98 | 432.23 | 386.605 | 20.78 | 17.96 | 19.37 | 446.52 | 80.23 | 263.375 | -3.80261 | 3.765223 | -0.55374 |
| CTNNB1 | AAVSHWQQQSYLDSGIHSGATTTAPSLSGK | Phospho(Y) | 709.31 | 918.33 | 813.82 | 17.36 | 527.94 | 272.65 | 1367.05 | 9.5 | 688.275 | 13.49 | 844.75 | 429.12 | -0.24172 | -0.6816 | 0.654331 |
| CTNND1 | LNGPQDHSHLLYSTIPR | Phospho(Y) | 1018.87 | 1380.92 | 1199.895 | 708.91 | 769.14 | 739.025 | 760.53 | 1487.92 | 1124.225 | 1961.57 | 1541.21 | 1751.39 | -0.09398 | 0.63957 | 1.244805 |
| CTNND1 | QDVYGPQPQVR | Phospho(Y) | 2162.1 | 2270.95 | 2216.525 | 1105.86 | 1234.36 | 1170.11 | 1866.28 | 1417.44 | 1641.86 | 2694.8 | 2755.5 | 2725.15 | -0.43297 | 0.731005 | 1.219691 |
| CTNND1 | SLDNNYSTPNER | Phospho(Y) | 110.85 | 161.61 | 136.23 | 757.33 | 766.15 | 761.74 | 51.86 | 83.5 | 67.68 | 854.79 | 597.1 | 725.945 | -1.00924 | 3.423059 | -0.06944 |
| CTTN | LPSSPVYEDAASFK | Phospho(ST)Phosp | 7941.59 | 11399.13 | 9670.36 | 2790.87 | 3665.34 | 3228.105 | 11414.73 | 21564.36 | 16489.55 | 16384.29 | 31779.83 | 24082.06 | 0.76991 | 0.546407 | 2.899199 |
| CTTN | GPVSGTEPEPVYSMEAADYR | Phospho(Y) | 23602.97 | 24152.43 | 23877.7 | 5711.49 | 6603.42 | 6157.455 | 20238.1 | 23136.16 | 21687.13 | 40392.97 | 46349.29 | 43371.13 | -0.13882 | 0.999896 | 2.816329 |
| CTTN | LPSSPVYEDAASFK | Phospho(Y) | 66266.84 | 73998.79 | 70132.82 | 28619.84 | 31039.73 | 29829.79 | 82100.49 | 146165.1 | 114132.8 | 87527.56 | 117431.4 | 102479.5 | 0.702552 | -0.15538 | 1.780509 |
| CTTN | NASTFEDVTQVSSAYQK | Phospho(Y) | 2498.7 | 2549.63 | 2524.165 | 1272.88 | 1463.53 | 1368.205 | 2335.87 | 3882.79 | 3109.33 | 3378.23 | 2935.41 | 3156.82 | 0.300798 | 0.021868 | 1.206188 |
| CTTN | VDQSAVGFEYQGK | Phospho(Y) | 1697.72 | 1764.65 | 1731.185 | 1147.99 | 1605.56 | 1376.775 | 1898.54 | 1667.68 | 1783.11 | 2523.15 | 2126.7 | 2324.925 | 0.042636 | 0.382788 | 0.755891 |
| DAB2IP | AGQTPTTPGTSEGAPGRPQLLAPLSFQNPVYQMAAGLP | Phospho(Y) | 6426.29 | 12789.08 | 9607.685 | 8584.04 | 9053.89 | 8818.965 | 9923.69 | 9992.12 | 9957.905 | 8375.63 | 8704.89 | 8540.26 | 0.051653 | -0.22156 | -0.04633 |
| DBNL | FQDVGPQAPVGSVYQK | Phospho(Y) | 4298.91 | 4028.07 | 4163.49 | 1460.1 | 1621.09 | 1540.595 | 6061.71 | 6104.13 | 6082.92 | 10491.3 | 8653.54 | 9572.42 | 0.546971 | 0.65412 | 2.635396 |
| DCBLD2 | AGKPGLPAPDELVYQVPQSTQEVSGAGR | Phospho(Y) | 17987.44 | 19846.75 | 18917.1 | 5308.56 | 5498.44 | 5403.5 | 12087.96 | 15646.82 | 13867.39 | 18032.73 | 19743.8 | 18888.27 | -0.44799 | 0.445794 | 1.805524 |
| DDR1 | EPPPYQEPRPR | Phospho(Y) | 16202.19 | 19889.39 | 18045.79 | 2226.61 | 2412.73 | 2319.67 | 2764.98 | 2534.67 | 2649.825 | 5744.52 | 4764.87 | 5254.695 | -2.76769 | 0.98771 | 1.179687 |
| DDX3Y | ELAVQIYEEAR | Phospho(Y) | 867.15 | 577.81 | 722.48 | 0 | 76.81 | 38.405 | 894.76 | 89.72 | 492.24 | 15.28 | 269.59 | 142.435 | -0.5536 | -1.78906 | 1.890938 |
| DDX6 | FGHLGLAINLITYDDRFNLK | Phospho(Y) | 1007.85 | 1884.56 | 1446.205 | 292.64 | 202.48 | 247.56 | 672.3 | 1594.32 | 1133.31 | 141.09 | 461.19 | 301.14 | -0.35173 | -1.91204 | 0.282656 |
| DKFZp779K1237 | ERVPFILTYDFIHVIQQGK | Phospho(Y) | 1436.84 | 1297.5 | 1367.17 | 200.71 | 138.79 | 169.75 | 299.87 | 1077.1 | 688.485 | 329.88 | 479.34 | 404.61 | -0.9897 | -0.76689 | 1.25312 |
| DKFZp779K1237 | VPFILTYDFIHVIQQGK | Phospho(Y) | 10222.33 | 12923.7 | 11573.02 | 8114.47 | 5525 | 6819.735 | 19034.65 | 25687.8 | 22361.23 | 11697.26 | 13288.4 | 12492.83 | 0.950234 | -0.8399 | 0.873313 |
| DLG3 | RDNEVDGQDYHFVVSR | Phospho(Y) | 23195.76 | 31179.87 | 27187.82 | 23373.94 | 22204.69 | 22789.32 | 70559.09 | 95529.17 | 83044.13 | 46402.33 | 56729.46 | 51565.9 | 1.610918 | -0.68746 | 1.17806 |
| DLG3 | DNEVDGQDYHFVVSR | Phospho(Y) | 6329.6 | 7185.92 | 6757.76 | 9733.09 | 13525.22 | 11629.16 | 14815.17 | 20841.63 | 17828.4 | 19694.83 | 23635.18 | 21665.01 | 1.39956 | 0.281189 | 0.89762 |
| DNAJA1 | NVVHQLSVTLEDLYNGATR | Phospho(Y) | 438.76 | 881.43 | 660.095 | 258.11 | 260.16 | 259.135 | 453.62 | 679.24 | 566.43 | 337.01 | 165.24 | 251.125 | -0.22078 | -1.17349 | -0.0453 |
| DNMT1 | LSIFDANESGFESYEALPQHK | Phospho(Y) | 1297.92 | 2047.08 | 1672.5 | 86.4 | 246.46 | 166.43 | 1700.45 | 2163.2 | 1931.825 | 1509.67 | 1827.62 | 1668.645 | 0.207958 | -0.21129 | 3.32569 |
| DSTN | YALYDASFETK | Phospho(Y) | 270.73 | 413.79 | 342.26 | 23.15 | 237.19 | 130.17 | 321.67 | 607.37 | 464.52 | 51.62 | 119.47 | 85.545 | 0.440648 | -2.44099 | -0.60564 |
| DYRK1B | IYQYIQSR | Phospho(Y) | 692434.2 | 682181.6 | 687307.9 | 246528.4 | 262936.9 | 254732.7 | 313303.4 | 328458.9 | 320881.1 | 528441.5 | 623253.4 | 575847.5 | -1.09892 | 0.843648 | 1.176703 |
| DYRK4 | VYTYIQSR | Phospho(Y) | 2518.25 | 3027.44 | 2772.845 | 3008.91 | 4136.39 | 3572.65 | 692.35 | 637.22 | 664.785 | 3060.18 | 3153.06 | 3106.62 | -2.06041 | 2.224386 | -0.20165 |
| EEF1A2 | STTTGHLIYK | Phospho(Y) | 8242.62 | 10962.11 | 9602.365 | 1719.66 | 1931.45 | 1825.555 | 603.77 | 521.02 | 562.395 | 8503.69 | 8344.92 | 8424.305 | -4.09373 | 3.904902 | 2.206223 |
| EEF1A2 | EHALLAYTLGVK | Phospho(Y) | 33001.18 | 37831.73 | 35416.46 | 21701.5 | 23299.47 | 22500.49 | 24936.86 | 32177.56 | 28557.21 | 39747.43 | 42099.03 | 40923.23 | -0.31056 | 0.519065 | 0.862964 |
| EEF2 | KEDLYLKPIQR | Phospho(Y) | 738.26 | 1211.97 | 975.115 | 775.59 | 665.45 | 720.52 | 419.74 | 916.93 | 668.335 | 65.33 | 1420.75 | 743.04 | -0.545 | 0.152868 | 0.044401 |
| EGFR | RPAGSVQNPVYHNQPLNPAPSR | Phospho(Y) | 1661.63 | 3261.76 | 2461.695 | 3202.3 | 3059.02 | 3130.66 | 5517.39 | 6702.57 | 6109.98 | 3167.77 | 6915.28 | 5041.525 | 1.311516 | -0.27731 | 0.687393 |
| EGFR | GSHQISLDNPDYQQDFFPK | Phospho(Y) | 320.73 | 913.51 | 617.12 | 1078.76 | 1019 | 1048.88 | 216.93 | 733.45 | 475.19 | 250.22 | 1151.72 | 700.97 | -0.37705 | 0.560848 | -0.58143 |
| EIF5A | NDFQLIGIQDGYLSLLQDSGEVR | Phospho(Y) | 3446.28 | 4630.1 | 4038.19 | 883.11 | 1731.77 | 1307.44 | 2418.21 | 87.3 | 1252.755 | 1577.52 | 2340.38 | 1958.95 | -1.6886 | 0.644976 | 0.583336 |
| ELAVL1 | NVALLSQLYHSPAR | Phospho(Y) | 1793.85 | 2410.5 | 2102.175 | 1021.2 | 999.14 | 1010.17 | 1915.65 | 2020.63 | 1968.14 | 2901.45 | 2608.7 | 2755.075 | -0.09505 | 0.485259 | 1.447493 |
| ELP3 | NLHDALSGHTSNNIYEAVK | Phospho(Y) | 369.33 | 865.96 | 617.645 | 60.37 | 24.44 | 42.405 | 23.56 | 444.13 | 233.845 | 379.18 | 580.55 | 479.865 | -1.40123 | 1.037076 | 3.500322 |
| ENO3 | AAVPSGASTGIYEALELR | Phospho(Y) | 7079.9 | 8591.97 | 7835.935 | 5461.2 | 5826.68 | 5643.94 | 6986.39 | 9943.27 | 8464.83 | 6201.88 | 7534.85 | 6868.365 | 0.111376 | -0.30151 | 0.283264 |
| EPHA1 | LLDDFDGTYETQGGKIPIR | Phospho(Y) | 2417.1 | 3312.12 | 2864.61 | 14.79 | 2.05 | 8.42 | 400.11 | 1068.02 | 734.065 | 69.3 | 298.27 | 183.785 | -1.96436 | -1.99789 | 4.448055 |
| EPHA1 | LLDDFDGTYETQGGK | Phospho(Y) | 9507.59 | 11250.76 | 10379.18 | 422.36 | 673.44 | 547.9 | 6686.78 | 9693.19 | 8189.985 | 4236.95 | 3445.25 | 3841.1 | -0.34176 | -1.09234 | 2.809535 |
| EPHA1 | LWLKPYVDLQAYEDPAQGALDFTR | Phospho(Y) | 1683.85 | 3519.77 | 2601.81 | 188.16 | 238.84 | 213.5 | 1591.02 | 2501.17 | 2046.095 | 490.83 | 482.85 | 486.84 | -0.34664 | -2.07135 | 1.189212 |
| EPHA2 | TYVDPHTYEDPNQAVLK | Phospho(ST)Phosp | 7175.36 | 10497.11 | 8836.235 | 1362.26 | 2100.35 | 1731.305 | 4254.74 | 5795.88 | 5025.31 | 7006.54 | 13756.42 | 10381.48 | -0.81422 | 1.046728 | 2.58408 |
| EPHA2 | VLEDDPEATYTTSGGK | Phospho(Y) | 16615.69 | 20116.36 | 18366.03 | 5675.73 | 6327.43 | 6001.58 | 17258.7 | 20648.17 | 18953.44 | 25696.46 | 21042.57 | 23369.52 | 0.04542 | 0.302168 | 1.961214 |
| EPHA2 | TYVDPHTYEDPNQAVLK | Phospho(Y) | 1279.16 | 1697.04 | 1488.1 | 567.76 | 599.28 | 583.52 | 1302.66 | 1820.4 | 1561.53 | 1966.72 | 2181.15 | 2073.935 | 0.069489 | 0.40941 | 1.829517 |
| EPHA4 | VLEDDPEAAYTTR | Phospho(Y) | 4201.45 | 5319.74 | 4760.595 | 2978.34 | 3516.08 | 3247.21 | 5261.63 | 5171.88 | 5216.755 | 4258.53 | 4131.83 | 4195.18 | 0.132011 | -0.31442 | 0.369532 |
| EPHA4 | TYVDPFTYEDPNQAVR | Phospho(Y) | 2073.5 | 2832.34 | 2452.92 | 3064.66 | 3024.64 | 3044.65 | 2487.59 | 3401.82 | 2944.705 | 4090.26 | 1990.63 | 3040.445 | 0.263623 | 0.046159 | -0.00199 |
| EPHA7 | ADQEGDEELYFHFK | Phospho(Y) | 966.52 | 922.07 | 944.295 | 7400.05 | 9312.71 | 8356.38 | 1851.58 | 2218.77 | 2035.175 | 1997.13 | 2602.78 | 2299.955 | 1.107843 | 0.176453 | -1.86127 |
| EPHA7 | VIEDDPEAVYTTTGGK | Phospho(Y) | 959.17 | 1576.1 | 1267.635 | 4050.96 | 3302.94 | 3676.95 | 1216.9 | 1689.52 | 1453.21 | 967.05 | 835.27 | 901.16 | 0.197104 | -0.68939 | -2.02865 |
| EPHB3 | FLEDDPSDPTYTSSLGGK | Phospho(Y) | 2192.33 | 2083.81 | 2138.07 | 981.64 | 942.05 | 961.845 | 3509.73 | 4444.53 | 3977.13 | 2993.16 | 3516.29 | 3254.725 | 0.895419 | -0.28919 | 1.758659 |
| EPHB4 | EIDVSYVK | Phospho(Y) | 997.64 | 1660.56 | 1329.1 | 199.72 | 206.16 | 202.94 | 84.37 | 24.05 | 54.21 | 681.85 | 645.52 | 663.685 | -4.61575 | 3.613868 | 1.709445 |
| EPHB4 | VYIDPFTYEDPNEAVR | Phospho(Y)Phosph | 1809.45 | 5754.48 | 3781.965 | 161.16 | 460.83 | 310.995 | 548.32 | 980.2 | 764.26 | 210.99 | 1590.02 | 900.505 | -2.307 | 0.236671 | 1.533843 |
| EPHB4 | FLEENSSDPTYTSSLGGK | Phospho(Y) | 9854.72 | 10885.86 | 10370.29 | 6123.83 | 5532.73 | 5828.28 | 19490.56 | 23698.18 | 21594.37 | 14723.39 | 16165.79 | 15444.59 | 1.058199 | -0.48355 | 1.405959 |
| EPHB4 | VYIDPFTYEDPNEAVR | Phospho(Y) | 18859.02 | 12814.86 | 15836.94 | 5283.68 | 6050.77 | 5667.225 | 18016.01 | 22827.89 | 20421.95 | 9032.06 | 14936.57 | 11984.32 | 0.366827 | -0.76897 | 1.080433 |
| EPHB4 | QPHYSAFGSVGEWLR | Phospho(Y) | 4821.3 | 7005.42 | 5913.36 | 2459.88 | 2953 | 2706.44 | 5494.45 | 7459.28 | 6476.865 | 3551.04 | 4289.82 | 3920.43 | 0.131318 | -0.72428 | 0.534615 |
| EPPK1 | AVTGYTDPYTGQQISLFQAMQK | Phospho(Y) | 1992.41 | 3751.55 | 2871.98 | 1937.14 | 1409.26 | 1673.2 | 1061.23 | 1662.28 | 1361.755 | 3285.16 | 2996 | 3140.58 | -1.07658 | 1.205564 | 0.908421 |
| ERBB2 | LLDIDETEYHADGGKVPIK | Phospho(Y) | 1043.93 | 980.35 | 1012.14 | 48.61 | 332.72 | 190.665 | 696.76 | 81.81 | 389.285 | 68.41 | 696.2 | 382.305 | -1.37851 | -0.0261 | 1.003684 |
| ERBB2 | GTPTAENPEYLGLDVPV | Phospho(Y) | 6875.36 | 8569.81 | 7722.585 | 4702.84 | 5823.11 | 5262.975 | 6282.92 | 6448.44 | 6365.68 | 5335.08 | 7663.99 | 6499.535 | -0.27877 | 0.030022 | 0.304458 |
| ERBB2IP | AQIPEGDYLSYR | Phospho(Y) | 15276.54 | 17973.9 | 16625.22 | 6406.64 | 5778.8 | 6092.72 | 30302.15 | 31299.87 | 30801.01 | 20822.52 | 21678.74 | 21250.63 | 0.889604 | -0.53547 | 1.802347 |
| ERBB2IP | IYDILSDNGPQQPSTTVK | Phospho(Y) | 890.93 | 1028.09 | 959.51 | 121.28 | 393.79 | 257.535 | 1715.68 | 1443.66 | 1579.67 | 97.71 | 1352.86 | 725.285 | 0.719253 | -1.123 | 1.493779 |
| ERBB3 | SLEATDSAFDNPDYWHSR | Phospho(Y) | 4119.18 | 5377.19 | 4748.185 | 810.9 | 847.8 | 829.35 | 6745.68 | 8268.21 | 7506.945 | 1384.99 | 1976.4 | 1680.695 | 0.66085 | -2.15917 | 1.019005 |

| | | | | | | | | | | | | | | | | | |
|---|---|---|---|---|---|---|---|---|---|---|---|---|---|---|---|---|---|
| ERBB3 | AFQGPGHQAPHVHYAR | Phospho(Y) | 17732.75 | 18972.02 | 18352.39 | 1728.86 | 1160.59 | 1444.725 | 6797.2 | 8971.37 | 7884.285 | 156.55 | 656.11 | 406.33 | -1.21892 | -4.27826 | -1.83007 |
| ESYT1 | HLSPYATLTVGDSSHK | Phospho(Y) | 1102.21 | 1547.84 | 1325.025 | 452.9 | 562.56 | 507.73 | 2226.94 | 2531.75 | 2379.345 | 90.91 | 1878.59 | 984.75 | 0.844545 | -1.27274 | 0.955696 |
| F11R | VIYSQPSAR | Phospho(Y) | 729.1 | 1152.61 | 940.855 | 2028.65 | 2338.09 | 2183.37 | 39.97 | 66.68 | 53.325 | 324.27 | 1413.57 | 868.92 | -4.14109 | 4.026339 | -1.32926 |
| FAM102A | VSSGVYEPVVIESH | Phospho(Y) | 1031.38 | 1187.89 | 1109.635 | 156.01 | 134.32 | 145.165 | 396.13 | 1669.26 | 1032.695 | 1315.04 | 983.63 | 1149.335 | -0.10367 | 0.154385 | 2.985034 |
| FAM111B | NYYFCSLPR | Phospho(Y) | 420.88 | 384.7 | 402.79 | 273.51 | 366.85 | 320.18 | 17.29 | 36.54 | 26.915 | 617.82 | 463.19 | 540.505 | -3.90355 | 4.327826 | 0.755425 |
| FAM120A, Y418 | NLTEQNSYSNIPHEGK | Phospho(Y) | 0 | 47.96 | 23.98 | 71.82 | 94.29 | 83.055 | 2 | 533.1 | 267.55 | 891.38 | 357.91 | 624.645 | 3.479905 | 1.223228 | 2.910898 |
| FAM59A | SELPYEELWLEEGKPSHQPLTR | Phospho(Y) | 5371.71 | 6388.74 | 5880.225 | 3124.99 | 3416.63 | 3270.81 | 9477.23 | 13163.49 | 11320.36 | 7223.2 | 8467.95 | 7845.575 | 0.944977 | -0.52897 | 1.262231 |
| FAM59A | QWTTITAHSLEEGHYVIGPK | Phospho(Y) | 1584.26 | 1800.11 | 1692.185 | 716.14 | 777.35 | 746.745 | 1133.51 | 1577.22 | 1355.365 | 1011.96 | 1374.12 | 1193.04 | -0.32021 | -0.18404 | 0.675955 |
| FAM83B | EGYVSHHNTPAQSFANR | Phospho(Y) | 6521.85 | 6345.46 | 6433.655 | 8089.56 | 10524.81 | 9307.185 | 5408.96 | 6169.12 | 5789.04 | 4540.16 | 3645.51 | 4092.835 | -0.15231 | -0.50022 | -1.18524 |
| FAM83B | GIYTLNEHDK | Phospho(Y) | 123.03 | 150.63 | 136.83 | 986.67 | 1040.2 | 1013.435 | 25.51 | 131.67 | 78.59 | 398.13 | 0 | 199.065 | -0.79997 | 1.340822 | -2.34794 |
| FAM83B | GIYTLNEHDKYNIR | Phospho(Y) | 0 | 174.25 | 87.125 | 243.34 | 0 | 121.67 | 0.04 | 0 | 0.02 | 0 | 34.68 | 17.34 | -12.0889 | 9.759888 | -2.8108 |
| FAM83F | AKAPAPAPAESGESLAYWPDR | Phospho(Y) | 33.71 | 140.56 | 87.135 | 786.97 | 839.39 | 813.18 | 103.65 | 440.39 | 272.02 | 114.5 | 199.42 | 156.96 | 1.642388 | -0.79332 | -2.37318 |
| FAM83F | APAPAPAESGESLAYWPDR | Phospho(Y) | 400.43 | 258.59 | 329.51 | 2556.05 | 2250.05 | 2403.05 | 926.28 | 1000.02 | 963.15 | 364.77 | 534.59 | 449.68 | 1.547438 | -1.09886 | -2.4179 |
| FER | VQENDGKEPPPVVNYEEDAR | Phospho(Y) | 453.85 | 1802.88 | 1128.365 | 2406.29 | 3292.47 | 2849.38 | 1374.5 | 1807.42 | 1590.96 | 2221.86 | 2389.25 | 2305.555 | 0.495664 | 0.535217 | -0.30553 |
| FGB | YRGTAGNALMDGASQLMGENR | Phospho(Y)Phosph | 1896.86 | 3000.9 | 2448.88 | 7899.72 | 7530.39 | 7715.055 | 5188.24 | 7732.02 | 6460.13 | 2024.92 | 1171.61 | 1598.265 | 1.399441 | -2.01506 | -2.27117 |
| FKBP15 | LASLFGLDQAAAGHGNEFFQYTAPK | Phospho(Y) | 1824.54 | 2524.6 | 2174.57 | 586.07 | 671.25 | 628.66 | 1721.45 | 2019.77 | 1870.61 | 1486.35 | 1880.41 | 1683.38 | -0.21722 | -0.15215 | 1.421009 |
| FLOT1 | VSAQYLSEIEMAK | Phospho(Y) | 2470.75 | 2171.28 | 2321.015 | 61.75 | 100.05 | 80.9 | 2712.63 | 3389.16 | 3050.895 | 1475.62 | 2265.9 | 1870.76 | 0.394477 | -0.70561 | 4.531341 |
| FLOT1 | AQADLAYQLQVAK | Phospho(Y) | 2676.68 | 2217.9 | 2447.29 | 106.87 | 184.58 | 145.725 | 3087.22 | 3561.83 | 3324.525 | 41.22 | 1581.44 | 811.33 | 0.441963 | -2.03479 | 2.47704 |
| FRK | VDNEDIYESRHEIK | Phospho(Y) | 11116.68 | 16678.62 | 13897.65 | 916.54 | 988.82 | 952.68 | 2523.7 | 32769.04 | 17646.37 | 32598.1 | 21095.49 | 26846.8 | 0.34453 | 0.605378 | 4.816614 |
| FRK | VDNEDIYESRHEIKLPVK | Phospho(Y) | 1768.22 | 1289.57 | 1528.895 | 113.58 | 68.64 | 91.11 | 861.86 | 744.2 | 803.03 | 1400.43 | 1410.84 | 1405.635 | -0.92896 | 0.807696 | 3.947469 |
| FRK | LEDYFETDSSYSDANNFIR | Phospho(Y) | 20864.48 | 24695.16 | 22779.82 | 2813.22 | 3618.13 | 3215.675 | 20990.29 | 26778.99 | 23884.64 | 38627.27 | 39765.57 | 39196.42 | 0.068327 | 0.714639 | 3.607528 |
| FRK | HGHYFVALFDYQAR | Phospho(Y) | 90741.37 | 119391.3 | 105066.3 | 11836.52 | 11752.27 | 11794.4 | 80670.49 | 97527.35 | 89098.92 | 92674.48 | 88302.55 | 90488.52 | -0.23782 | 0.022327 | 2.939633 |
| FRK | WKLEDYFETDSSYSDANNFIR | Phospho(Y) | 1100.72 | 3169.56 | 2135.14 | 28.75 | 38.75 | 33.75 | 187.86 | 602.49 | 395.175 | 47.97 | 429.12 | 238.545 | -2.43377 | -0.72823 | 2.821302 |
| FRS2 | TALLNYENLPSLPPVWEAR | Phospho(Y) | 1324.36 | 1453.77 | 1389.065 | 225.39 | 319.04 | 272.215 | 1454.89 | 77.95 | 766.42 | 670.34 | 1420.49 | 1045.415 | -0.85791 | 0.447869 | 1.941257 |
| FRS2 | LVYENINGLSIPSASGVR | Phospho(Y) | 7838.72 | 16166.46 | 12002.59 | 7682.86 | 7918.87 | 7800.865 | 7665.08 | 9253.8 | 8459.44 | 17230.17 | 9969.26 | 13599.72 | -0.50471 | 0.684942 | 0.80187 |
| FYN | LIEDNEYTAR | Phospho(Y) | 5035.94 | 6103.76 | 5569.85 | 2214.38 | 1778.61 | 1996.495 | 1275.06 | 2049.7 | 1662.38 | 8801.52 | 9330.31 | 9065.915 | -1.74439 | 2.447202 | 2.182983 |
| FYN | GAYSLSIR | Phospho(Y) | 321.97 | 826.05 | 574.01 | 349.63 | 360.08 | 354.855 | 86.33 | 517.3 | 301.815 | 461.85 | 1364.1 | 912.975 | -0.92741 | 1.596911 | 1.363346 |
| G6PD | NSYVAGQYDDAASYQR | Phospho(Y) | 0 | 503.43 | 251.715 | 234.25 | 394.92 | 314.585 | 113.69 | 0 | 56.845 | 1181.57 | 1799.95 | 1490.76 | -2.14669 | 4.712871 | 2.244526 |
| G6PD | GGYFDEFGIIR | Phospho(Y) | 248.43 | 246.24 | 247.335 | 0 | 394.17 | 197.085 | 289.01 | 329.22 | 309.115 | 0 | 314.82 | 157.41 | 0.321677 | -0.97362 | -0.32429 |
| GGA2 | RPGQAPPDQEALQVVYER | Phospho(Y) | 1024.14 | 1208.49 | 1116.315 | 140.87 | 145 | 142.935 | 736.96 | 1436.86 | 1086.91 | 317.88 | 1232.4 | 775.14 | -0.03851 | -0.4877 | 2.439098 |
| GIT1 | LQPFHSTELEDDAIYSVHVPAGLYR | Phospho(Y) | 10149.76 | 13418.51 | 11784.14 | 2267.31 | 3488.96 | 2878.135 | 7526.09 | 11600.87 | 9563.48 | 4331.54 | 6774.78 | 5553.16 | -0.30124 | -0.78423 | 0.948175 |
| GIT1 | HGSGADSDYENTQSGDPLLGLEGK | Phospho(Y) | 0 | 424.02 | 212.01 | 310.14 | 301.3 | 305.72 | 8.19 | 73.3 | 40.745 | 0 | 259.84 | 129.92 | -2.37944 | 1.672929 | -1.23459 |
| GNAI3 | NNLKECGLY | Phospho(Y) | 28.65 | 33.95 | 31.3 | 96.48 | 110.26 | 103.37 | 0 | 32.39 | 16.195 | 0 | 36.48 | 18.24 | -0.95061 | 0.171557 | -2.50264 |
| GOLGA4 | EAEEKILTLENQVYSMK | Phospho(Y)Phosph | 22.95 | 0 | 11.475 | 260.11 | 0 | 130.055 | 11.73 | 0 | 5.865 | 66.15 | 11.04 | 38.595 | -0.96829 | 2.718211 | -1.75264 |
| GOLGA4 | NVYATTVGTPYK | Phospho(Y) | 316.67 | 839.12 | 577.895 | 394.36 | 427.25 | 410.805 | 527.16 | 739.9 | 633.53 | 1255.61 | 1743.35 | 1499.48 | 0.132606 | 1.242977 | 1.867937 |
| GOLGA5 | NTDYTELHQQNTDLIYQTGPK | Phospho(Y) | 5261.07 | 7506.57 | 6383.82 | 2384.03 | 1702.33 | 2043.18 | 11014.01 | 15451.19 | 13232.6 | 8734.75 | 14991.01 | 11862.88 | 1.051605 | -0.15764 | 2.537566 |
| GPRC5A | AHAWPSPYKDYEVK | Phospho(Y)Phosph | 9524.18 | 16482.23 | 13003.21 | 1590.29 | 2391.01 | 1990.65 | 8189.98 | 12638.03 | 10414.01 | 11967.45 | 15026.87 | 13497.16 | -0.32034 | 0.374131 | 2.761344 |
| GPRC5A | AHAWPSPYKDYEVKK | Phospho(Y)Phosph | 1351.58 | 3020.19 | 2185.885 | 93.92 | 158.61 | 126.265 | 87.97 | 587.95 | 337.96 | 802.4 | 878.2 | 840.3 | -2.69329 | 1.314052 | 2.73445 |
| GPRC5A | AHAWPSPYKDYEVK | Phospho(Y) | 1281.1 | 1313.74 | 1297.42 | 211 | 191.15 | 201.075 | 819.63 | 1039.9 | 929.765 | 1019.15 | 1260.72 | 1139.935 | -0.48071 | 0.294014 | 2.503146 |
| GPRC5A | AYSQEEITQGFEETGDTLYAPYSTHFQLQNQPPQK | Phospho(Y) | 30692.58 | 38492.59 | 34592.59 | 7217.24 | 9019.99 | 8118.615 | 29422.9 | 43474.36 | 36448.63 | 31967.51 | 41229.24 | 36598.38 | 0.075402 | 0.005915 | 2.172474 |
| GPRC5A | AHAWPSPYK | Phospho(Y) | 18920.38 | 15764.27 | 17342.33 | 5847.91 | 5783.17 | 5815.54 | 11623.96 | 16904.28 | 14264.12 | 18987.77 | 16365.69 | 17676.73 | -0.28191 | 0.309461 | 1.603866 |
| GPRC5C | VPSEGAYDIILPR | Phospho(Y) | 14328.88 | 17475.51 | 15902.2 | 7567.29 | 7359.38 | 7463.335 | 16723.02 | 20216.35 | 18469.69 | 25483.51 | 29649.66 | 27566.59 | 0.215933 | 0.577761 | 1.885028 |
| GPRC5C | AEDMYSAQSHQAATPPK | Phospho(Y) | 24.73 | 148.82 | 86.775 | 184.13 | 495.03 | 339.58 | 0 | 1 | 0.5 | 0 | 93.27 | 46.635 | -7.43921 | 6.543341 | -2.86427 |
| GSK3A | GEPNVSYICSR | Phospho(Y) | 2251134 | 2546563 | 2398848 | 2158912 | 2073961 | 2116436 | 2033829 | 2496554 | 2265192 | 4250147 | 3809944 | 4030045 | -0.08271 | 0.831163 | 0.929159 |
| HCK | NRPEERPTFEYIQSVLDDFYTATESQYQQQP | Phospho(Y) | 9817.95 | 25993.57 | 17905.76 | 4597.22 | 4808.02 | 4702.62 | 6673.1 | 11204.51 | 8938.805 | 5433.68 | 11888.81 | 8661.245 | -1.00227 | -0.04551 | 0.88111 |
| HELZ | HINLPLPAPHAQYAIPNR | Phospho(Y) | 35.35 | 243.7 | 139.525 | 467.34 | 258.4 | 362.87 | 0.38 | 0 | 0.19 | 132.67 | 353.67 | 243.17 | -9.52031 | 10.32175 | -0.57749 |
| HGS | VCEPCYEQLNR | Phospho(Y) | 13.38 | 1522.29 | 767.835 | 132.35 | 189.99 | 161.17 | 8.61 | 13.05 | 10.83 | 1815.62 | 97.96 | 956.79 | -6.14769 | 6.465097 | 2.569619 |
| HIPK2 | AVCSTYLQSR | Phospho(Y) | 76414.51 | 84763.01 | 80588.76 | 21878.44 | 32703.43 | 27290.94 | 13012.54 | 13931.22 | 13471.88 | 52946.9 | 46867.81 | 49907.36 | -2.58063 | 1.889301 | 0.870831 |
| HIPK3 | TVCSTYLQSR | Phospho(Y) | 317.61 | 356.81 | 337.21 | 690.15 | 499.82 | 594.985 | 34.17 | 38.49 | 36.33 | 101.95 | 0 | 50.975 | -3.21441 | 0.488629 | -3.54499 |
| HIST1H4A | ISGLIYEETR | Phospho(Y) | 4625.34 | 5060.2 | 4842.77 | 600.26 | 600.97 | 600.615 | 2340.38 | 6083.65 | 4212.015 | 6812.68 | 6077.04 | 6444.86 | -0.20132 | 0.613638 | 3.423637 |
| HMGB1 | RPPSAFFLFCSEYRPK | Phospho(Y) | 1368.44 | 1876.64 | 1622.54 | 297.64 | 651.33 | 474.485 | 451.25 | 846.99 | 649.12 | 538.24 | 669.33 | 603.785 | -1.3217 | -0.10445 | 0.347672 |
| HNRNPA1 | SSGPYGGGGQYFAKPR | Phospho(Y) | 25.74 | 1894.52 | 960.13 | 1218.36 | 1409.3 | 1313.83 | 6.97 | 53.59 | 30.28 | 3335.99 | 2877.42 | 3106.705 | -4.98679 | 6.680876 | 1.241607 |
| HNRNPA2B1 | NMGGPYGGGNYGPGGSGGSGGYGGR | Phospho(Y) | 7064 | 6795.69 | 6929.845 | 5378.93 | 8001.19 | 6690.06 | 10847.26 | 8957.81 | 9902.535 | 11653.66 | 10161.87 | 10907.77 | 0.514975 | 0.139486 | 0.705264 |
| HNRNPH1 | DLNYCFSGMSDHR | Phospho(Y) | 5923.1 | 6306.11 | 6114.605 | 3105.24 | 3709.21 | 3407.225 | 2223.67 | 3214.22 | 2718.945 | 8301.09 | 5714.05 | 7007.57 | -1.16921 | 1.365867 | 1.040317 |
| HNRNPK | IITITGTQDQIQNAQYLLQNSVK | Phospho(Y) | 504.62 | 852.28 | 678.45 | 671.98 | 496.72 | 584.35 | 1027.68 | 1237.4 | 1132.54 | 610.34 | 800.96 | 705.65 | 0.739248 | -0.68254 | 0.27212 |
| HRAS | QGVEDAFYTLVR | Phospho(Y) | 410.46 | 465.26 | 437.86 | 95.44 | 35.43 | 65.435 | 495.92 | 553.63 | 524.775 | 336.16 | 413.86 | 375.01 | 0.261229 | -0.48477 | 2.518795 |
| HSP90AA1 | IRYESLTDPSK | Phospho(Y) | 9193.84 | 7658 | 8425.92 | 4142.73 | 3693.93 | 3918.33 | 9664.61 | 13900.37 | 11782.49 | 17718.81 | 18148.63 | 17933.72 | 0.483738 | 0.60603 | 2.194364 |
| HSP90AB1 | LVSSPCCIVTSTYGWTANMER | Phospho(Y) | 3562.09 | 3701.41 | 3631.75 | 3621.03 | 3284.52 | 3452.775 | 2404.72 | 3849.87 | 3127.295 | 3537.97 | 4125.96 | 3831.965 | -0.21575 | 0.293169 | 0.150328 |
| HSPA1B | ELEQVCNPIISGLYQGAGGPGPGGFGAQGPK | Phospho(Y) | 32879.23 | 45221.63 | 39050.43 | 11146.32 | 11515.94 | 11331.13 | 24473.4 | 28309.25 | 26391.33 | 34886.89 | 36537.03 | 35711.96 | -0.56527 | 0.436344 | 1.656116 |
| HSPA1B | KELEQVCNPIISGLYQGAGGPGPGGFGAQGPK | Phospho(Y) | 2490.09 | 2973.9 | 2731.995 | 3276.22 | 1193.43 | 2234.825 | 2115.03 | 2146.62 | 2130.825 | 2293.6 | 2462.58 | 2378.09 | -0.35854 | 0.158391 | 0.089641 |
| HSPA4 | KEDIYAVEIVGGATR | Phospho(Y) | 2467.15 | 2765.74 | 2616.445 | 821.59 | 1136.64 | 979.115 | 3011.33 | 2691.18 | 2851.255 | 3236.24 | 4349.78 | 3793.01 | 0.123989 | 0.411746 | 1.953793 |
| HSPA4 | EDIYAVEIVGGATR | Phospho(Y) | 17.01 | 263.86 | 140.435 | 21.15 | 217.23 | 119.19 | 80.97 | 202.1 | 141.535 | 0 | 324.29 | 162.145 | 0.011256 | 0.196126 | 0.444021 |
| HSPA8 | GPAVGIDLGTTYSCVGVFQHGK | Phospho(Y) | 830.61 | 1602.37 | 1216.49 | 879.31 | 1013.81 | 946.56 | 272.63 | 267.43 | 270.03 | 953.14 | 928.49 | 940.815 | -2.17153 | 1.800791 | -0.00878 |
| HSPH1 | NAVEEYVYEFRDK | Phospho(Y) | 1474.19 | 1106.5 | 1290.345 | 27.92 | 90.15 | 59.035 | 481.01 | 494.49 | 487.75 | 1600.01 | 1385.94 | 1492.975 | -1.40354 | 1.613976 | 4.660476 |
| HSPH1 | NAVEEYVYEFR | Phospho(Y) | 1035.26 | 1537.43 | 1286.345 | 188.91 | 190.24 | 189.575 | 1127.75 | 1524.31 | 1326.03 | 2008.68 | 1841.06 | 1924.87 | 0.043836 | 0.537648 | 3.34392 |
| IFITM3 | MNHTVQTFFSPVNSGQPPNYEMLKEEHEVAVLGAPHNF | Phospho(Y) | 28069.2 | 35114.68 | 31591.94 | 48409.91 | 43770.09 | 46090 | 23949.86 | 30487.23 | 27218.55 | 8249.35 | 8840 | 8544.675 | -0.21497 | -1.67149 | -2.43136 |
| IFNGR1 | APTSFGYDKPHVLVDLLVDDSGK | Phospho(Y) | 2175.36 | 3729.45 | 2952.405 | 2836.14 | 3913.18 | 3374.66 | 2546.22 | 3514.11 | 3030.165 | 1776.71 | 1999.62 | 1888.165 | 0.037506 | -0.68241 | -0.83776 |
| IFT27 | GSDGAHFQKSYTLTTGMDLVVK | Phospho(ST)Phosp | 205032.9 | 219474.5 | 212253.7 | 46865.25 | 54409.71 | 50637.48 | 79560.36 | 97052.35 | 88306.36 | 59667.75 | 99024.75 | 79346.25 | -1.2652 | -0.15436 | 0.647956 |
| IFT27 | QSDGAHFQKSYTLTTGMDLVVK | Phospho(ST)Phosp | 141509.7 | 222282.5 | 181896.1 | 112348.7 | 114075.2 | 113212 | 86061.7 | 111474 | 98767.83 | 108613.9 | 99935.11 | 104274.5 | -0.881 | 0.078273 | -0.11864 |
| IFT27 | SSDGAHFQKSYTLTTGMDLVVK | Phospho(ST)Phosp | 80797.75 | 85450.17 | 83123.96 | 83645.34 | 98224.71 | 90935.03 | 215428.4 | 292574 | 254001.2 | 76127.85 | 91033.59 | 83580.72 | 1.611499 | -1.60359 | -0.12167 |
| IFT27 | MSDGAHFQKSYTLTTGMDLVVK | Phospho(ST)Phosp | 450.62 | 272.2 | 361.41 | 770.37 | 1305.22 | 1037.795 | 2407.17 | 4327.58 | 3367.375 | 576.98 | 647.11 | 612.045 | 3.219916 | -2.45991 | -0.76181 |
| IGF1R | QPYAHMNGGR | Phospho(Y) | 17.31 | 4.45 | 10.88 | 994.13 | 859.4 | 926.765 | 25.24 | 0.74 | 12.99 | 57.39 | 52.11 | 54.75 | 0.255723 | 2.075458 | -4.08127 |
| IGF1R, Y1014 | ELGQGSFGMVYEGVAK | Phospho(Y) | 363.07 | 297.69 | 330.38 | 36.64 | 0 | 18.32 | 1000.7 | 958.92 | 979.81 | 252.08 | 78.69 | 165.385 | 1.568376 | -2.56667 | 3.174337 |
| IGF1R, Y1161,1165,1166 | DIYETDYYRK | Phospho(Y) | 51404.34 | 48805.14 | 50104.74 | 52471.73 | 64727.01 | 58599.37 | 64082.62 | 76050.6 | 70066.61 | 123097.1 | 149846.3 | 136471.7 | 0.48378 | 0.961803 | 1.219645 |
| IGF1R, Y1161/Y1165 | DIYETDYYRK | Phospho(Y)Phosph | 47857.89 | 65327.64 | 56592.77 | 92666.42 | 146652.7 | 119659.6 | 57176.99 | 40099.6 | 48638.3 | 236795 | 258714.7 | 247754.8 | -0.21852 | 2.348749 | 1.049977 |
| INPPL1 | NSFNNPAYYVLEGVPHQLLPPEPPSPAR | Phospho(Y) | 144227.1 | 206467.6 | 175347.4 | 85695.88 | 86242.69 | 85969.29 | 207593.8 | 288869 | 248231.4 | 178319.5 | 206799.9 | 192559.7 | 0.50147 | -0.36638 | 1.163413 |
| INPPL1 | ERLYEWISIDKDEAGAK | Phospho(Y) | 4908.34 | 6162.83 | 5535.585 | 466.29 | 2086.86 | 1276.575 | 3444.04 | 6927.24 | 5185.64 | 751.94 | 4134.38 | 2443.16 | -0.09421 | -1.08577 | 0.93647 |
| INPPL1 | LYEWISIDKDEAGAK | Phospho(Y) | 2793.77 | 1767.63 | 2280.7 | 243.41 | 1098.78 | 671.095 | 3856.78 | 3218.85 | 3537.815 | 29.06 | 1944.43 | 986.745 | 0.633382 | -1.84211 | 0.55616 |
| IQGAP1 | LGLAPQIQDLYGK | Phospho(Y) | 1914.34 | 2163.14 | 2038.74 | 932.52 | 1215.16 | 1073.84 | 2176.29 | 1826.76 | 2001.525 | 749.62 | 1929.32 | 1339.47 | -0.02658 | -0.57944 | 0.318883 |
| IRS1 | LCGAAGGLENGLNYIDLDLVK | Phospho(Y) | 854.58 | 2120.49 | 1487.535 | 467.5 | 731.12 | 599.31 | 1099.6 | 1817.52 | 1458.56 | 499.26 | 1068.48 | 783.87 | -0.02838 | -0.89586 | 0.387312 |
| IRS1 | GGHHRPDSSTLHTDDGYMPMSPGVAPVPSGR | Phospho(Y) | 77688.11 | 112656.7 | 95172.38 | 68775.34 | 63569.51 | 66172.43 | 186115.7 | 214459.8 | 200287.8 | 90225.21 | 72303.32 | 81264.27 | 1.07346 | -1.30138 | 0.296391 |
| IRS1 | GEEELSNYICMGGK | Phospho(Y) | 4949.31 | 6457.98 | 5703.645 | 6281.05 | 5559 | 5920.025 | 8744.25 | 12134.28 | 10439.27 | 7558.89 | 5936.94 | 6747.915 | 0.872064 | -0.62951 | 0.188839 |
| IRS2 | APYTCGGDSDQYVLMSSPVGR | Phospho(Y) | 15012.79 | 16348.42 | 15680.61 | 584.73 | 877.28 | 731.005 | 12586.47 | 14525.16 | 13555.82 | 6221.52 | 9725.75 | 7973.635 | -0.21007 | -0.7656 | 3.447284 |
| IRS2 | DGGGGGEFYGYMTMDRPLSHCGR | Phospho(Y) | 35108.23 | 44863.25 | 39985.74 | 816.62 | 467.58 | 642.1 | 19427.34 | 27023.86 | 23225.6 | 7094.26 | 6594.05 | 6844.155 | -0.78377 | -1.76277 | 3.414003 |
| IRS2 | AAAGDAPPAAAPAASCSASLPGALGGSAGAAGAEDSYG | Phospho(Y) | 3306.76 | 3601.34 | 3454.05 | 634.76 | 458.51 | 546.635 | 3854.46 | 5747.4 | 4800.93 | 2064.09 | 3415.29 | 2739.69 | 0.475025 | -0.8093 | 2.325363 |
| IRS2 | QRPVPQPSSASLDEYTLMR | Phospho(Y) | 2131.92 | 2843.41 | 2487.665 | 31.81 | 33.52 | 32.665 | 2942.53 | 2633.52 | 2788.025 | 147.32 | 84.75 | 116.035 | 0.164451 | -4.58661 | 1.828742 |
| IRS2, Y632 | VAYHPYPEDYGDIEIGSHR | Phospho(Y) | 741.82 | 848.05 | 794.935 | 8.39 | 10.86 | 9.625 | 1365.13 | 2166.99 | 1766.06 | 43.14 | 247.96 | 145.55 | 1.151626 | -3.60095 | 3.918584 |
| IRS2, Y675 | SDDYMPMSPASVSAPK | Phospho(Y) | 2352.32 | 2250.13 | 2301.225 | 3.84 | 73.65 | 38.745 | 8117.81 | 12665.68 | 10391.75 | 1410.04 | 1352.17 | 1381.105 | 2.174964 | -2.91154 | 5.155669 |
| IRS2, Y75 | GPGAGGDEATAGGGSAPQPPRLEYYESEK | Phospho(Y) | 1102.29 | 642.61 | 872.45 | 44.03 | 65.7 | 54.865 | 1721.16 | 1966.92 | 1844.04 | 607 | 560.87 | 583.935 | 1.079726 | -1.65899 | 3.41185 |
| IRS2, Y766 | LLPNGDYLNVSPSDAVTTGTPPDFFSAALHPGGEPLR | Phospho(Y) | 10387.04 | 16434.16 | 13410.6 | 342.96 | 340.11 | 341.535 | 21596.92 | 27471.38 | 24534.15 | 1811.58 | 2568.13 | 2189.855 | 0.871418 | -3.48588 | 2.68073 |
| ITGB1 | WDTGENPIYK | Phospho(Y) | 2040.52 | 2448.37 | 2244.445 | 87.33 | 61.24 | 74.285 | 1414.54 | 1679.02 | 1546.78 | 458.5 | 3028.68 | 1743.59 | -0.53709 | 0.172793 | 4.552846 |
| ITSN2 | LIYLVPEK | Phospho(Y) | 2523.98 | 2415.76 | 2469.87 | 792.66 | 774.28 | 783.47 | 2092.68 | 3675.94 | 2884.31 | 3852.26 | 3880.79 | 3866.525 | 0.223791 | 0.422811 | 2.303088 |
| ITSN2 | REEPEALYAAVNK | Phospho(Y) | 114398.1 | 98357.06 | 106377.6 | 56527.66 | 63701.08 | 60114.37 | 93134.64 | 116413.5 | 104774.1 | 220309.2 | 162041.1 | 191175.1 | -0.02191 | 0.867613 | 1.669113 |
| ITSN2 | EEPEALYAAVNK | Phospho(Y) | 17736.59 | 20352.27 | 19044.43 | 12384.25 | 11591.86 | 11988.06 | 13998.22 | 21967.36 | 17982.79 | 28660.69 | 25780.41 | 27220.55 | -0.08275 | 0.598079 | 1.183099 |
| JAK2 | EVGDYGQLHETEVLLK | Phospho(Y) | 6286.65 | 5337.04 | 5811.845 | 653.65 | 3175.03 | 1914.34 | 5520.97 | 5598.67 | 5559.82 | 2025.76 | 4880.41 | 3453.085 | -0.06396 | -0.68715 | 0.851039 |
| KATNAL2 | GLLLYGPPGTGK | Phospho(Y) | 470.4 | 396.64 | 433.52 | 235.5 | 150.65 | 193.075 | 439.39 | 526.63 | 483.01 | 330.03 | 602.14 | 466.085 | 0.155955 | -0.05146 | 1.271432 |
| KAZN | QSQQTLYHSHPPHPADR | Phospho(Y) | 10046.9 | 10533.9 | 10290.4 | 2785.73 | 1587.38 | 2186.555 | 12643.77 | 13140.69 | 12892.23 | 18843.83 | 15810.72 | 17327.28 | 0.325203 | 0.426543 | 2.986313 |
| KAZN | TDLVSQMQQLYATLESREEQLR | Phospho(Y) | 370.1 | 510.79 | 440.445 | 42.22 | 2032.27 | 1037.245 | 122.75 | 77.6 | 100.175 | 105.17 | 98.07 | 101.62 | -2.13644 | 0.020662 | -3.3515 |
| KIAA1217 | NEGFYADPYLYHEGR | Phospho(Y) | 9894.75 | 10148.95 | 10021.85 | 661.46 | 678.39 | 669.925 | 7639.11 | 7854.01 | 7746.56 | 10093.66 | 10617.07 | 10355.37 | -0.37152 | 0.418751 | 3.950235 |
| KIAA1217 | NVYYELNDVR | Phospho(Y) | 5727.43 | 6669.39 | 6198.41 | 952.84 | 1145.6 | 1049.22 | 3894.22 | 4135.86 | 4015.04 | 7145.03 | 5447.36 | 6296.195 | -0.62648 | 0.649066 | 2.585163 |
| KIAA1671 | ATFFAVTYQIPNTQK | Phospho(Y) | 1001 | 883.32 | 942.16 | 564.69 | 645.89 | 605.29 | 885.49 | 599.28 | 742.385 | 1194.74 | 1037.28 | 1116.01 | -0.3438 | 0.58811 | 0.882652 |
| KRT18 | STFSTNYR | Phospho(Y) | 1909.89 | 2545.17 | 2227.53 | 4562.09 | 5786.97 | 5174.53 | 146.82 | 299.24 | 223.03 | 3652.8 | 3063.91 | 3358.355 | -3.32014 | 3.912445 | -0.62367 |
| LAMTOR1 | IAAYAYSALSQIR | Phospho(Y) | 1628.35 | 1952.65 | 1790.5 | 488.02 | 433.22 | 460.62 | 1049.86 | 1363.82 | 1206.84 | 979.37 | 1158.52 | 1068.945 | -0.56913 | -0.17505 | 1.214539 |
| LAPTM4A | MPEKEPPPPYLPA | Phospho(Y) | 34077.23 | 38888.68 | 36482.96 | 3837.78 | 4087.8 | 3962.79 | 41940.66 | 41152.29 | 41546.48 | 12005.02 | 11575.81 | 11790.42 | 0.187503 | -1.81711 | 1.573026 |
| LAPTM4A | NVPEIAVYPAFEAPPQYVLPTYEMAVK | Phospho(Y) | 10464.54 | 15357.07 | 12910.81 | 736.68 | 1319.87 | 1028.275 | 6900.87 | 8650.53 | 7775.7 | 1838.56 | 2031.68 | 1935.12 | -0.73153 | -2.00655 | 0.912197 |
| LASP1 | RPLEQQQPHHIPTSAPVYQQPQQQPVAQSYGGYK | Phospho(Y) | 52.12 | 77.08 | 64.6 | 174.56 | 323.85 | 249.205 | 2431.81 | 3199.83 | 2815.82 | 3814.49 | 4054.99 | 3934.74 | 5.445877 | 0.482713 | 3.980863 |
| LASP1 | RPLEQQQPHHIPTSAPVYQQPQQQPVAQSYGGYKEPAA | Phospho(Y) | 42063.76 | 51160.91 | 46612.34 | 8683.32 | 8145.3 | 8414.31 | 51727.97 | 77315.82 | 64521.9 | 96415.09 | 89012.67 | 92713.88 | 0.469077 | 0.522997 | 3.461868 |
| LDHA | DQLIYNLLKEEQTPQNK | Phospho(Y) | 6357.63 | 8694.3 | 7525.965 | 5245.41 | 5415.41 | 5330.41 | 4511.65 | 5945.66 | 5228.655 | 5933.24 | 6144.58 | 6038.91 | -0.52544 | 0.207848 | 0.180042 |
| LDHA | DQLIYNLLK | Phospho(Y) | 1999.82 | 2697.38 | 2348.6 | 2651.62 | 2811.05 | 2731.335 | 2295.84 | 3235.04 | 2765.44 | 2191.59 | 3406.89 | 2799.24 | 0.235708 | 0.017526 | 0.035429 |
| LOC647859 | NVSAGTQDVPSPPSDYVER | Phospho(Y) | 1397.15 | 1856.65 | 1626.9 | 1429.86 | 1528.11 | 1478.985 | 3229.04 | 4712.15 | 3970.595 | 2577.41 | 4101.39 | 3339.4 | 1.28723 | -0.24977 | 1.174981 |
| LPHN1 | NPLQGYYQVR | Phospho(Y) | 1881.74 | 1854.99 | 1868.365 | 896.22 | 1017.43 | 956.825 | 1209.66 | 1123.56 | 1166.61 | 76.09 | 1969.08 | 1022.585 | -0.67945 | -0.1901 | 0.095894 |
| LSR | AATSGVPSIYAPSTYAHLSPAK | Phospho(Y)Phosph | 1857.13 | 1783.3 | 1820.215 | 667.26 | 1047.34 | 857.3 | 2337.9 | 3501.74 | 2919.82 | 3345.99 | 5050.61 | 4198.3 | 0.681771 | 0.523926 | 2.291933 |
| LSR | SSSAGGQGSYVPLLR | Phospho(Y) | 420.45 | 754.05 | 587.25 | 685.59 | 592.13 | 638.86 | 856.01 | 988.26 | 922.135 | 1505.37 | 1865.3 | 1685.335 | 0.651003 | 0.869986 | 1.399464 |
| LSR | SGDLPYDGRLLEEAVR | Phospho(Y) | 879.88 | 1175.98 | 1027.93 | 182.06 | 278.79 | 230.425 | 658.27 | 1007.3 | 832.785 | 197.06 | 856.11 | 526.585 | -0.30373 | -0.66128 | 1.192369 |
| LSR | AATSGVPSIYAPSTYAHLSPAK | Phospho(Y) | 2262.22 | 2861.69 | 2561.955 | 4055.41 | 3860.61 | 3958.01 | 6338.31 | 8371.82 | 7355.065 | 7451.03 | 8293.53 | 7872.28 | 1.521493 | 0.098043 | 0.992006 |
| LYN | EKAEERPTFDYLQSVLDDFYTATEGQYQQQP | Phospho(Y) | 115.68 | 5234.09 | 2674.885 | 187.74 | 480.42 | 334.08 | 187.92 | 18.23 | 103.075 | 302.9 | 481.97 | 392.435 | -4.69771 | 1.928759 | 0.23226 |
| LYN | AEERPTFDYLQSVLDDFYTATEGQYQQQP | Phospho(Y) | 133323.1 | 226641.1 | 179982.1 | 221355.4 | 275834.9 | 248595.2 | 180672.7 | 1671.25 | 91171.97 | 288293.1 | 1233.11 | 144763.1 | -0.98119 | 0.667032 | -0.7801 |
| MAPK1 | VADPDHDHTGFLTEYVATR | Phospho(Y) | 44738.6 | 60365.44 | 52552.02 | 11050.74 | 11806.74 | 11428.74 | 40237.35 | 43075.03 | 41656.19 | 37165.1 | 49907.69 | 43536.4 | -0.33522 | 0.063691 | 1.929556 |
| MAPK13 | HADAEMTGYVVTR | Phospho(Y) | 1636.11 | 2121.08 | 1878.595 | 880.94 | 959.88 | 920.41 | 913.83 | 745.19 | 829.51 | 7072.48 | 5146.13 | 6109.305 | -1.17932 | 2.880677 | 2.73066 |
| MAPK14 | HTDDEMTGYVATR | Phospho(Y) | 22234.88 | 23989.03 | 23111.96 | 26173.12 | 32413.98 | 29293.55 | 3821.13 | 3469.05 | 3645.09 | 55944.1 | 50723.46 | 53333.78 | -2.66461 | 3.871023 | 0.864467 |
| MAPK3 | IADPEHDHTGFLTEYVATR | Phospho(Y) | 97275.55 | 101112 | 99193.78 | 23038.65 | 24592.66 | 23815.66 | 85475.98 | 170931.4 | 128203.7 | 89029.91 | 104671.8 | 96850.85 | 0.370116 | -0.4046 | 2.023854 |
| MAPK7 | GLCTSPAEHQYFMTEYVATR | Phospho(Y) | 298.46 | 412.95 | 355.705 | 0.95 | 114.14 | 57.545 | 407.23 | 0 | 203.615 | 0 | 83.69 | 41.845 | -0.80484 | -2.28272 | -0.45964 |
| MARK2 | DQQNLPYGVTPASPSGHSQGR | Phospho(Y) | 1115.37 | 2180.85 | 1648.11 | 6058.72 | 5965.22 | 6011.97 | 4709.2 | 4782.43 | 4745.815 | 5240.59 | 6936.23 | 6088.41 | 1.525843 | 0.35941 | 0.018228 |
| MDH2 | GYLGPEQLPDCLK | Phospho(Y) | 458.19 | 73.96 | 266.075 | 0.62 | 17.19 | 8.905 | 249.98 | 0.36 | 125.17 | 17.2 | 181.86 | 99.53 | -1.08794 | -0.33069 | 3.482444 |
| MGAM | QYDIHNLYGYSMAVATAEAAKTVFPNKR | Phospho(Y)Phosph | 119.77 | 26.7 | 73.235 | 228.14 | 918.98 | 573.56 | 5387.04 | 8014.38 | 6700.71 | 185.38 | 226.22 | 205.8 | 6.515637 | -5.025 | -1.4787 |
| MLLT4 | DLQYITVSK | Phospho(Y) | 359.85 | 306.37 | 333.11 | 226.44 | 204.43 | 215.435 | 77.19 | 95.82 | 86.505 | 610.43 | 475.37 | 542.9 | -1.94514 | 2.649831 | 1.333434 |
| MPP5 | VYESIGQYGGETVK | Phospho(Y) | 1583.48 | 2069.94 | 1826.71 | 611.54 | 665.95 | 638.745 | 3049.99 | 3599.66 | 3324.825 | 6486.43 | 3935.06 | 5210.745 | 0.864031 | 0.648211 | 3.028178 |
| MPZL1 | SLPSGSHQGPVIYAQLDHSGGHHSDK | Phospho(Y) | 1691.16 | 0 | 845.58 | 191.92 | 0 | 95.96 | 50.1 | 0 | 25.05 | 0 | 40.77 | 20.385 | -5.07706 | -0.2973 | -2.23493 |
| MREG | ALPEKEPLVSDNNPYSSFGATLVRDDEK | Phospho(Y) | 1586.31 | 1355.53 | 1470.92 | 110.45 | 107.02 | 108.735 | 1127.68 | 1686.43 | 1407.055 | 0 | 558.97 | 279.485 | -0.06404 | -2.33184 | 1.361954 |
| MREG | ALPEKEPLVSDNNPYSSFGATLVR | Phospho(Y) | 3200.97 | 3857.62 | 3529.295 | 974.16 | 1280.51 | 1127.335 | 3198.26 | 5400.97 | 4299.615 | 2647.07 | 3135.6 | 2891.335 | 0.284827 | -0.57247 | 1.358819 |
| MUC1 | YVPPSSTDRSPYEK | Phospho(Y) | 6368.53 | 8428.52 | 7398.525 | 363.7 | 406.79 | 385.245 | 6793.75 | 9832.16 | 8312.955 | 20221.33 | 17475.79 | 18848.56 | 0.168124 | 1.181021 | 5.612534 |
| MYO1D | HIDYFNNQIIVDLVEQQHK | Phospho(Y) | 39.12 | 819.26 | 429.19 | 177.16 | 111.69 | 144.425 | 707.86 | 949.4 | 828.63 | 552 | 693.36 | 622.68 | 0.949112 | -0.41224 | 2.10817 |
| MYO6 | SVTDYDFAPFLNNSPQQNPAAQIPAR | Phospho(Y) | 22.39 | 455.59 | 238.99 | 5642.19 | 229.88 | 2936.035 | 260.15 | 719.63 | 489.89 | 2595.52 | 1926.48 | 2261 | 1.035508 | 2.206431 | -0.37691 |
| MYO6 | SVTDYAQQNPAAQIPAR | Phospho(Y) | 454.39 | 334.54 | 394.465 | 6811.43 | 7336.25 | 7073.84 | 1398.36 | 2119.65 | 1759.005 | 2200.57 | 8080.32 | 5140.445 | 2.15679 | 1.547134 | -0.4606 |
| N4BP3 | NEPADYATLYYR | Phospho(Y) | 1007.79 | 1387.38 | 1197.585 | 404.91 | 252.55 | 328.73 | 3118.98 | 3500.64 | 3309.81 | 3852.58 | 2991.13 | 3421.855 | 1.46662 | 0.04803 | 3.379804 |
| NACC1 | AEDDAYTTFISETGK | Phospho(Y) | 476.16 | 666.61 | 571.385 | 0 | 208.58 | 104.29 | 658.35 | 1203.54 | 930.945 | 930.45 | 1052.65 | 991.55 | 0.704233 | 0.09099 | 3.249085 |
| NCK2 | IYDLNIPAFVK | Phospho(Y) | 292.78 | 410.66 | 351.72 | 208.16 | 227.61 | 217.885 | 290.83 | 462.65 | 376.74 | 150.67 | 260.83 | 205.75 | 0.099142 | -0.87268 | -0.08267 |
| NCKIPSD | QHSLPSSEHLGADGGLYQIPPQPR | Phospho(Y) | 15696.73 | 18406.84 | 17051.79 | 7311.75 | 6470.84 | 6891.295 | 13458.85 | 19023.12 | 16240.99 | 10983.08 | 11889.41 | 11436.25 | -0.07028 | -0.50603 | 0.730766 |
| NEDD9 | EKDYDFPPPMR | Phospho(Y) | 12603.21 | 15073.86 | 13838.54 | 2758.41 | 4020.44 | 3389.425 | 12920.81 | 15258.41 | 14089.61 | 7058.79 | 6074.18 | 6566.485 | 0.02594 | -1.10144 | 0.954081 |
| NEDD9 | DGVYDVPLHNPPDAK | Phospho(Y) | 32844.39 | 29088.68 | 30966.54 | 13457.76 | 14477.99 | 13967.88 | 27156.85 | 36147.32 | 31652.09 | 25569.5 | 22517.38 | 24043.44 | 0.031591 | -0.39666 | 0.783531 |
| NEDD9 | LYQVPNPQAAPR | Phospho(Y) | 3188.31 | 3598.47 | 3393.39 | 958.28 | 1398.95 | 1178.615 | 3177.95 | 4528.5 | 3853.225 | 1594.1 | 1858.95 | 1726.525 | 0.183339 | -1.1582 | 0.550779 |
| NEDD9 | DVYDIPPSHTTQGVYDIPPSSAK | Phospho(Y) | 1113.76 | 1148.78 | 1131.27 | 555.13 | 408.95 | 482.04 | 1168.67 | 1842.48 | 1505.575 | 633.36 | 545.74 | 589.55 | 0.412371 | -1.35263 | 0.290461 |
| NEDD9 | HQSLSPNHPPPQLGQSVGSQNDAYDVPR | Phospho(Y) | 32723.95 | 26119.94 | 29421.95 | 12822.89 | 17624.95 | 15223.92 | 26212.71 | 39881.7 | 33047.21 | 15432.03 | 18224.88 | 16828.46 | 0.167636 | -0.97363 | 0.144563 |
| NEDD9 | QAGRPDLRPEGVYDIPPTCTKPAGK | Phospho(Y) | 16911.57 | 20295.58 | 18603.58 | 7697.09 | 7006.25 | 7351.67 | 13680.24 | 17972.66 | 15826.45 | 8309.64 | 6821.36 | 7565.5 | -0.23324 | -1.06483 | 0.041363 |
| NEDD9 | ANPQERDGVYDVPLHNPPDAK | Phospho(Y) | 4085.58 | 5068.46 | 4577.02 | 685.5 | 1100.55 | 893.025 | 2582.42 | 3197.93 | 2890.175 | 64.03 | 1190.91 | 627.47 | -0.66325 | -2.20354 | -0.50915 |
| NEDD9 | GPVFSVPVGEIKPQGVYDIPPTK | Phospho(Y) | 6552.77 | 9726.22 | 8139.495 | 4116.85 | 4117.02 | 4116.935 | 5284.25 | 6074.78 | 5679.515 | 2323.24 | 2888.72 | 2605.98 | -0.51917 | -1.12394 | -0.65974 |
| NMD3 | LISQDIHSNTYNYK | Phospho(Y) | 0 | 846.81 | 423.405 | 342.66 | 506.71 | 424.685 | 0 | 1 | 0.5 | 158.22 | 4.04 | 81.13 | -9.72589 | 7.342164 | -2.38809 |
| NT5C2 | VDAYGNLLVCAHGFNFIR | Phospho(Y) | 41.01 | 936.67 | 488.84 | 182.29 | 51.57 | 116.93 | 133.36 | 1085.22 | 609.29 | 262.13 | 483.62 | 372.875 | 0.317767 | -0.70844 | 1.673047 |

| | | | | | | | | | | | | | | | | | |
|---|---|---|---|---|---|---|---|---|---|---|---|---|---|---|---|---|---|
| NUDCD2 | ENPGFDFSGAEISGNYTK | Phospho(Y) | 1631.28 | 1232.08 | 1431.68 | 443.67 | 475.17 | 459.42 | 1879.89 | 2155.88 | 2017.885 | 1025.32 | 1652.05 | 1338.685 | 0.495135 | -0.59203 | 1.542931 |
| NUMA1 | VESLESLYFTPIPAR | Phospho(Y) | 10623.98 | 13911.68 | 12267.83 | 3450.63 | 3606.77 | 3528.7 | 9599.05 | 13298.55 | 11448.8 | 9157.24 | 10352.45 | 9754.845 | -0.09968 | -0.23101 | 1.466982 |
| PAPSS2 | STNVVYQAHHVSR | Phospho(Y) | 193.57 | 395.2 | 294.385 | 164.7 | 40.7 | 102.7 | 0 | 29.33 | 14.665 | 209.93 | 254.88 | 232.405 | -4.32726 | 3.986192 | 1.178205 |
| PARD3 | ISHSLYSGIEGLDESPSR | Phospho(Y) | 1083.73 | 1961.98 | 1522.855 | 470.29 | 889.42 | 679.855 | 3263.22 | 4660.31 | 3961.765 | 4200.41 | 4384.18 | 4292.295 | 1.379365 | 0.115606 | 2.65845 |
| PARD3 | ERDYAEIQDFHR | Phospho(Y) | 2053.57 | 3095.84 | 2574.705 | 833.56 | 975.29 | 904.425 | 3248.07 | 4050.11 | 3649.09 | 3080.21 | 2922.37 | 3001.29 | 0.50313 | -0.28195 | 1.73051 |
| PARD3 | DVTIGGSAPIYVK | Phospho(Y) | 420.74 | 802.92 | 611.83 | 179.87 | 259.37 | 219.62 | 767.75 | 1576.69 | 1172.22 | 893.24 | 1019.78 | 956.51 | 0.938041 | -0.29339 | 2.122771 |
| PARD6B | KEEADYSAFGTDTLIK | Phospho(Y) | 2537.85 | 4004.52 | 3271.185 | 1973.94 | 2155.85 | 2064.895 | 4586.11 | 6767.57 | 5676.84 | 11496.28 | 14040.07 | 12768.18 | 0.795275 | 1.169392 | 2.628412 |
| PDAP1 | ARQYTSPEEIDAQLQAEK | Phospho(Y) | 0 | 13.24 | 6.62 | 146.2 | 29.72 | 87.96 | 0 | 0 | 0 | 866.96 | 44.02 | 455.49 | #NUM! | #DIV/0! | 2.3725 |
| PDLIM1 | VITNQYNNPAGLYSSENISNFNNALESK | Phospho(Y) | 447.39 | 1411.35 | 929.37 | 540.83 | 652.28 | 596.555 | 691.15 | 659.85 | 675.5 | 1706.83 | 840.82 | 1273.825 | -0.4603 | 0.915139 | 1.09444 |
| PGK1 | KELNYFAK | Phospho(Y) | 4110.49 | 5047.24 | 4578.865 | 1495.29 | 1365.96 | 1430.625 | 1201.37 | 1731.04 | 1466.205 | 4585.46 | 4309.28 | 4447.37 | -1.6429 | 1.600866 | 1.636307 |
| PGRMC1 | FYGPEGPYGVFAGR | Phospho(Y) | 1822.65 | 2687.76 | 2255.205 | 622.4 | 732.29 | 677.345 | 864.53 | 1107.55 | 986.04 | 1896.53 | 2254.56 | 2075.545 | -1.19354 | 1.073772 | 1.615527 |
| PHPT1 | IHVYGYSMAYGPAQHAISTEK | Phospho(Y) | 1596.72 | 2174.61 | 1885.665 | 1039.45 | 1360.91 | 1200.18 | 1503.07 | 2419.09 | 1961.08 | 2540.61 | 2586.55 | 2563.58 | 0.056575 | 0.386512 | 1.094909 |
| PIH1D1, Y194 | IQELGDLYTPAPGR | Phospho(Y) | 375.87 | 396.18 | 386.025 | 58.27 | 18.89 | 38.58 | 531.3 | 343.05 | 437.175 | 570.07 | 33.96 | 302.015 | 0.179517 | -0.53359 | 2.968695 |
| PIK3CA | EAGFSYSHAGLSNR | Phospho(Y) | 2411.07 | 2832.02 | 2621.545 | 369.05 | 313.34 | 341.195 | 7074.22 | 7601.49 | 7337.855 | 2055.54 | 2239.4 | 2147.47 | 1.484941 | -1.77272 | 2.65397 |
| PIK3R1 | DQYLMWLTQK | Phospho(Y) | 23577.15 | 26594.64 | 25085.9 | 8957.65 | 9035.32 | 8996.485 | 27630.77 | 34401.91 | 31016.34 | 22140.84 | 24537.7 | 23339.27 | 0.306152 | -0.41027 | 1.375326 |
| PIK3R1 | TRDQYLMWLTQK | Phospho(Y) | 1325.43 | 1786.69 | 1556.06 | 13.64 | 404.82 | 209.23 | 2414.99 | 4301.23 | 3358.11 | 185.86 | 1847.46 | 1016.66 | 1.109752 | -1.72381 | 2.280676 |
| PIK3R2 | DQYLVWLTQK | Phospho(Y) | 182.84 | 0 | 91.42 | 0 | 74.88 | 37.44 | 500.42 | 510.01 | 505.215 | 0 | 51.63 | 25.815 | 2.466316 | -4.29062 | -0.53637 |
| PIK3R2, Y464 | EYDQLYEEYTR | Phospho(Y) | 512.76 | 495.15 | 503.955 | 96.15 | 0.02 | 48.085 | 891.7 | 1002.87 | 947.285 | 399.02 | 292.28 | 345.65 | 0.910504 | -1.45449 | 2.845653 |
| PIN4 | FGYHIIMVEGR | Phospho(Y) | 5154.23 | 6854.81 | 6004.52 | 4004.48 | 3734.68 | 3869.58 | 18182.58 | 23917.74 | 21050.16 | 14193.21 | 15897.65 | 15045.43 | 1.80971 | -0.48451 | 1.959076 |
| PKM | EAEAAIYHLQLFEELR | Phospho(Y) | 39.07 | 488.91 | 263.99 | 287.2 | 48.01 | 167.605 | 92.96 | 817.41 | 455.185 | 52.14 | 585.31 | 318.725 | 0.78597 | -0.51414 | 0.927247 |
| PKP3 | NLIYDNADNK | Phospho(Y) | 17696.18 | 17658.08 | 17677.13 | 2788.78 | 2994.09 | 2891.435 | 1607.64 | 1785.39 | 1696.515 | 11853.27 | 10116.55 | 10984.91 | -3.38124 | 2.694877 | 1.925665 |
| PKP3 | GQYHTLQAGFSSR | Phospho(Y) | 10422.56 | 9043.74 | 9733.15 | 16822.79 | 16632.55 | 16727.67 | 20717.13 | 22037.3 | 21377.22 | 22141.07 | 24350.18 | 23245.63 | 1.135095 | 0.120885 | 0.474723 |
| PKP3 | ADYDTLSLR | Phospho(Y) | 2568.08 | 3193.94 | 2881.01 | 5247.46 | 4980.75 | 5114.105 | 4516.89 | 4262.75 | 4389.82 | 4453.29 | 7041.12 | 5747.205 | 0.607587 | 0.388699 | 0.168379 |
| PKP4 | NNYALNTTATYAEPYRPIQYR | Phospho(Y)Phosph | 13360.6 | 21266.58 | 17313.59 | 2111.52 | 3410.85 | 2761.185 | 8695.02 | 14271.49 | 11483.26 | 10956.68 | 17504.27 | 14230.48 | -0.59237 | 0.309452 | 2.365624 |
| PKP4 | VHFPASTDYSTQYGLK | Phospho(Y) | 9091.39 | 8876.13 | 8983.76 | 4635.78 | 4182.81 | 4409.295 | 15196.68 | 20508.17 | 17852.43 | 15348.68 | 17520.54 | 16434.61 | 0.990729 | -0.11938 | 1.898117 |
| PKP4 | SAVSPDLHITPIYEGR | Phospho(Y) | 12236.81 | 12300.51 | 12268.66 | 1018.82 | 4451.84 | 2735.33 | 13593.73 | 15120.08 | 14356.91 | 596.72 | 19035.79 | 9816.255 | 0.226767 | -0.5485 | 1.843458 |
| PKP4 | LQHQQLYYSQDDSNR | Phospho(Y) | 975.98 | 1332.37 | 1154.175 | 398.71 | 265.08 | 331.895 | 49.75 | 1624.34 | 837.045 | 650.98 | 1718.17 | 1184.575 | -0.46348 | 0.500992 | 1.835571 |
| PKP4 | NNYALNTTATYAEPYRPIQYR | Phospho(Y) | 33.86 | 46.48 | 40.17 | 9400.45 | 12.92 | 4706.685 | 236.27 | 202.11 | 219.19 | 31269.47 | 0.07 | 15634.77 | 2.447992 | 6.156432 | 1.731975 |
| PKP4 | LQHQQLYYSQDDSNRK | Phospho(Y) | 18435.08 | 23116.12 | 20775.6 | 4591.74 | 8352.59 | 6472.165 | 11857.61 | 13360.62 | 12609.12 | 23232.07 | 15169.9 | 19200.99 | -0.72042 | 0.606713 | 1.56886 |
| PKP4 | TYYSPVYR | Phospho(Y) | 592.05 | 630.62 | 611.335 | 455.66 | 520.63 | 488.145 | 116.35 | 44.62 | 80.485 | 1157.26 | 1042.14 | 1099.7 | -2.92517 | 3.772246 | 1.171728 |
| PKP4 | STTNYVDFYSTK | Phospho(Y) | 1900.19 | 2477.33 | 2188.76 | 817.01 | 907.02 | 862.015 | 2079.09 | 2692.37 | 2385.73 | 1880.8 | 1755.92 | 1818.36 | 0.124317 | -0.39179 | 1.076853 |
| PLCG1 | NPGFYVEANPMPTFK | Phospho(Y) | 2058.26 | 4732.32 | 3395.29 | 1353.03 | 1827.2 | 1590.115 | 3744 | 5685.11 | 4714.555 | 2267.35 | 3019.77 | 2643.56 | 0.473587 | -0.83464 | 0.733351 |
| PLEKHA5 | GGNRPNTGPLYTEADR | Phospho(Y) | 6190.21 | 7156.74 | 6673.475 | 1568.19 | 2052.5 | 1810.345 | 7216.31 | 8092.07 | 7654.19 | 7624.37 | 6580.23 | 7102.3 | 0.197812 | -0.10796 | 1.972022 |
| PLEKHA6 | LPPRSEDIYADPAAYVMR | Phospho(Y) | 133.65 | 893.92 | 513.785 | 31.9 | 0 | 15.95 | 0 | 68.25 | 34.125 | 136.17 | 25.67 | 80.92 | -3.91226 | 1.245667 | 2.34294 |
| PLEKHA6 | SEDIYADPAAYVMR | Phospho(Y) | 16790.94 | 15466.79 | 16128.87 | 3674.3 | 5504.14 | 4589.22 | 22112.92 | 26937.19 | 24525.06 | 13402.42 | 17905.54 | 15653.98 | 0.604611 | -0.64773 | 1.770209 |
| PLEKHA7 | QGPGQSLSFPENYQTLPK | Phospho(Y) | 534.04 | 888.47 | 711.255 | 1650.74 | 2280.36 | 1965.55 | 745.34 | 63.78 | 404.56 | 593.45 | 34.04 | 313.745 | -0.81401 | -0.36676 | -2.64727 |
| POF1B | GSHFFPGNNVIYEK | Phospho(Y) | 2148.12 | 2123.75 | 2135.935 | 284.7 | 427.03 | 355.865 | 2245.4 | 3053.09 | 2649.245 | 2431.64 | 3380.08 | 2905.86 | 0.310714 | 0.133384 | 3.029563 |
| PPDPF | AAIPSSGSLVATHDYYR | Phospho(Y) | 1889.39 | 1073.07 | 1481.23 | 47.48 | 156.13 | 101.805 | 1946.51 | 3112.33 | 2529.42 | 4.46 | 284.73 | 144.595 | 0.772011 | -4.12872 | 0.506209 |
| PPP1R12A | LAYVAPTIPR | Phospho(Y) | 1569.37 | 1656.89 | 1613.13 | 592.71 | 419.9 | 506.305 | 959.21 | 1603 | 1281.105 | 2076.62 | 1968.02 | 2022.32 | -0.33247 | 0.658623 | 1.997933 |
| PRKD2 | SVVGTPAYLAPEVLLNQGYNR | Phospho(Y) | 3127.05 | 3415.73 | 3271.39 | 1802.23 | 1940.78 | 1871.505 | 3396.74 | 2906.17 | 3151.455 | 2472.31 | 3456.13 | 2964.22 | -0.05389 | -0.08837 | 0.663454 |
| PRLR | LQLGGLDYLDPACFTHSFH | Phospho(Y) | 3350.01 | 8564.99 | 5957.5 | 190.11 | 206.07 | 198.09 | 1626.7 | 2202.61 | 1914.655 | 400.42 | 768.23 | 584.325 | -1.63762 | -1.71224 | 1.560615 |
| PRPF4B | LCDFGSASHVADNDITPYLVSR | Phospho(Y) | 2270233 | 3094679 | 2682456 | 2234156 | 2284797 | 2259476 | 2187429 | 2907725 | 2547577 | 2726831 | 2882988 | 2804909 | -0.07443 | 0.138828 | 0.311966 |
| PSMA2 | LAQQYYLVYQEPIPTAQLVQR | Phospho(Y) | 1638.43 | 1613.83 | 1626.13 | 569.14 | 479.18 | 524.16 | 1278.45 | 750.69 | 1014.57 | 1245.79 | 1808.18 | 1526.985 | -0.68057 | 0.589817 | 1.542607 |
| PSMC1 | KQEGTPEGLYL | Phospho(Y) | 238.66 | 450.58 | 344.62 | 0 | 123.94 | 61.97 | 562.48 | 22.08 | 292.28 | 27.22 | 0 | 13.61 | -0.23766 | -4.42461 | -2.1869 |
| PTBP1 | GQPIYIQFSNHK | Phospho(Y) | 1260.41 | 1090.14 | 1175.275 | 855 | 549.08 | 702.04 | 1765.56 | 1740.99 | 1753.275 | 1886.95 | 1616.33 | 1751.64 | 0.577054 | -0.00135 | 1.319081 |
| PTK2 | FLIGNQHIYQPVGKPDPAAPPK | Phospho(Y) | 9001.25 | 6945.28 | 7973.265 | 1926.55 | 2188.12 | 2057.335 | 8360.61 | 12064.72 | 10212.67 | 6181.27 | 6703.5 | 6442.385 | 0.357117 | -0.66469 | 1.646818 |
| PTK2 | GSIDREDGSLQGPIGNQHIYQPVGKPDPAAPPK | Phospho(Y) | 31158.74 | 37711.1 | 34434.92 | 5383.5 | 7552.72 | 6468.11 | 28611.55 | 30478.9 | 29545.23 | 25314.79 | 31460.44 | 28387.62 | -0.22095 | -0.05766 | 2.133846 |
| PTK2 | AAAYLDPNLNHTPNSSTK | Phospho(Y) | 969.07 | 944.62 | 956.845 | 191.4 | 439.65 | 315.525 | 1252.44 | 1260.39 | 1256.415 | 975.97 | 1447.32 | 1211.645 | 0.392956 | -0.05235 | 1.941141 |
| PTK2 | EDGSLQGPIGNQHIYQPVGKPDPAAPPK | Phospho(Y) | 6786.32 | 7420.6 | 7103.46 | 355.99 | 2682.84 | 1519.415 | 12479.67 | 379.3 | 6429.485 | 0 | 302.65 | 151.325 | -0.14382 | -5.40898 | -3.32779 |
| PTK6, Y447 | LSSFTSYENPT | Phospho(Y) | 1154.79 | 795.95 | 975.37 | 137.65 | 144.28 | 140.965 | 971.08 | 1019.31 | 995.195 | 1388.98 | 1110.3 | 1249.64 | 0.02903 | 0.328461 | 3.148104 |
| PTPN11 | IQNTGDYYDLYGGEK | Phospho(Y) | 32276.32 | 32628.85 | 32452.59 | 16498.24 | 19676.24 | 18087.24 | 61146.86 | 75338.53 | 68242.7 | 97641.26 | 118131.9 | 107886.6 | 1.072341 | 0.660769 | 2.576471 |
| PTPN11 | TIQMVRSQRSGMVQTEAQYR | Phospho(ST)Phosp | 728720.3 | 229067.6 | 478893.9 | 68880.48 | 111190.6 | 90035.52 | 383085.4 | 473908.6 | 428497 | 110205.8 | 455832.2 | 283019 | -0.16042 | -0.59839 | 1.652333 |
| PTPN11 | VYENVGLMQQQK | Phospho(Y) | 1165.52 | 1196.72 | 1181.12 | 1441.73 | 1826.46 | 1634.095 | 541.34 | 8.74 | 275.04 | 3767.67 | 119.24 | 1943.455 | -2.10244 | 2.82091 | 0.250132 |
| PTPRA | VVQEYIDAFSDYANFK | Phospho(Y) | 67791.49 | 91991.71 | 79891.6 | 49650.43 | 48448.9 | 49049.67 | 86872.25 | 114709.1 | 100790.7 | 49629.46 | 53623.97 | 51626.72 | 0.335247 | -0.96517 | 0.073875 |
| PTPRE | VVQDFIDIFSDYANFK | Phospho(Y) | 38351.81 | 50640.15 | 44495.98 | 10886.07 | 16736.26 | 13811.17 | 64095.33 | 108447.6 | 86271.45 | 39037.38 | 52165.29 | 45601.34 | 0.955208 | -0.91981 | 1.723241 |
| PTPRJ, Y1311 | VDLIYQNTTAMTIYENLAPVTTFGK | Phospho(Y) | 5440.01 | 9449.12 | 7444.565 | 1198.73 | 1075.74 | 1137.235 | 8385.69 | 11441.83 | 9913.76 | 6197.76 | 11523.68 | 8860.72 | 0.413245 | -0.16201 | 2.961894 |
| PTPRK | YLCEGTESPYQTGQLHPAIR | Phospho(Y) | 6775.61 | 8913.14 | 7844.375 | 1876.66 | 1802.46 | 1839.56 | 3405.23 | 3966.9 | 3686.065 | 4170.99 | 5405.47 | 4788.23 | -1.08958 | 0.377411 | 1.380132 |
| PTTG1IP | YGLFKEENPYAR | Phospho(Y) | 16961.32 | 15324.74 | 16143.03 | 3956.62 | 3515.97 | 3736.295 | 12671.76 | 17622.17 | 15146.97 | 20731.77 | 17506.7 | 19119.24 | -0.09188 | 0.335996 | 2.355345 |
| PVRL2 | EQTLQGAEEDEDLEGPPSYKPPTPK | Phospho(Y)Phosph | 2004.04 | 2254.32 | 2129.18 | 180.97 | 507.35 | 344.16 | 3385.4 | 3934.98 | 3660.19 | 2213.23 | 3980.39 | 3096.81 | 0.781621 | -0.24114 | 3.169632 |
| PVRL2 | EQTLQGAEEDEDLEGPPSYKPPTPK | Phospho(Y) | 3748.27 | 5763.05 | 4755.66 | 1348.25 | 1279.54 | 1313.895 | 6480.56 | 10443.52 | 8462.04 | 6891.83 | 5959.17 | 6425.5 | 0.83136 | -0.3972 | 2.289959 |
| PVRL2 | INPIYDALSYSSPSDSYQGK | Phospho(Y) | 968.66 | 1408.94 | 1188.8 | 397.41 | 961.08 | 679.245 | 3102.88 | 3651.43 | 3377.155 | 3040.2 | 2741.73 | 2890.965 | 1.506302 | -0.22426 | 2.089547 |
| PVRL2 | YHELPTLEER | Phospho(Y) | 3481.74 | 4001.14 | 3741.44 | 1447.51 | 1266.39 | 1356.95 | 5253.42 | 5938.6 | 5596.01 | 5449.74 | 4101.49 | 4775.615 | 0.580805 | -0.22871 | 1.815319 |
| PXN | VGEEEHVYSFPNK | Phospho(Y) | 335313.3 | 330081.5 | 332697.4 | 76321.06 | 78820.06 | 77570.56 | 400527.5 | 473958.8 | 437243.2 | 289538 | 274571.5 | 282054.8 | 0.394225 | -0.63246 | 1.862394 |
| PXN | FIHQQPQSSSPVYGSSAK | Phospho(Y) | 18467.2 | 18629.12 | 18548.16 | 4435.92 | 5227.66 | 4831.79 | 20644.71 | 22708.45 | 21676.58 | 8767.43 | 10398.36 | 9582.895 | 0.224861 | -1.1776 | 0.987904 |
| PYGB | ARPEYMLPVHFYGR | Phospho(Y) | 308.07 | 947.5 | 627.785 | 141.55 | 32.42 | 86.985 | 0 | 92.06 | 46.03 | 49.17 | 114.92 | 82.045 | -3.76962 | 0.833841 | -0.08435 |
| RAB13 | AYDHLFK | Phospho(Y) | 2644.73 | 3678.11 | 3161.42 | 875.63 | 853.71 | 864.67 | 285.44 | 812.31 | 548.875 | 1171.45 | 1025.22 | 1098.335 | -2.52602 | 1.000769 | 0.345097 |
| RAB1B | MNPEYDYLFK | Phospho(Y) | 4344.6 | 4762 | 4553.3 | 9517.13 | 10317.57 | 9917.35 | 8561.54 | 9569.93 | 9065.735 | 8776.91 | 10054.3 | 9415.605 | 0.993511 | 0.05463 | -0.0749 |
| RAB2A | AYAYLFK | Phospho(Y) | 1307.78 | 1608.17 | 1457.975 | 891.53 | 944.28 | 917.905 | 1245.57 | 1451.09 | 1348.33 | 520.41 | 513.08 | 516.745 | -0.11279 | -1.38365 | -0.82889 |
| RAB7A | QETEVELYNEFPEPIK | Phospho(Y) | 8323.59 | 10903.87 | 9613.73 | 4554.47 | 6010.56 | 5282.515 | 13675 | 16269.72 | 14972.36 | 8572.86 | 11218.79 | 9895.825 | 0.639133 | -0.59741 | 0.905595 |
| RAB7A | NALKQETEVELYNEFPEPIK | Phospho(Y) | 4378.03 | 7195.37 | 5786.7 | 2938.74 | 4370.46 | 3654.6 | 1561.51 | 2739.26 | 2150.385 | 2116.8 | 3019.69 | 2568.245 | -1.42815 | 0.256188 | -0.50893 |
| RAB9A | KEFIYYADVKEPESFPFVILGNK | Phospho(Y) | 3685.6 | 4302.37 | 3993.985 | 917.4 | 1538.84 | 1228.12 | 1535.19 | 3007.23 | 2271.21 | 1967.37 | 2352.17 | 2159.77 | -0.81437 | -0.07258 | 0.814426 |
| RAB9A | EFIYYADVKEPESFPFVILGNK | Phospho(Y) | 119.94 | 1890.73 | 1005.335 | 168.95 | 253.76 | 211.355 | 258.84 | 25.69 | 142.265 | 254.41 | 460.98 | 357.695 | -2.82102 | 1.330149 | 0.759062 |
| RASAL2 | RPLHPALNQPGGLQPLSFQNPVYHLNNPIPAMPK | Phospho(Y) | 8524.73 | 12571.91 | 10548.32 | 4079.22 | 3335.92 | 3707.57 | 8259.83 | 9541.79 | 8900.81 | 6577.12 | 6457.91 | 6517.515 | -0.245 | -0.44961 | 0.813848 |
| RAVER1 | IPLNPYLNLHSLLPASNLAGK | Phospho(Y) | 408.07 | 1009.34 | 708.705 | 1078.66 | 1351.22 | 1214.94 | 409.65 | 683.58 | 546.615 | 501.68 | 522.02 | 511.85 | -0.37466 | -0.0948 | -1.24709 |
| RBM14 | ASYVAPLTAQPATYR | Phospho(Y) | 10095.68 | 11530.47 | 10813.08 | 16042.66 | 15837.72 | 15940.19 | 7565.24 | 7666.71 | 7615.975 | 18687.93 | 15093.23 | 16890.58 | -0.50568 | 1.149118 | 0.08355 |
| RBM14 | YSGSYNDYLR | Phospho(Y) | 10.97 | 32.12 | 21.545 | 799.55 | 997.97 | 898.76 | 1.37 | 0 | 0.685 | 47.72 | 314.69 | 181.205 | -4.97511 | 8.047303 | -2.31031 |
| RBM14/RBM4 fusion | HLLPTSGAAATAAAAAAAAAAVTAASTSYYGR | Phospho(ST) | 866.01 | 1663.72 | 1264.865 | 1541.01 | 1762.19 | 1651.6 | 740.06 | 658.82 | 699.44 | 749.36 | 884.1 | 816.73 | -0.85471 | 0.223659 | -1.01593 |
| REPS2 | KDVLYSQPPSKPIR | Phospho(Y) | 1882.21 | 1735.91 | 1809.06 | 736.15 | 1121.5 | 928.825 | 1224.9 | 1434.16 | 1329.53 | 911.19 | 1312.33 | 1111.76 | -0.44432 | -0.25807 | 0.259367 |
| RET | YPNDSVYANWMLSPSAAK | Phospho(Y) | 9637.03 | 9402.86 | 9519.945 | 162.73 | 737.45 | 450.09 | 12169.84 | 12904.14 | 12536.99 | 940.07 | 2550.69 | 1745.38 | 0.397166 | -2.84458 | 1.955256 |
| RET | RPAQAFPVSYSSSGAR | Phospho(Y) | 51514.44 | 92917.67 | 72216.06 | 18133.2 | 25133.52 | 21633.36 | 121338.3 | 154028.6 | 137683.4 | 73085.79 | 72302.81 | 72694.3 | 0.930963 | -0.92144 | 1.748584 |
| RIN1 | EKPAQDPLYDVPNASGGQAGGPQRPGR | Phospho(Y) | 89509.44 | 99802.69 | 94656.07 | 55957.43 | 61539.58 | 58748.51 | 86157.2 | 107037.6 | 96597.39 | 114773.5 | 147875 | 131324.2 | 0.029289 | 0.443077 | 1.160509 |
| RIPK1 | LQDEANYHLYGSR | Phospho(Y) | 2.28 | 0 | 1.14 | 0 | 10.15 | 5.075 | 0.87 | 0 | 0.435 | 12.77 | 251.41 | 132.09 | -1.38995 | 8.24629 | 4.70197 |
| ROBO1 | QEVAPVQYNIVEQNK | Phospho(Y) | 70.09 | 20.96 | 45.525 | 241.88 | 183.22 | 212.55 | 97.16 | 59.13 | 78.145 | 0 | 36.91 | 18.455 | 0.779495 | -2.08214 | -3.52572 |
| RP11-114F7.3 | HPEIVGYSVPGR | Phospho(Y) | 2029.49 | 2038.82 | 2034.155 | 572.4 | 606.51 | 589.455 | 1824.08 | 3231.73 | 2527.905 | 3654.18 | 2619.88 | 3137.03 | 0.313513 | 0.311457 | 2.411946 |
| RPL31 | LYTLVTYVPVTTFK | Phospho(Y) | 5080.3 | 6066.98 | 5573.64 | 3093.13 | 3434.77 | 3263.95 | 7362.58 | 9246.75 | 8304.665 | 8539.65 | 8151.15 | 8345.4 | 0.575302 | 0.007059 | 1.354362 |
| RPLP0 | IIQLLDDYPK | Phospho(Y) | 2082.56 | 2848.65 | 2465.605 | 431.22 | 452.68 | 441.95 | 2255.05 | 3284.99 | 2770.02 | 3148.32 | 3504.38 | 3326.35 | 0.167955 | 0.264044 | 2.911985 |
| RPS2 | AFVAIGDYNGHVGLGVK | Phospho(Y) | 2745.56 | 4411.58 | 3578.57 | 577.47 | 767.68 | 672.575 | 1726.27 | 2910.48 | 2318.375 | 607.04 | 2285.07 | 1446.055 | -0.62627 | -0.68099 | 1.104355 |
| RPS27L | LVQSPNSYFMDVK | Phospho(Y) | 12938.89 | 14286.8 | 13612.85 | 10142.71 | 10779.48 | 10461.1 | 18947.37 | 23354.07 | 21150.72 | 25946.27 | 32634.51 | 29290.39 | 0.635738 | 0.469721 | 1.485394 |
| RPS29 | GHQQLYWSHPR | Phospho(Y) | 414.99 | 397.16 | 406.075 | 264.84 | 216.86 | 240.85 | 14.57 | 540.22 | 277.395 | 1002.1 | 259.29 | 630.695 | -0.5498 | 1.185001 | 1.388808 |
| RPS3 | FVDGLMIHSGDPVNYYVDTAVR | Phospho(Y) | 40.18 | 977.86 | 509.02 | 433.76 | 476.63 | 455.195 | 278.26 | 1572.51 | 925.385 | 1275.25 | 1296.86 | 1286.055 | 0.862331 | 0.474827 | 1.498396 |
| RPS6KB1 | DGFYPAPDFR | Phospho(Y) | 4261.86 | 4192.68 | 4227.27 | 9864.59 | 12328.09 | 11096.34 | 5196.14 | 5813.82 | 5504.98 | 23834.49 | 30563.48 | 27198.99 | 0.381011 | 2.304744 | 1.293469 |
| RPSAP58 | ADHQPLTEASYVNLPTIALCNTDSPLR | Phospho(ST) | 3577.92 | 4245.43 | 3911.675 | 1656.21 | 2065.02 | 1860.615 | 3556.38 | 4552.83 | 4054.605 | 3848.42 | 4536.56 | 4192.49 | 0.051775 | 0.048246 | 1.172028 |
| SCAMP3 | EPPPAYEPPAPAPLPPPSAPSLQPSR | Phospho(Y) | 5921.63 | 5516.49 | 5719.06 | 1070.27 | 1239.48 | 1154.875 | 7790.12 | 7592.83 | 7691.475 | 6998.68 | 9227.85 | 8113.265 | 0.427482 | 0.077022 | 2.812546 |
| SCAMP3 | QYATLDVYNPFETR | Phospho(Y) | 11780.76 | 13460.31 | 12620.54 | 8387.14 | 10813.26 | 9600.2 | 12877.05 | 15036.68 | 13956.87 | 13861.46 | 16346.73 | 15104.1 | 0.145202 | 0.113965 | 0.653803 |
| SCAMP3 | NYGSYSTQASAAAATAELLK | Phospho(Y) | 0 | 1721.72 | 860.86 | 644.93 | 1090.95 | 867.94 | 286.69 | 61.58 | 174.135 | 1499.79 | 243.92 | 871.855 | -2.30557 | 2.323882 | 0.006493 |
| SDC4 | KAPTNEFYA | Phospho(Y) | 13161.32 | 13498.35 | 13329.84 | 1148.95 | 913.05 | 1031 | 4020.66 | 4566.08 | 4293.37 | 4464.82 | 5177.56 | 4821.19 | -1.63448 | 0.167279 | 2.225345 |
| SDCBP | VIQAQTAFSANPANPAILSEASAPIPHDGNLYPR | Phospho(Y) | 11306.68 | 13947.52 | 12627.1 | 6017.17 | 6507.92 | 6262.545 | 9884 | 16429.48 | 13156.74 | 7546.8 | 7392.69 | 7469.745 | 0.059279 | -0.81667 | 0.25431 |
| SEC16A | ANHSSHQEDTYGALDFTLSR | Phospho(Y) | 2094.74 | 3383.99 | 2739.365 | 1265.59 | 1350.29 | 1307.94 | 1883.91 | 3616.54 | 2750.225 | 4289.51 | 5526.1 | 4907.805 | 0.005708 | 0.835528 | 1.907782 |
| SEC16A | YRPYDGAASAYAQNYR | Phospho(Y) | 1353.38 | 1999.19 | 1676.285 | 2324.14 | 2747.43 | 2535.785 | 259.75 | 258.52 | 259.135 | 561.52 | 1525.09 | 1043.305 | -2.69349 | 2.009385 | -1.28127 |
| SEPT2 | QQPTQFINPETPGYVGFANLPNQVHR | Phospho(Y) | 15970.1 | 19694.01 | 17832.06 | 12186.38 | 12607.21 | 12396.8 | 10966.59 | 15835.91 | 13401.25 | 13676.84 | 14528.25 | 14102.55 | -0.41211 | 0.073588 | 0.185988 |
| SEPT7 | NLEGYVGFANLPNQVYR | Phospho(Y) | 2013.91 | 2204.74 | 2109.325 | 602.97 | 446.84 | 524.905 | 1866.89 | 2162.27 | 2014.58 | 1842.85 | 1699.93 | 1771.39 | -0.0663 | -0.1856 | 1.754754 |
| SEPT7 | LAAVTYNGVDNNK | Phospho(Y) | 309.69 | 408.92 | 359.305 | 344.25 | 477.29 | 410.77 | 17.04 | 10.04 | 13.54 | 581.01 | 0 | 290.505 | -4.72991 | 4.423263 | -0.49977 |
| SET | IDFYFDENPYFENK | Phospho(Y) | 2204.73 | 3016.96 | 2610.845 | 594.95 | 720.59 | 657.77 | 2189.04 | 2446.86 | 2317.95 | 1475.93 | 2062.67 | 1769.3 | -0.17167 | -0.38967 | 1.427524 |
| SGK223 | EATQPEPIYAESTKR | Phospho(Y) | 183909.6 | 153099.2 | 168504.4 | 39127.34 | 38081.03 | 38604.19 | 105970.7 | 103039.8 | 104505.2 | 115935.9 | 106358.3 | 111147.1 | -0.68921 | 0.088895 | 1.525641 |
| SGK223 | LNLSHSETNVHDESHFSYSLSPGNR | Phospho(Y) | 1131.68 | 1841.16 | 1486.42 | 211.94 | 113.14 | 162.54 | 46.61 | 1139.22 | 592.915 | 781.05 | 18.22 | 399.635 | -1.32594 | -0.56914 | 1.297888 |
| SGK223 | EATQPEPIYAESTK | Phospho(Y) | 181406.9 | 202801.3 | 192104.1 | 75299.88 | 65587.5 | 70443.69 | 158168.2 | 140943.2 | 149555.7 | 166550 | 163898 | 165224 | -0.36121 | 0.143741 | 1.229881 |
| SH2B1 | LYLASHPQYAGPGAEAAFSR | Phospho(Y) | 1658.42 | 119.54 | 888.98 | 5007.66 | 484.17 | 2745.915 | 2823.53 | 4571.58 | 3697.555 | 7467.23 | 4635.14 | 6051.185 | 2.056349 | 0.710646 | 1.139931 |
| SHANK2 | GQMPENPYSEVGK | Phospho(Y) | 22.12 | 647.79 | 334.955 | 279.13 | 496.32 | 387.725 | 56.73 | 11.44 | 34.085 | 25.75 | 267.92 | 146.835 | -3.29676 | 2.106987 | -1.40084 |
| SHB | VTIADDYSDPFDAK | Phospho(Y) | 26264.24 | 26357.55 | 26310.9 | 8174.31 | 8267.56 | 8220.935 | 30210.5 | 41486.82 | 35848.66 | 19123.82 | 24095.36 | 21609.59 | 0.446259 | -0.73025 | 1.394297 |
| SHB | LDYCGGSGEPGGVQR | Phospho(Y) | 27160.36 | 29647.64 | 28404 | 5885.3 | 4203.43 | 5044.365 | 9469.56 | 9219.31 | 9344.435 | 11270.49 | 10882.23 | 11076.36 | -1.60391 | 0.245305 | 1.134739 |
| SHB | LPQDDDRPADEYDQPWEWNR | Phospho(Y) | 9043.28 | 10673.55 | 9858.415 | 1117.84 | 1958.14 | 1537.99 | 5124.3 | 8449.87 | 6787.085 | 1573.82 | 3976.2 | 2775.01 | -0.53856 | -1.2903 | 0.851447 |
| SHC1 | ELFDDPSYVNVQNLDK | Phospho(Y) | 91658.18 | 10471.05 | 51064.62 | 7664.96 | 12646.69 | 10155.83 | 92221.97 | 113994.2 | 103108.1 | 20866.92 | 122816.7 | 71841.82 | 1.013762 | -0.52126 | 2.822516 |
| SHC1 | ELFDDPSYVNVQNLDK | Phospho(Y) | 2365.99 | 104088.9 | 53227.45 | 65729.53 | 67315.09 | 66522.31 | 1780.01 | 2207.81 | 1993.91 | 92857.57 | 2839.6 | 47848.59 | -4.7385 | 4.584804 | -0.47536 |
| SHC4 | VDLFDDPCYINTQALQSTPGSAGNQR | Phospho(Y) | 19155.82 | 24983.18 | 22069.5 | 4159.94 | 3479.54 | 3819.74 | 22267.13 | 32052.77 | 27159.95 | 24666.49 | 27805.01 | 26235.75 | 0.299427 | -0.04995 | 2.779988 |
| SIGIRR | TDFYCLVSK | Phospho(Y) | 137.85 | 283.15 | 210.5 | 236.17 | 210.52 | 223.345 | 149.73 | 325.82 | 237.775 | 780.15 | 373.33 | 576.74 | 0.175777 | 1.278324 | 1.368647 |
| SIPA1L3 | LYSSGSSTPTGLAGGSRDPPR | Phospho(Y)Phosph | 1823.34 | 1931.47 | 1877.405 | 17436.5 | 15979.76 | 16708.13 | 10628.12 | 15636.64 | 13132.38 | 5729.05 | 4606.43 | 5167.74 | 2.806317 | -1.34552 | -1.69294 |
| SLC12A2 | GNHQSVLTFYS | Phospho(ST) | 1263.1 | 1521.92 | 1392.51 | 879.67 | 1184.19 | 1031.93 | 2145.1 | 2336.8 | 2240.95 | 39.33 | 2692.77 | 1366.05 | 0.686423 | -0.7141 | 0.404665 |
| SLC12A2 | EGAQYLMQAAGLGR | Phospho(Y) | 628.06 | 535.29 | 581.675 | 457.01 | 644.28 | 550.645 | 1447.89 | 2296.06 | 1871.975 | 507.28 | 766.2 | 636.74 | 1.686276 | -1.55578 | 0.209582 |
| SLC1A5 | HYRGPAGDATVASEK | Phospho(Y) | 1923.79 | 3143.54 | 2533.665 | 15.16 | 746.52 | 380.84 | 159.07 | 141.59 | 150.33 | 607.17 | 1109.27 | 858.22 | -4.07502 | 2.513215 | 1.172163 |
| SLC25A5 | AAYFGIYDTAK | Phospho(Y) | 1893.49 | 1960.53 | 1927.01 | 472.81 | 705.36 | 589.085 | 2721.71 | 3028.92 | 2875.315 | 2792.99 | 3640.87 | 3216.93 | 0.577356 | 0.161965 | 2.449137 |
| SLC25A6, Y191 | AAYFGVYDTAK | Phospho(Y) | 122.08 | 243.54 | 182.81 | 159.84 | 7.34 | 83.59 | 0 | 661.48 | 330.74 | 692.6 | 1077.84 | 885.22 | 0.855353 | 1.420339 | 3.404634 |
| SLC38A2 | SHYADVDPENQNFLLESNLGK | Phospho(ST) | 4693.74 | 5129.58 | 4911.66 | 437.48 | 1121.8 | 779.64 | 3685.3 | 4405.16 | 4045.23 | 759.7 | 4508.34 | 2634.02 | -0.27999 | -0.61896 | 1.756386 |
| SLITRK6 | ANLHAEPDYLEVLEQQT | Phospho(Y) | 3900.21 | 4157.81 | 4029.01 | 3268.38 | 3678.02 | 3473.2 | 6008.4 | 6472.04 | 6240.22 | 9699.62 | 10320.67 | 10010.15 | 0.631172 | 0.681794 | 1.527125 |
| SMC3 | LFYHIVDSDEVSTK | Phospho(Y) | 1341.51 | 1503.1 | 1422.305 | 1011.48 | 726.7 | 869.09 | 1893.59 | 1514.98 | 1704.285 | 2011.85 | 2477.71 | 2244.78 | 0.260936 | 0.397407 | 1.368997 |
| SMG9 | EGQRPTQPVYQIQNR | Phospho(Y) | 45.08 | 1316.79 | 680.935 | 254.4 | 316.84 | 285.62 | 178.64 | 641.25 | 409.945 | 1726.57 | 1565.1 | 1645.835 | -0.73209 | 2.005317 | 2.526651 |
| SNRNP70 | EFEVYGPIKR | Phospho(Y) | 2987.29 | 3630.16 | 3308.725 | 1301.76 | 2001.41 | 1651.585 | 4286.15 | 5538.73 | 4912.44 | 4422.69 | 4702.99 | 4562.84 | 0.570164 | -0.10651 | 1.466081 |
| SPTAN1 | QVEELYHSLLELGEK | Phospho(Y) | 822.69 | 902.23 | 862.46 | 57.87 | 191.99 | 124.93 | 858.8 | 23.01 | 440.905 | 916.93 | 1825.69 | 1371.31 | -0.96799 | 1.637015 | 3.456363 |
| SPTLC1 | DHPALNYNIVSGPPSHK | Phospho(Y) | 10871.5 | 14260.7 | 12566.1 | 611.14 | 632.34 | 621.74 | 14827.33 | 13037.25 | 13932.29 | 3691 | 5391.44 | 4541.22 | 0.148895 | -1.61728 | 2.868697 |
| SRCIN1 | EPLYAAFPGSHLTNGDLR | Phospho(Y) | 2454.59 | 2678.92 | 2566.755 | 420.64 | 648.45 | 534.545 | 2962.94 | 2458.11 | 2710.525 | 3439.34 | 3274.85 | 3357.095 | 0.078627 | 0.308641 | 2.65083 |
| SRCIN1 | KEPLYAAFPGSHLTNGDLR | Phospho(Y) | 9144.78 | 13455.38 | 11300.08 | 2742.84 | 2707.05 | 2724.945 | 8666.8 | 16160.57 | 12413.69 | 15910.29 | 15991.3 | 15950.8 | 0.135598 | 0.361697 | 2.549329 |
| SRCIN1 | GEGLYADPYGLLHEGR | Phospho(Y) | 12766.51 | 17268.71 | 15017.61 | 5956.87 | 3822.33 | 4889.6 | 11290.46 | 15858.47 | 13574.47 | 14171.51 | 14279.74 | 14225.63 | -0.14576 | 0.067597 | 1.540704 |
| ST6GALNAC2 | IDYVMLR | Phospho(Y) | 68.64 | 463.6 | 266.12 | 333.7 | 296.2 | 314.95 | 0 | 695.33 | 347.665 | 1791.78 | 2204.44 | 1998.11 | 0.385621 | 2.522866 | 2.665441 |
| STAM2 | LVNEAPVYSVYSK | Phospho(Y) | 2344.57 | 2656.27 | 2500.42 | 799.57 | 725.52 | 762.545 | 2121.37 | 2645.37 | 2383.37 | 3856.69 | 4326.66 | 4091.675 | -0.06917 | 0.779689 | 2.423797 |
| STAT3 | YCRPESQEHPEADPGAAPYLK | Phospho(Y) | 1188.17 | 2064.93 | 1626.55 | 779.01 | 572.01 | 675.51 | 19.53 | 1596.16 | 807.845 | 999.47 | 2165.9 | 1582.685 | -1.00966 | 0.970224 | 1.228325 |
| STAT3 | YCRPESQEHPEADPGSAAPYLK | Phospho(Y) | 6843.89 | 4919.46 | 5881.675 | 2671.02 | 3261.83 | 2966.425 | 1837.57 | 7719.83 | 4778.7 | 6816.18 | 7650.71 | 7233.445 | -0.29961 | 0.598065 | 1.285958 |
| STIP1 | LAYINPDLALEEK | Phospho(Y) | 1749.32 | 1847.14 | 1798.23 | 1552.63 | 1815.44 | 1684.035 | 1288.95 | 2064.05 | 1676.5 | 2094.55 | 2754.09 | 2424.32 | -0.10113 | 0.532128 | 0.525658 |
| STX12 | SYGPLDMYR | Phospho(Y) | 2295.83 | 2974.52 | 2635.175 | 1109.37 | 1290.41 | 1199.89 | 4780.7 | 6500.56 | 5640.63 | 7865.34 | 7457.77 | 7661.555 | 1.097958 | 0.441781 | 2.674735 |
| STX12 | SYGPLDMYRNPGPSGPQLR | Phospho(Y) | 2326.64 | 1737.43 | 2032.035 | 134.39 | 370.71 | 252.55 | 2253.69 | 2738.52 | 2496.105 | 1270.47 | 1791.34 | 1530.905 | 0.296753 | -0.70529 | 2.599744 |
| STX4, Y251 | NILSSADYVER | Phospho(Y) | 298.74 | 366.78 | 332.76 | 0 | 67.78 | 33.89 | 787.47 | 1044.66 | 916.065 | 131.77 | 764.5 | 448.135 | 1.460968 | -1.03152 | 3.725002 |
| STX7 | SYTPGVGGDPAQLAQR | Phospho(ST) | 907.47 | 962.25 | 934.86 | 297.73 | 313.24 | 305.485 | 1463.66 | 1552.26 | 1507.96 | 1088.09 | 1426.62 | 1257.355 | 0.689776 | -0.26221 | 2.041219 |
| SYAP1 | DFGNYLFNFASAATK | Phospho(Y) | 163.66 | 0 | 81.83 | 0 | 86.72 | 43.36 | 278.66 | 879.01 | 578.835 | 118.89 | 107.69 | 113.29 | 2.82245 | -2.35313 | 1.385584 |
| SYK | QESTVSFNPYEPELAPWAADKGPQR | Phospho(Y) | 42379.18 | 54688.59 | 48533.89 | 16527.68 | 18246.71 | 17387.2 | 46639.37 | 58243.12 | 52441.25 | 40552.58 | 44520.1 | 42536.34 | 0.11171 | -0.30201 | 1.290671 |
| SYK | QESTVSFNPYEPELAPWAADK | Phospho(Y) | 9769.99 | 10932.25 | 10351.12 | 4594.72 | 6485.4 | 5540.06 | 20109.11 | 24874.45 | 22491.78 | 11050.97 | 14184.13 | 12617.55 | 1.119611 | -0.83397 | 1.187458 |
| SYNCRIP | DYAFIHFDER | Phospho(Y) | 212.72 | 108.54 | 160.63 | 34.3 | 436.02 | 235.16 | 609.28 | 546.81 | 578.045 | 8.74 | 606.58 | 307.66 | 1.84744 | -0.90984 | 0.387694 |
| TBCA | LEAAYLDLQR | Phospho(Y) | 453.14 | 406.66 | 429.9 | 125.4 | 73.41 | 99.405 | 554.38 | 797.24 | 675.81 | 649.43 | 711.4 | 680.415 | 0.652617 | 0.009797 | 2.775025 |
| TFG | NRPPFGQGYTQPGPGYR | Phospho(Y) | 186.02 | 1453.37 | 819.695 | 1958.68 | 1730.23 | 1844.455 | 46.32 | 143.36 | 94.84 | 254.4 | 1201.53 | 727.965 | -3.11152 | 2.940302 | -1.34125 |
| TFRC | SAFSNLFGGEPLSYTR | Phospho(Y) | 20247.92 | 28098.87 | 24173.4 | 6823.08 | 7890.52 | 7356.8 | 23333.95 | 30899 | 27116.48 | 17623.84 | 18415.42 | 18019.63 | 0.16575 | -0.5896 | 1.292419 |
| THRAP3 | WEGLVYAPPGK | Phospho(Y) | 518.4 | 517.28 | 517.84 | 6.72 | 38 | 22.36 | 322.15 | 448.23 | 385.19 | 105.97 | 370.21 | 238.09 | -0.42694 | -0.69406 | 3.412515 |

| | | | | | | | | | | | | | | | | | |
|---|---|---|---|---|---|---|---|---|---|---|---|---|---|---|---|---|---|
| TJP1 | YEPIQATPPPPPLPSQYAQPSQPVTSASLHIHSK | Phospho(Y) | 0.01 | 1578.22 | 789.115 | 0 | 967.73 | 483.865 | 263.73 | 246.09 | 254.91 | 24.02 | 263.03 | 143.525 | -1.63025 | -0.82869 | -1.7533 |
| TJP2 | HPDIYAVPIK | Phospho(Y) | 27595 | 29061.03 | 28328.02 | 14182.88 | 16772.32 | 15477.6 | 25192.96 | 37003.94 | 31098.45 | 68659.48 | 49089.94 | 58874.71 | 0.134613 | 0.920805 | 1.927466 |
| TJP2 | IEIAQKHPDIYAVPIK | Phospho(Y) | 2181.37 | 2652.32 | 2416.845 | 29.39 | 205.37 | 117.38 | 362.25 | 507.48 | 434.865 | 41.05 | 1554.84 | 797.945 | -2.47449 | 0.875722 | 2.765103 |
| TLN1 | AVSSAIAQLLGEVAQGNENYAGIAAR | Phospho(Y) | 1112 | 2039.13 | 1575.565 | 812.46 | 874.6 | 843.53 | 1108.6 | 1828.02 | 1468.31 | 418.08 | 971.27 | 694.675 | -0.10171 | -1.07975 | -0.2801 |
| TLN1, Y71 | ALDYYMLR | Phospho(Y) | 838.21 | 947.39 | 892.8 | 46.76 | 69.75 | 58.255 | 1845.54 | 3619.58 | 2732.56 | 3104.72 | 2962.65 | 3033.685 | 1.613844 | 0.150818 | 5.702546 |
| TMEM106B | NGDVSQFPYVEFTGR | Phospho(Y) | 1928.57 | 1869.63 | 1899.1 | 623.39 | 746.95 | 685.17 | 892.99 | 1524.19 | 1208.59 | 953.2 | 1368.42 | 1160.81 | -0.65199 | -0.05819 | 0.760598 |
| TMEM127 | MYAPGGAGLPGGR | Phospho(Y) | 12142.1 | 9262.56 | 10702.33 | 1160.94 | 1187.58 | 1174.26 | 11324.08 | 14522.19 | 12923.14 | 4099.66 | 4571.2 | 4335.43 | 0.272031 | -1.57571 | 1.884423 |
| TMEM134 | LRYQNLENDEDGAQASPEPDGGVGTR | Phospho(Y) | 1686.73 | 1475.88 | 1581.305 | 675.43 | 772.39 | 723.91 | 2363.93 | 2291.25 | 2327.59 | 2260.72 | 1724.93 | 1992.825 | 0.557721 | -0.22402 | 1.460933 |
| TMEM192 | AKPEPDILEEEKIYAYPSNITSETGFR | Phospho(Y) | 3557.36 | 4569.11 | 4063.235 | 57.67 | 125.56 | 91.615 | 2181.49 | 2219.08 | 2200.285 | 3235.9 | 4000.54 | 3618.22 | -0.88494 | 0.71759 | 5.303552 |
| TMEM192 | IYAYPSNITSETGFR | Phospho(Y) | 3302.7 | 489.57 | 1896.135 | 1479.52 | 460.17 | 969.845 | 3146.43 | 15913.57 | 9530 | 14696.46 | 4530.38 | 9613.42 | 2.329415 | 0.012574 | 3.309224 |
| TMEM38B | IVGGVTHANSYYKNGWIVMIAIGWARGAGGTIITNFER | Phospho(ST)Phosp | 970.72 | 112.11 | 541.415 | 5884.69 | 6439.19 | 6161.94 | 10.57 | 1610.08 | 810.325 | 6287.01 | 28.91 | 3157.96 | 0.581766 | 1.96242 | -0.96439 |
| TNK2 | VSSTHYYLLPERPSYLER | Phospho(Y) | 19407.83 | 27405.26 | 23406.55 | 10592.15 | 10258.74 | 10425.45 | 20937.27 | 24488.61 | 22712.94 | 27132.9 | 25852.96 | 26492.93 | -0.0434 | 0.222093 | 1.345498 |
| TNK2 | KPTYDPVSEDQDPLSSDFKR | Phospho(Y) | 17824.39 | 22080.07 | 19952.23 | 14269.42 | 13795.84 | 14032.63 | 14958.84 | 26363.85 | 20661.35 | 33603.92 | 26692.53 | 30148.23 | 0.050384 | 0.545139 | 1.103288 |
| TNK2 | ALPQNDDHYVMQEHR | Phospho(Y) | 14724.48 | 17613.85 | 16169.17 | 5183.7 | 6382.24 | 5782.97 | 19308.01 | 20687.01 | 19997.51 | 16808.17 | 6493.06 | 11650.62 | 0.306575 | -0.77941 | 1.010524 |
| TNK2 | KPTYDPVSEDQDPLSSDFK | Phospho(Y) | 22431.81 | 24085.76 | 23258.79 | 17744.76 | 19856.58 | 18800.67 | 33035.19 | 43177.3 | 38106.25 | 30518.29 | 44946.8 | 37732.55 | 0.712252 | -0.01422 | 1.005025 |
| TNK2 | YATPQVIQAPGPR | Phospho(Y) | 1059.54 | 1009.83 | 1034.685 | 870.64 | 1244.03 | 1057.335 | 785.47 | 1087.99 | 936.73 | 7.49 | 1106.57 | 557.03 | -0.14349 | -0.74988 | -0.92461 |
| TNS3 | LSLGQYDNDAGGQLPFSK | Phospho(Y) | 2326.86 | 2902.37 | 2614.615 | 2552.87 | 2993.39 | 2773.13 | 2481.23 | 3432.73 | 2956.98 | 1989.75 | 3265.51 | 2627.63 | 0.177526 | -0.17036 | -0.07775 |
| TOM1L2 | TVTYEDPQAVGGLASALDNR | Phospho(Y) | 3328.46 | 3763.69 | 3546.075 | 4257.62 | 4514.05 | 4385.835 | 2982.85 | 3099.73 | 3041.29 | 5175.24 | 4913.05 | 5044.145 | -0.22154 | 0.729926 | 0.201758 |
| TOM1L2 | KTVTYEDPQAVGGLASALDNRK | Phospho(Y) | 4861.06 | 4759.47 | 4810.265 | 3347.74 | 3005.59 | 3176.665 | 2515.71 | 3076.46 | 2796.085 | 5331.47 | 5301.32 | 5316.395 | -0.78271 | 0.92704 | 0.742935 |
| TOM1L2 | KTVTYEDPQAVGGLASALDNR | Phospho(Y) | 7876.85 | 10576.82 | 9226.835 | 9320.5 | 10138.96 | 9729.73 | 8688.05 | 11613.56 | 10150.81 | 11116.85 | 16501.4 | 13809.13 | 0.137686 | 0.444028 | 0.50515 |
| TPCN1 | ELKPETISSPGYHILR | Phospho(Y) | 11.54 | 359.11 | 185.325 | 205.01 | 128.54 | 166.775 | 18.39 | 295.91 | 157.15 | 279.84 | 324.82 | 302.33 | -0.23792 | 0.943982 | 0.858221 |
| TRIM25 | FDTIYQILLK | Phospho(Y) | 775.4 | 930.17 | 852.785 | 398.61 | 359.35 | 378.98 | 774.94 | 1087.15 | 931.045 | 621.53 | 1199.08 | 910.305 | 0.126669 | -0.0325 | 1.264228 |
| TRIM28 | VFPGSTTEDYNLIVIER | Phospho(Y) | 960.23 | 1910.73 | 1435.48 | 78.85 | 133.05 | 105.95 | 1203.33 | 940.53 | 1071.93 | 732.35 | 918.13 | 825.24 | -0.42132 | -0.37733 | 2.96143 |
| TRIP6 | QAYEPPPPPAYR | Phospho(Y) | 3418.6 | 3602.72 | 3510.66 | 1228.08 | 1166.08 | 1197.08 | 3613.46 | 4081.37 | 3847.415 | 6525.23 | 6706.49 | 6615.86 | 0.132147 | 0.782039 | 2.466409 |
| TRIP6 | VNFCPLPSEQCYQAPGGPEDR | Phospho(Y) | 6825.62 | 7411.7 | 7118.66 | 1553.15 | 2657.88 | 2105.515 | 5581.82 | 6642.82 | 6112.32 | 5912.02 | 8915.18 | 7413.6 | -0.21989 | 0.278454 | 1.816001 |
| TUBA1C | VGINYQPPTVVPGGDLAK | Phospho(Y) | 2864.05 | 3039.7 | 2951.875 | 4360.87 | 4259.32 | 4310.095 | 4460.51 | 5346.92 | 4903.715 | 4311.02 | 3364.27 | 3837.645 | 0.732244 | -0.35365 | -0.1675 |
| TUBA1C | IHFPLATYAPVISAEK | Phospho(Y) | 3840.4 | 5141.02 | 4490.71 | 8395.85 | 7853.09 | 8124.47 | 3868.27 | 4232.39 | 4050.33 | 4033.49 | 4528.67 | 4281.08 | -0.1489 | 0.079935 | -0.9243 |
| TUBA1C | QLFHPEQLITGKEDAANNYAR | Phospho(Y) | 9.07 | 301.61 | 155.34 | 420.64 | 611.88 | 516.26 | 59.12 | 29.95 | 44.535 | 177.05 | 141.51 | 159.28 | -1.80242 | 1.838554 | -1.69653 |
| TUBA4A | FDLMYAK | Phospho(Y) | 529.14 | 727.11 | 628.125 | 340.11 | 305.84 | 322.975 | 224.63 | 142.53 | 183.58 | 671.49 | 1124.71 | 898.1 | -1.77464 | 2.290467 | 1.475454 |
| TUBB4B | INVYYNEATGGK | Phospho(Y) | 1449.5 | 1618.22 | 1533.86 | 854.3 | 1262.46 | 1058.38 | 1569.53 | 1971.11 | 1770.32 | 2902.12 | 2079.48 | 2490.8 | 0.206843 | 0.492599 | 1.234751 |
| TUBB4B | NSSYFVEWIPNNVK | Phospho(Y) | 3065.71 | 4463.75 | 3764.73 | 5442.1 | 5318.8 | 5380.45 | 4307.17 | 4067.84 | 4187.505 | 4672.71 | 4574.39 | 4623.55 | 0.153545 | 0.14291 | -0.21873 |
| TYK2 | LLAQAEGEPCYIR | Phospho(Y) | 118140.5 | 71076.62 | 94608.57 | 46442.42 | 47882.45 | 47162.44 | 77620.42 | 82071.63 | 79846.03 | 98285.81 | 103716.3 | 101001 | -0.24475 | 0.339078 | 1.09866 |
| UBAP2L | FPLDYYSIPFPTPTTPLTGR | Phospho(Y) | 328.77 | 2246.11 | 1287.44 | 782.38 | 1136.24 | 959.31 | 1703.87 | 235.53 | 969.7 | 1684.01 | 3979.48 | 2831.745 | -0.40889 | 1.546081 | 1.561622 |
| UBR5 | AASTAPSSTSTPAASSAGLIYIDPSNLR | Phospho(Y) | 1589.23 | 3026.76 | 2307.995 | 5474.93 | 3720.9 | 4597.915 | 1601.08 | 2338.78 | 1969.93 | 3320.46 | 3186.35 | 3253.405 | -0.2285 | 0.723806 | -0.49903 |
| UGDH | AVQALCAVYEHWVPR | Phospho(Y) | 398 | 0 | 199 | 21.06 | 15.52 | 18.29 | 246.44 | 475.29 | 360.865 | 0 | 445.89 | 222.945 | 0.858691 | -0.69477 | 3.607561 |
| UGDH | INAWNSPTLPIYEPGLK | Phospho(Y) | 1320.13 | 1820.03 | 1570.08 | 1517.15 | 1500.17 | 1508.66 | 2270.55 | 2909.21 | 2589.88 | 1897.25 | 1716.63 | 1806.94 | 0.722047 | -0.51934 | 0.260281 |
| USP9Y | FNDYFEFPR | Phospho(Y) | 65.29 | 463.07 | 264.18 | 128.14 | 211.63 | 169.885 | 0 | 1 | 0.5 | 291.55 | 55.28 | 173.415 | -9.04538 | 8.438085 | 0.02967 |
| VASP | VQIYHNPTANSFR | Phospho(Y) | 13689.34 | 14283.47 | 13986.41 | 3681.68 | 4882.89 | 4282.285 | 20377.36 | 20952.56 | 20664.96 | 27543.77 | 27563.63 | 27553.7 | 0.563161 | 0.415059 | 2.685793 |
| VTI1B | YGIYAVENEHMNR | Phospho(Y) | 6971.51 | 6747.16 | 6859.335 | 1245.09 | 931.72 | 1088.405 | 6816.23 | 6228.13 | 6522.18 | 3233.01 | 3598.32 | 3415.665 | -0.07271 | -0.93319 | 1.649951 |
| WASL | VIYDFIEK | Phospho(Y) | 57085.82 | 66965.58 | 62025.7 | 27756.28 | 25935.93 | 26846.11 | 36158.86 | 74845.98 | 55502.42 | 43509.87 | 41851 | 42680.44 | -0.16032 | -0.37898 | 0.668862 |
| WDR1 | AHDGGIYAISWSPDSTHLLSASGDK | Phospho(Y) | 0 | 1210.27 | 605.135 | 434.32 | 879.26 | 656.79 | 22.65 | 0 | 11.325 | 1055.23 | 73.99 | 564.61 | -5.73967 | 5.639672 | -0.21818 |
| YBX1 | RPQYSNPPVQGEVMEGADNQGAGEQGRPVR | Phospho(Y) | 497.62 | 554.73 | 526.175 | 929.31 | 785.12 | 857.215 | 2538.52 | 2825.84 | 2682.18 | 3452.03 | 1493.29 | 2472.66 | 2.349791 | -0.11734 | 1.528335 |
| YES1 | LDNGGYYITTR | Phospho(Y) | 2093.14 | 78.3 | 1085.72 | 89.89 | 48.61 | 69.25 | 1629.34 | 1452.6 | 1540.97 | 113.89 | 1787.26 | 950.575 | 0.505187 | -0.69697 | 3.778914 |
| YES1 | KLDNGGYYITTR | Phospho(Y) | 1145.77 | 1668.15 | 1406.96 | 420.64 | 458.65 | 439.645 | 891.89 | 61.58 | 476.735 | 2913.09 | 2465.5 | 2689.295 | -1.56132 | 2.495969 | 2.612817 |
| YES1 | KDPDERPTFEYIQSFLEDYFTATEPQYQPGENL | Phospho(Y) | 21517.96 | 102478.8 | 61998.36 | 8715.36 | 12751.16 | 10733.26 | 15124.53 | 5189.83 | 10157.18 | 29950.69 | 54071.63 | 42011.16 | -2.60973 | 2.048273 | 1.968684 |
| YES1 , Y416 | LIEDNEYTAR | Phospho(Y) | 903.62 | 1402.82 | 1153.22 | 0 | 0.9 | 0.45 | 208.91 | 36.71 | 122.81 | 2460.54 | 80.74 | 1270.64 | -3.23117 | 3.371055 | 11.46334 |
| ZDHHC5 | GVGSPEPGPTAPYLGR | Phospho(Y) | 420.09 | 591.76 | 505.925 | 1329.64 | 1922.1 | 1625.87 | 853.94 | 0.04 | 426.99 | 0 | 1587.7 | 793.85 | -0.24472 | 0.894664 | -1.03427 |
| ZNF185 | GALADYEGKDVATR | Phospho(Y) | 1792.59 | 1757.12 | 1774.855 | 368.64 | 616.92 | 492.78 | 1826.8 | 1681.38 | 1754.09 | 177.93 | 1699.33 | 938.63 | -0.01698 | -0.90209 | 0.929613 |
